# Maternal microbiome promotes offspring ovarian reserve via bile acid metabolism

**DOI:** 10.1038/s44319-026-00912-3

**Published:** 2026-08-27

**Authors:** Han Wang, Yukang Li, Yilin Niu, Xin Chen, Jing Liu, Weiting Feng, Jingru Cao, Haixia Liu, Chang Liu, Xiaoying Liang, Ziqing Ge, Junjie Wang, Xinlei Feng, Shunfeng Cheng, Wei Shen, Wei Ge

**Affiliations:** 1 College of Animal Science and Technology, Qingdao Agricultural University, Qingdao, 266109 China; 2 College of Life Sciences, Qingdao Agricultural University, Qingdao, 266109 China; 3 Central Laboratory of Qingdao Agricultural University, Qingdao Agricultural University, Qingdao, 266109 China; 4 Shandong Animal Products Quality and Safety Center, Jinan, 250100 China

**Keywords:** Development, Metabolism, Microbiology, Virology & Host Pathogen Interaction

## Abstract

The maternal microbiome plays a crucial role in host ovarian function and fertility, yet its influence on in-utero ovarian development in offspring remains poorly understood. Here, we demonstrate that maternal antibiotic-induced microbiome disruption in mice leads to diminished ovarian reserve (DOR) in the offspring, while fecal microbiota transplantation (FMT) alleviates this effect. Metabolomic analysis reveals that maternal microbiome disruption alters metabolomic profiles in the maternal serum, with pathway enrichment analysis indicating reduced bile acid secretion in the maternal serum of ABX dams. Importantly, supplementation of key bile acid metabolites and transplantation of *Lactobacillus gasseri* (*L. gasseri*), a known promoter of bile acid metabolism, to antibiotic-treated dams abrogated maternal microbiome disruption-induced DOR. Additionally, through in vitro fertilization assay, we found that maternal microbiome disruption impaired oocyte quality in offspring, while FMT and supplementation with bile acid metabolites alleviated this effect. Together, our findings highlight the critical role of the maternal microbiome in offspring fertility, potentially through microbially mediated bile acid metabolism in the maternal serum.

## Synopsis

The maternal microbiome has a critical role in regulating ovarian reserve and oocyte quality in offspring in mice. Maternal antibiotic-induced microbiome disruption leads to diminished ovarian reserve in the offspring, while fecal microbiota transplantation (FMT) and supplementation with bile acid metabolites alleviates this effect.

- Maternal antibiotic-induced microbiome disruption induces diminished ovarian reserve and oocyte quality in the offspring.
- Maternal microbiome disruption decreases bile acid levels in the maternal serum.
- Supplementation with key bile acid metabolites or L. gasseri colonization rescues ovarian defects in mouse offspring.

## Abstract (web summary)

The maternal microbiome has a critical role in regulating ovarian reserve and oocyte quality in offspring in mice. Maternal antibiotic-induced microbiome disruption leads to diminished ovarian reserve in the offspring, while fecal microbiota transplantation (FMT) and supplementation with bile acid metabolites alleviates this effect.

## Graphical abstract

**Figure:**
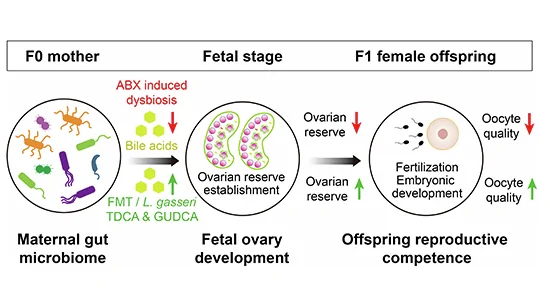

## Introduction

Diminished ovarian reserve (DOR) in women is characterized by a smaller pool of ovarian follicles in the developing ovary and is a major cause of decreased fertility, including premature ovarian insufficiency (Man et al, 2022; Rasool and Shah, 2017). Different from males, women are born with a definite pool of ovarian follicles, or ovarian reserve, which is determined during the embryonic stage (Kerr et al, 2013; Wilkosz et al, 2014), and research repeatedly demonstrates that in-utero germ cell development is vulnerable to maternal cues (Dupont et al, 2012; Perera and Herbstman, 2011). Moreover, exhaustion of the ovarian reserve is causally linked with ovarian aging (Park et al, 2021); it is therefore important to understand how maternal factors affect the ovarian reserve establishment in offspring and its subsequent importance to reproductive health.

In recent decades, the maternal gut microbiome (hereafter referred to as “maternal microbiome”) has been shown to play a critical role in mediating in-utero embryo development, including immunity (Sanidad et al, 2022), brain circuitry (Morel et al, 2023; Vuong et al, 2020), renal function (Hsu et al, 2022), metabolic phenotypes (Kimura et al, 2020), and of particular notice, the maternal microbiome alteration has been demonstrated to induce long-lasting effects during adulthood of the offspring (Di Gesu et al, 2022; Gao et al, 2021; Kimura et al, 2020; Nyangahu et al, 2018; Sun et al, 2023). Using an antibiotic-treated mouse, a recent work by Vuong et al revealed that the maternal microbiome modulates fetal neurodevelopment in mice through microbially derived metabolites and induces long-lasting effects on the adult offspring (Vuong et al, 2020). This is of particular concern since approximately 40% of pregnant women are exposed to antibiotics, and antibiotic exposure is known to induce maternal dysbiosis in humans (Miyoshi and Hisamatsu, 2022; Tai et al, 2024). Together with the fact that gut microbiota mediates ovarian function through the “gut microbiota-ovary axis” (Huang et al, 2024; Qi et al, 2019), it is therefore of significance to investigate whether the maternal microbiome causes adverse effects on ovarian function in offspring.

In this study, by applying an antibiotic-induced microbiome disruption, we show that maternal antibiotic-induced microbiome disruption leads to DOR in offspring. Specifically, using metabolomic analysis and *L. gasseri* colonization analysis, we demonstrate that the maternal microbiome may mediate in-utero ovarian development in offspring through microbially mediated bile acid metabolism in the maternal serum. Moreover, we verified that the maternal microbiome affected oocyte maturation and early embryonic development in offspring. Together, our work here emphasizes the critical role of the maternal microbiome on ovarian reserve establishment, and oocyte quality in offspring.

## Results

### Disruption of the maternal microbiome induces DOR in offspring

**Figure 1:**
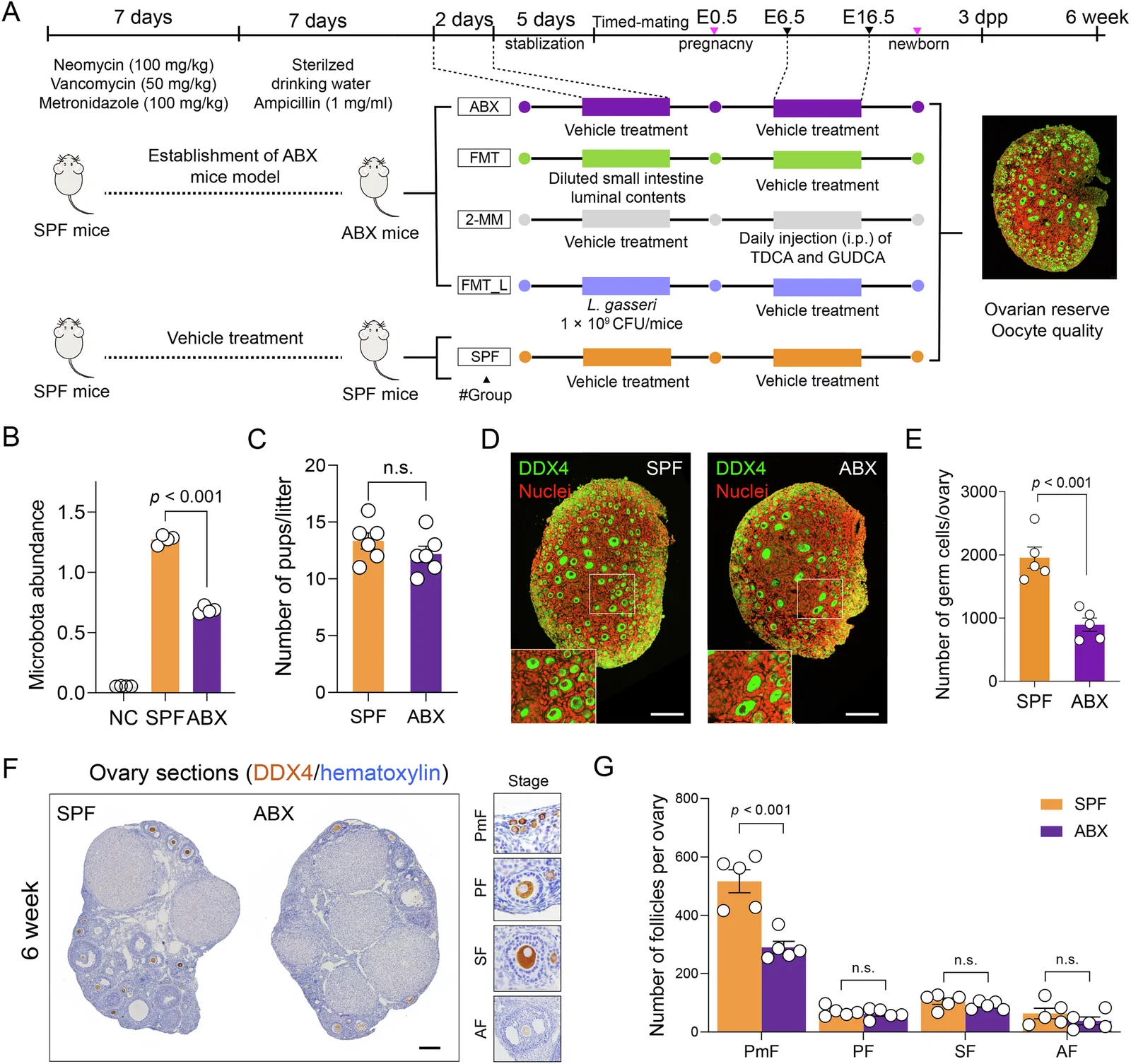
Maternal microbiome involvement in offspring ovarian reserve establishment. (**A**) Brief timeline diagram of the experimental procedure. (**B**) Relative bacterial biomass abundance of the intestinal contents from different groups (*n* = 4) after 12 h of in vitro culture. Data were quantified using a spectrophotometer at 595 nm. (**C**) Comparison of the number of offspring born from SPF (*n* = 6) and ABX (*n* = 6) dams. (**D**) DDX4 (green) staining in P3 ovaries of offspring from SPF and ABX dams. PI (red) was used to stain nuclei. Scale bars: 100 μm. (**E**) Comparison of the total number of germ cells in ovaries from SPF (*n* = 5) and ABX (*n* = 5) dams at P3. (**F**) Immunohistochemical staining of DDX4 in the ovaries of 6-week-old offspring from SPF and ABX dams. Representative images of ovarian follicles at different stages of development are displayed on the right panel. Scale bars: 200 μm. PmF primordial follicle, PF primary follicle, SF secondary follicle, AF antral follicle. (**G**) Quantification of the number of ovarian follicles at different stages of development in the ovaries of 6-week-old offspring from SPF (*n* = 5) and ABX (*n* = 5) dams. Data points in (**B**, **C**) represent individual dams and data points in (**E**, **G**) represent offspring from different dams. Data were presented as mean ± SEM. Statistical significance was determined by one-way ANOVA followed by LSD multiple comparison test (**B**) and two-tailed unpaired *t*-test (**C**,** E**,** G**). Source data are available online for this figure.

To evaluate whether the maternal microbiome affected offspring fertility, we systematically investigated how the maternal microbiome regulates ovarian reserve in offspring by performing fecal microbiota transplantation of a specific bacterial component (*L. gasseri* transplantation, hereafter termed as FMT_L group) and supplementation of two identified metabolites (Hereafter termed as 2-MM group) using an antibiotic-induced microbiome disruption (ABX) mouse model (Vuong et al, 2020). The overall experimental design is summarized in Fig. 1A.

**Figure EV1:**
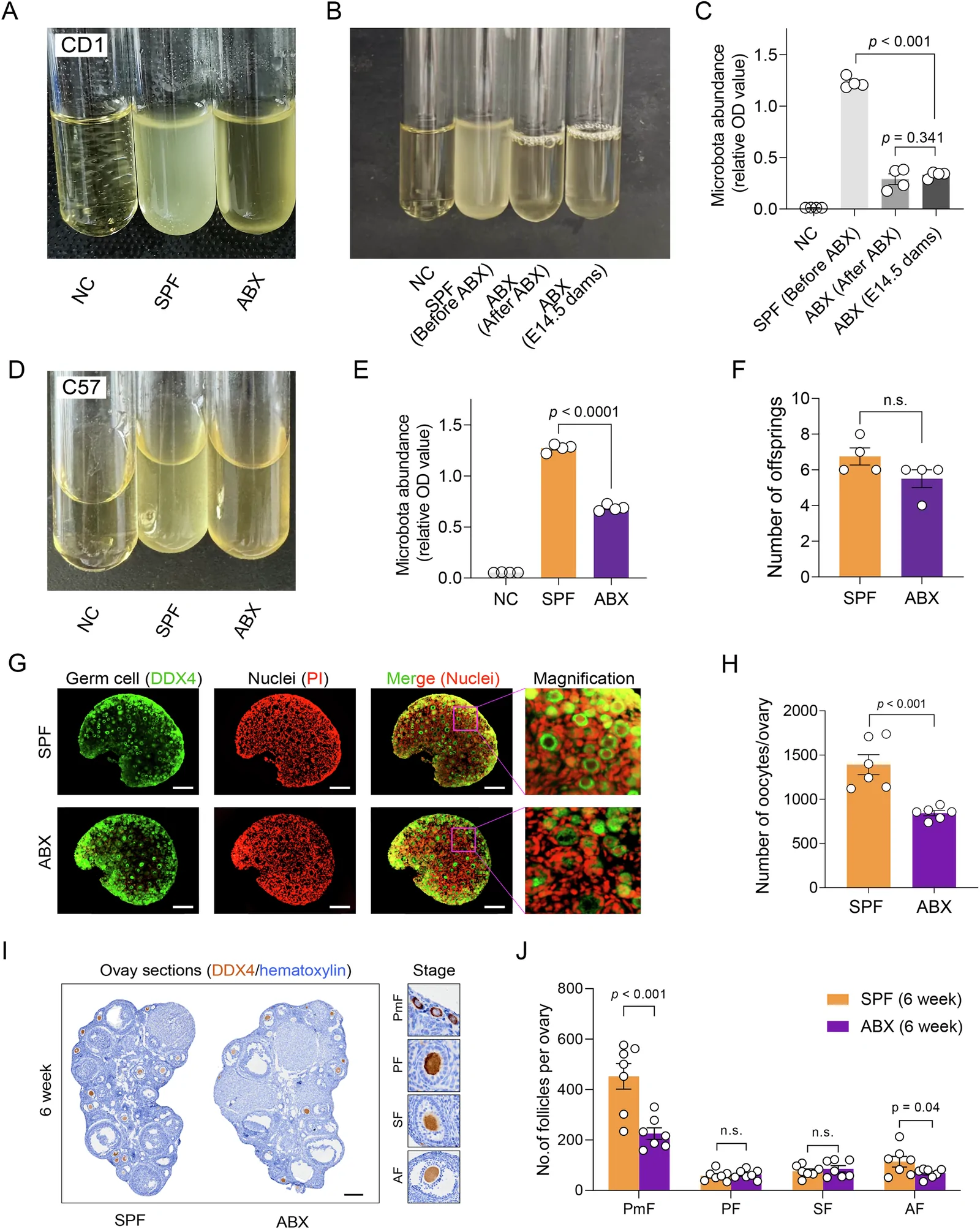
Validation of the ABX mouse model and comparison of ovarian reserve in offspring mice between SPF and ABX groups. (**A**) Representative images of bacterial cultures from the small intestine luminal contents of different groups of CD1 mice after antibiotic treatment. Images were captured after 12 h of in vitro culture. (**B**) Representative images of bacterial cultures from small intestine luminal contents in ABX mice before antibiotic administration (*n* = 4), after antibiotic administration (*n* = 4), and at E14.5 during gestation (*n* = 4). NC (*n* = 4) indicates the negative control. Images were captured after 12 h of in vitro culture. (**C**) Relative bacterial biomass abundance of intestinal contents from SPF (Before ABX), ABX (After ABX) and ABX (E14.5 dams) groups following 12 h of in vitro culture. Data were quantified using a spectrophotometer at 595 nm. (**D**) Representative images of bacterial cultures from the small intestine luminal contents of different groups of C57BL/6 mice after antibiotic treatment. Images were captured after 12 h of in vitro culture. (**E**) Relative bacterial biomass abundance of intestinal contents from different groups in C57BL/6 mice (*n* = 4) after 12 h of in vitro culture. Data were quantified using a spectrophotometer at 595 nm. (**F**) Comparison of the number of offspring in SPF (*n* = 4) and ABX (*n* = 4) dams of C57BL/6 mice. (**G**) DDX4 (green) staining in P3 ovaries of offspring from SPF and ABX dams in C57BL/6 mice. PI (red) was used to stain nuclei. Scale bars: 100 μm. (**H**) Comparison of the total number of germ cells per ovary in P3 ovaries from SPF (*n* = 6) and ABX (*n* = 6) dams of C57BL/6 mice. (**I**) Immunohistochemical staining of DDX4 in the ovaries of 6-week-old offspring from SPF and ABX dams of C57BL/6 mice. Representative images of ovarian follicles at different stages of development are displayed on the right panel. Scale bars: 200 μm. PmF primordial follicle, PF primary follicle, SF secondary follicle, AF antral follicle. (**J**) Quantification of the number of ovarian follicles at different stages of development in ovaries of 6-week-old offspring from SPF (*n* = 7) and ABX (*n* = 7) dams of C57BL/6 mice. Statistical significance was determined by one-way ANOVA followed by LSD multiple comparison test (**C**,** E**) and two-tailed unpaired *t*-test (**F**,** H**,** J**).

Firstly, 6-week-old specific pathogen-free (SPF) CD1 mice were first treated with a cocktail of antibiotics (neomycin, metronidazole, and vancomycin) for a week by gavage (Hereafter termed as ABX group), while the control group (Hereafter termed as SPF group) received an equal volume of vehicle treatment. Subsequently, the ABX mice were fed with sterile chow and water containing 1 mg/mL ampicillin for another week. Following a stabilization period with sterile chow and drinking water, mice were subjected to timed mating with wild-type males to establish pregnancy. To determine the robustness of the antibiotic-induced disruption of the maternal microbiome after antibiotic administration, we analyzed the bacterial biomass density of microbiota using intestinal contents isolated from SPF and ABX mice, and after a 12-h in vitro culture, the results confirmed that antibiotic exposure significantly decreased bacterial biomass density in the ABX group (Figs. 1B and EV1A; *p* < 0.001). In addition, by comparing the intestinal bacterial biomass density in ABX mice before antibiotic administration, after antibiotic administration, and at embryonic day 14.5 (E14.5), we confirmed that antibiotic-induced maternal microbiome disruption could persist until E14.5 (Fig. EV1B,C; *p* < 0.001), further verifying the effectiveness of antibiotic-induced maternal microbiome disruption during pregnancy.

To evaluate whether disruption of the maternal microbiome directly affected pregnancy outcomes, we compared the number of pups between SPF and ABX dams; the results showed that the number of pups in the ABX group tended to decrease compared with the SPF group, but the results were not significant (Fig. 1C). Next, to evaluate whether the maternal microbiome affected the ovarian reserve establishment in offspring, we compared the number of germ cells per ovary using immunofluorescence analysis of germ cell marker DDX4 (Ge et al, 2021) on ovaries collected at postnatal day 3 (P3) from offspring of SPF and ABX dams (Fig. 1D). Compared with ovaries in the offspring of SPF dams, it was observed that ovaries in the offspring of ABX dams showed a decreased number of DDX4-positive germ cells per ovary (Fig. 1E; *p* < 0.001), indicating a DOR phenotype after antibiotic-induced disruption of the maternal microbiome.

We next reasoned whether antibiotic-mediated disruption of the maternal microbiome caused long-lasting effects during offspring adulthood. To this end, we sectioned 6-week-old ovaries from offspring of SPF and ABX dams (Fig. 1F). Morphologically, we observed that ovaries from offspring of ABX dams showed a decreased number of DDX4-positive germ cells. To investigate whether antibiotic-mediated disruption of the maternal microbiome affected folliculogenesis, we analyzed the number of primordial (PmF), primary (PF), secondary (SF), and antral (AF) follicles between ovaries from offspring of SPF and ABX dams. Results showed that the number of PmF stage follicles in the ovaries from offspring of ABX dams significantly decreased when compared with the offspring of SPF dams (Fig. 1G; *p* < 0.001), which was consistent with the scenario we observed at P3. Notably, no significant differences were observed in the growing-stage ovarian follicles. Together, these results indicated that disruption of the maternal microbiome mainly compromised ovarian reserve establishment in offspring, whereas folliculogenesis was largely unaffected.

Furthermore, we validated our findings using the C57BL/6 mouse strain. The analysis of bacterial biomass density indicated that antibiotic treatment effectively disrupted the gut microbiome abundance in C57BL/6 mice (Fig. EV1D,E). Analyzing the number of pups between SPF and ABX dams in C57BL/6 mice revealed a decrease in the number of pups in ABX dams (Fig. EV1F), but it was not statistically significant, consistent with the scenario observed in CD1 mice. Importantly, we found that disruption of the maternal microbiome in C57BL/6 dams decreased the number of germ cells per ovary at P3 and reduced the PmF stage follicles in adult offspring of ABX dams (Fig. EV1G–J), which was consistent with our observations in the CD1 mice. Together, these results revealed that disruption of the maternal microbiome induced DOR in offspring in both CD1 and C57BL/6 mice.

### Disruption of the maternal microbiome increases germ cell DNA damage in fetal ovaries

**Figure 2:**
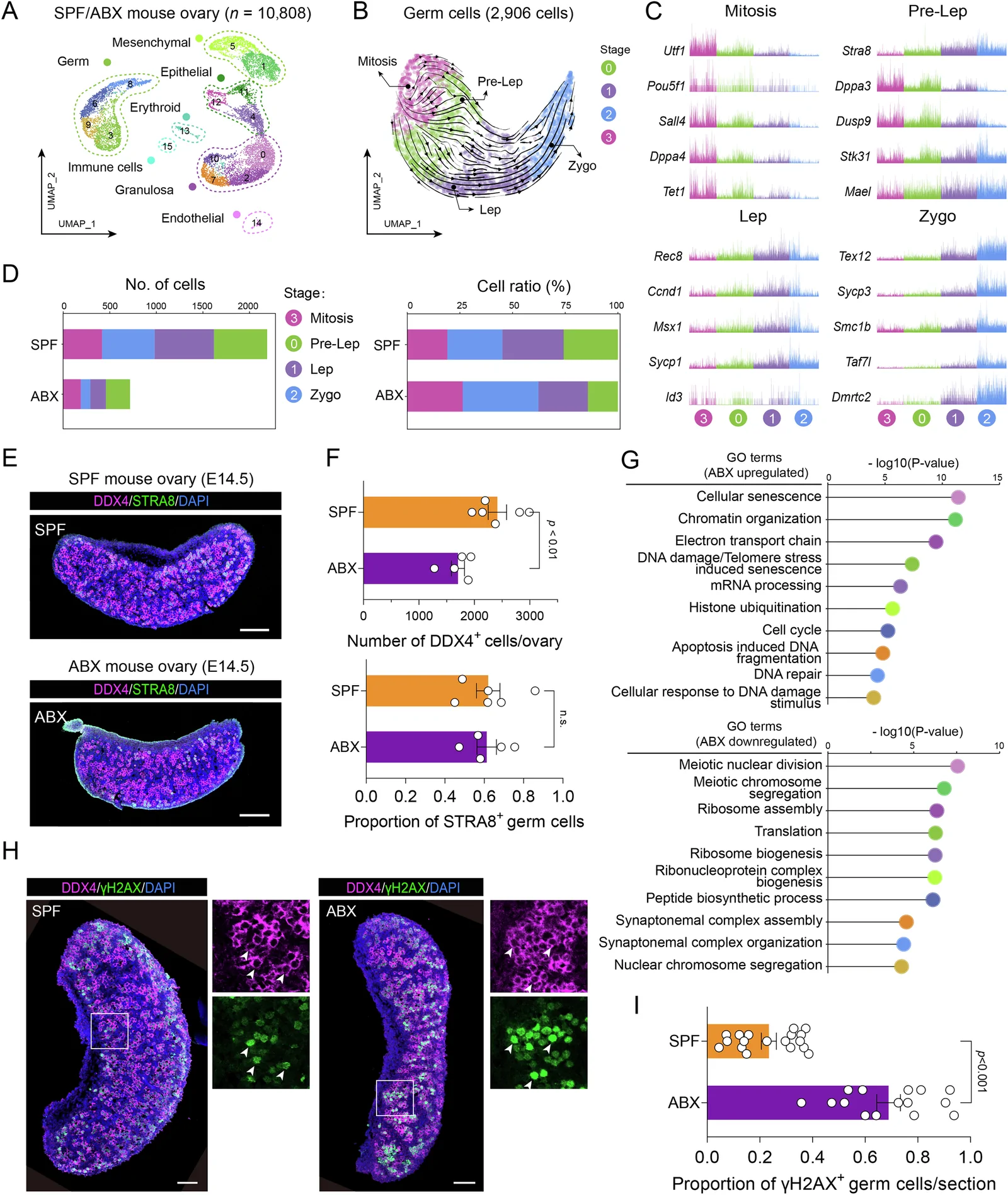
Understanding the effect of the maternal microbiome on early germ cell development using scRNA seq. (**A**) Establishment of a scRNA seq atlas using E14.5 ovaries from SPF and ABX dams. The ovaries from each group were collected from at least eight independent fetuses. (**B**) Fine-scale analysis of germ cell clusters. RNA velocity vectors were projected to the UMAP plot with the arrows indicating the predicted future state of their respective cells. Pre-Lep pre-leptotene, Lep leptotene, Zygo zygotene. (**C**) Trackplot illustrating representative germ cell marker gene expression at different stages of MPI. (**D**) Comparison of the number of germ cells and proportion of germ cells in E14.5 ovaries from SPF and ABX dams based on scRNA seq profiles. (**E**) Immunofluorescence staining of DDX4 (magenta) and STRA8 (green) expression in E14.5 fetal ovaries from SPF and ABX dams. Scale bars: 100 μm. (**F**) Comparison of the number of DDX4-positive germ cells per ovary and the proportion of STRA8 and DDX4 double-positive germ cells in E14.5 ovaries from SPF (*n* = 6) and ABX dams (*n* = 5) based on immunofluorescence analysis. (**G**) Lollipop chart illustrating the GO enrichment analysis of differentially expressed genes after ABX treatment. The top panel shows the results of ABX up-regulated GO terms, and the bottom panel shows the results of ABX downregulated GO terms. (**H**) Immunofluorescence staining of DDX4 (magenta) and γH2AX (green) expression in E14.5 fetal ovaries from SPF and ABX dams. Scale bars: 50 μm. (**I**) Comparison of the proportion of γH2AX-positive germ cells per section in E14.5 ovaries from SPF (*n* = 18) and ABX (*n* = 15) dams based on immunofluorescence analysis. Data points in (**F**) represent offspring from different dams, and data points in (**I**) represent individual ovarian sections. Data were presented as mean ± SEM. Statistical significance was determined by a two-tailed unpaired *t*-test (**F**,** I**). Source data are available online for this figure.

**Figure EV2:**
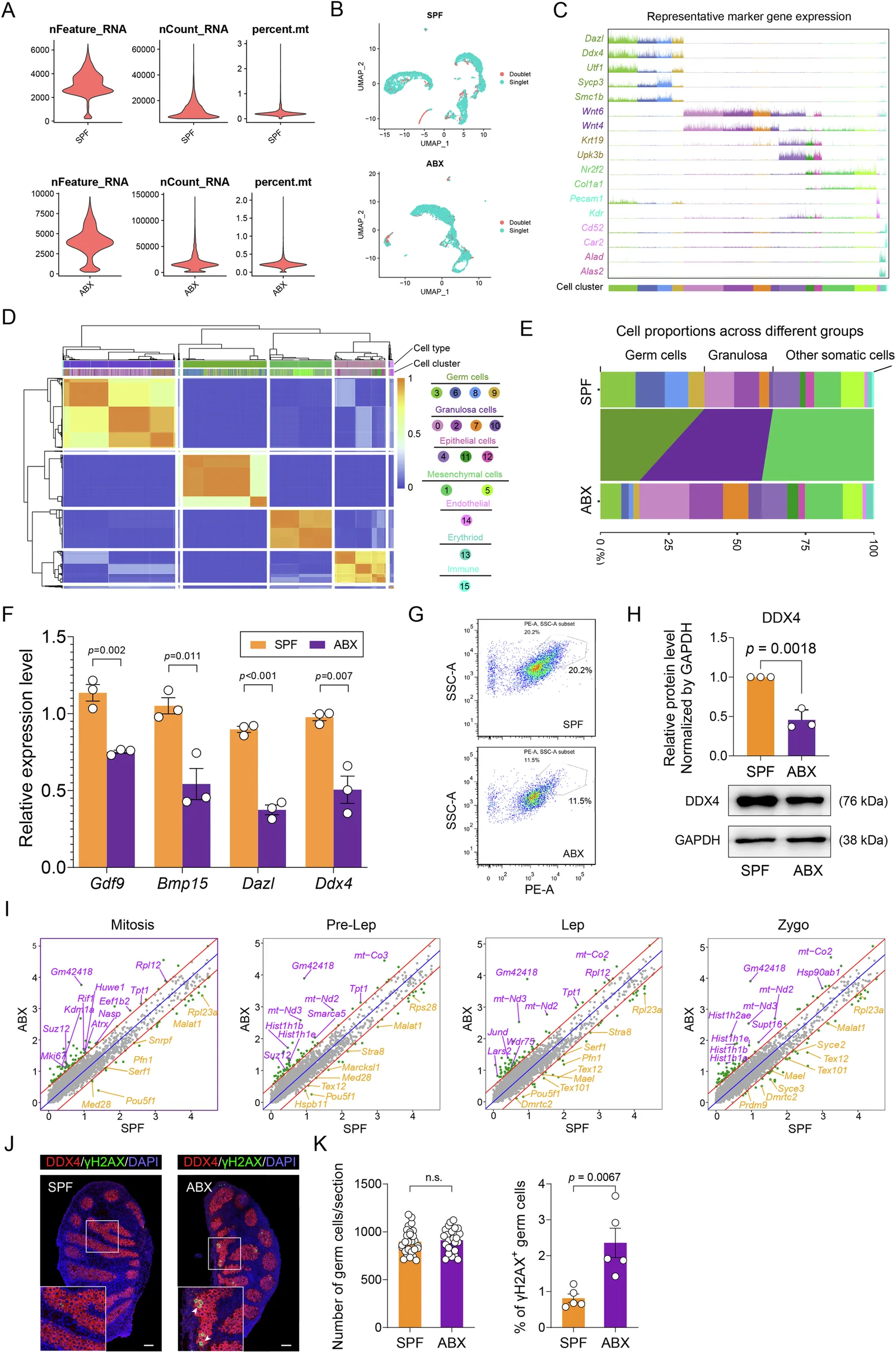
Quality control of scRNA seq datasets and validation of DDX4 expression in offspring ovaries from SPF and ABX dams. (**A**) Comparison of the number of genes detected in each cell (nFeature_RNA), number of UMIs per cell (nCount_RNA), and the percentage of mitochondrial genes (percent.mt) of scRNA seq data from the SPF and ABX groups. (**B**) UMAP plot indicates potential doublets and singlets of scRNA seq data from the SPF and ABX groups. Doublet indicates two cells encapsulated into one reaction volume, while a singlet indicates one cell encapsulated. (**C**) Trackplot indicating representative cell marker gene expression in different cell clusters. (**D**) Consensus clustering of single-cell RNA-seq data from the SPF and ABX groups. (**E**) Comparison of cell cluster proportions between the SPF and ABX groups based on scRNA seq. (**F**) RT-qPCR comparison of representative germ cell marker gene expression between SPF (*n* = 3) and ABX (*n* = 3) fetal ovaries at E14.5. *Gapdh* was used as a loading control. Data were presented as mean ± SEM. (**G**) Flow cytometry quantification of the percentage of DDX4⁺ germ cells between SPF and ABX fetal ovaries at E14.5. For each group, ovaries were collected from at least five independent fetuses. (**H**) Western blotting of germ cell marker DDX4 expression between the SPF (*n* = 3) and ABX (*n* = 3) groups. The expression level of DDX4 was normalized by GAPDH. (**I**) Comparative analysis and identifying germ cell-specific differentially expressed genes at various stages of meiosis between the SPF and ABX groups based on scRNA seq. (**J**) Representative immunofluorescence images showing DDX4 (red) and γH2AX (green) expression in E14.5 fetal testes from SPF and ABX dams. Scale bars, 50 μm. (**K**) Comparison of the total number of germ cells per section (left panel, *n* = 27, 26 for SPF and ABX, respectively) and the proportion of γH2AX-positive germ cells (right panel, *n* = 5, 5 for SPF and ABX, respectively) in E14.5 testes from SPF and ABX dams. Data were presented as mean ± SEM. Statistical significance was determined by a two-tailed unpaired *t*-test.

To investigate how the maternal microbiome mediated ovarian reserve establishment, we generated a single-cell gene expression profile of E14.5 fetal ovaries from ABX dams using the C57BL/6 mouse strain and compared them to our previously published single-cell gene expression profile of E14.5 SPF mouse ovaries (Ge et al, 2021). After stringent quality control, we generated a total of 10,808 single-cell gene expression profiles, and these cells were then projected to uniform manifold approximation and projection (UMAP) analysis (Figs. 2A and EV2A,B). By evaluating cluster-specific marker gene expression, we characterized seven major cell types, including germ cells (*Ddx4* and *Dazl*), granulosa cells (*Wnt6*, *Wnt4*), mesenchymal cells (*Col1a1* and *Nr2f2*), epithelial cells (*Krt19* and *Upk3b*), endothelial cells (*Pecam1* and *Kdr*), immune cells (*Cd52* and *Car2*), and erythroid cells (*Alad* and *Alas2*) (Fig. EV2C) (Ge et al, 2021). Consensus clustering of single-cell RNA-seq data revealed that cell clusters from the same type always clustered in the same module, further confirming the accuracy of our cell type characterization (Fig. EV2D) (Kiselev et al, 2017).

Based on the ovarian single-cell gene expression map from the fetuses of SPF and ABX dams, we next compared major cell proportions between SPF and ABX groups, where the percentage of germ cells from the ABX group decreased by more than twofold when compared with the SPF group (Fig. EV2E). To investigate whether the maternal microbiome disruption-induced DOR was causally associated with compromised early germ cell development, we performed a fine-scale analysis of the germ cell clusters (Fig. 2B). Re-analysis of germ cell clusters using UMAP identified four major cell clusters, and characterization of gene expression profiles revealed that these four clusters corresponded to germ cells at mitosis, pre-leptotene, leptotene, and zygotene stages of meiotic prophase I (Fig. 2C). The embedding of RNA velocity also confirmed the developmental trajectory from mitosis to the zygotene stage (La Manno et al, 2018). We then compared the progression of the meiotic program between ovaries from SPF and ABX fetuses; the results showed that germ cells at different stages of meiotic prophase I in the ABX group were comparable to those of the SPF group, while the total number of germ cells in the ABX group was decreased by more than twofold (Fig. 2D).

Consistent with our findings generated from single-cell transcriptome, gene expression analysis using quantitative reverse transcription PCR (RT-qPCR) further confirmed that the expression level of germ cell-specific markers, including *Gdf9*, *Bmp15*, *Dazl*, and *Ddx4*, was significantly decreased in the E14.5 ovary of ABX dams (Fig. EV2F). At the protein level, both flow cytometry and western blotting quantification of DDX4⁺ germ cells unveiled a reduction in the proportion of germ cells in the E14.5 ovary of ABX dams compared to SPF controls (Fig. EV2G,H). Similarly, immunofluorescence analysis unveiled that the total number of germ cells per ovary was significantly decreased in the E14.5 ovaries from fetuses of ABX dams in CD1 mice (Fig. 2E,F, top panel, *p* < 0.01). Moreover, analyzing the percentage of STRA8 expression in germ cells, a well-characterized marker of meiotic germ cells (Anderson et al, 2008), showed no difference in E14.5 ovaries from the fetuses of SPF and ABX dams (Fig. 2F, bottom panel), further supporting our single-cell analysis. Together, these findings consistently indicate that disruption of the maternal microbiome leads to germ cell loss at the early stage of germ cell development, but does not impact the progression of the meiotic program, in both CD1 and C57BL/6 mice.

To gain further insight into how the disruption of the maternal microbiome induced germ cell loss at the early stage of germ cell development, we performed differential gene expression analysis using germ cells from fetuses of SPF and ABX dams. Notably, it was observed that antibiotic-mediated disruption of the maternal microbiome significantly perturbed gene expression profiles of fetal germ cells en route to meiosis (Fig. EV2I). GO enrichment analysis of all ABX perturbed genes revealed that ABX up-regulated genes were enriched in the GO terms related to DNA damage and repair (Fig. 2G, top panel). For the ABX downregulated genes, these genes were mainly enriched in GO terms related to the meiotic program (Fig. 2G, bottom panel). Consistently, co-staining of DNA damage marker γH2AX (Mah et al, 2010) with germ cell marker DDX4, using E14.5 ovaries from the fetuses of SPF and ABX dams, revealed that γH2AX was co-located with DDX4 signals, indicating their expression in the germ cell populations (Fig. 2H). Importantly, analyzing the percentage of γH2AX-positive germ cells per section revealed that germ cells from the fetuses of ABX dams showed significantly higher levels of γH2AX-positive germ cells when compared with the fetuses from SPF dams (Fig. 2I, *p* < 0.001), thus indicating increased levels of DNA double-stranded breaks and unpaired meiotic chromatin in germ cells from the fetal ovaries of ABX dams (Malki et al, 2014).

In contrast, co-staining of DDX4 and γH2AX in E14.5 fetal testis revealed that the percentage of γH2AX-positive germ cells was significantly increased in the fetuses of ABX dams, while the total number of germ cells was comparable between SPF and ABX groups (Fig. EV2J,K). Notably, the magnitude of the increase in γH2AX-positive germ cells following maternal microbiome disruption was substantially lower in fetal testes than in fetal ovaries (~2.5 vs ~60%). These findings therefore suggest that maternal microbiome disruption may exert a sex-biased effect on fetal germ cell development. Combined with previous findings that germ cells with increased levels of meiotic defects are eliminated during early oogenesis, it is therefore plausible that maternal microbiome disruption-induced DOR may be causally related to the increased meiotic defects during early germ cell development.

### FMT alleviates maternal microbiome disruption-induced DOR in F1 offspring

**Figure EV3:**
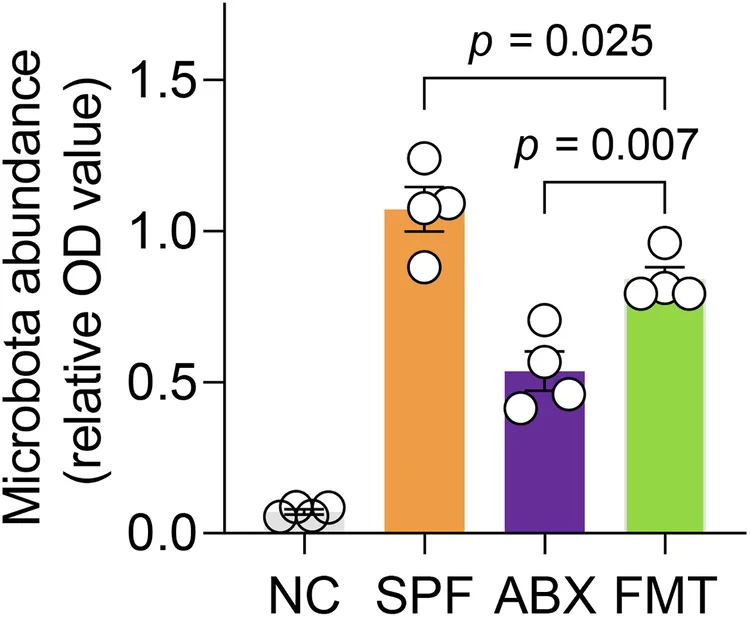
Validation of FMT on rescuing intestinal microbiota abundance in CD1 mice. Intestinal microbiota abundance in different groups analyzed by a spectrometer at 595 nm. Data were presented as mean ± SEM. Statistical significance was determined by one-way ANOVA followed by the LSD multiple comparison test.

To further verify whether the DOR phenotypes in the offspring of ABX dams were causally associated with the disrupted maternal microbiome, we performed an FMT assay in ABX mice (hereafter termed the FMT group). To this end, we transplanted ABX mice with small intestine luminal contents from aged-matched SPF mice using methods previously described (Fig. 1A) (Zhang et al, 2020). After FMT, bacterial biomass density analysis unveiled significantly elevated microbiota abundance in the FMT group when compared with the ABX group (Fig. EV3, *p* = 0.007), thus indicating the effectiveness of FMT in alleviating antibiotic-induced maternal microbiome disruption.

**Figure 3:**
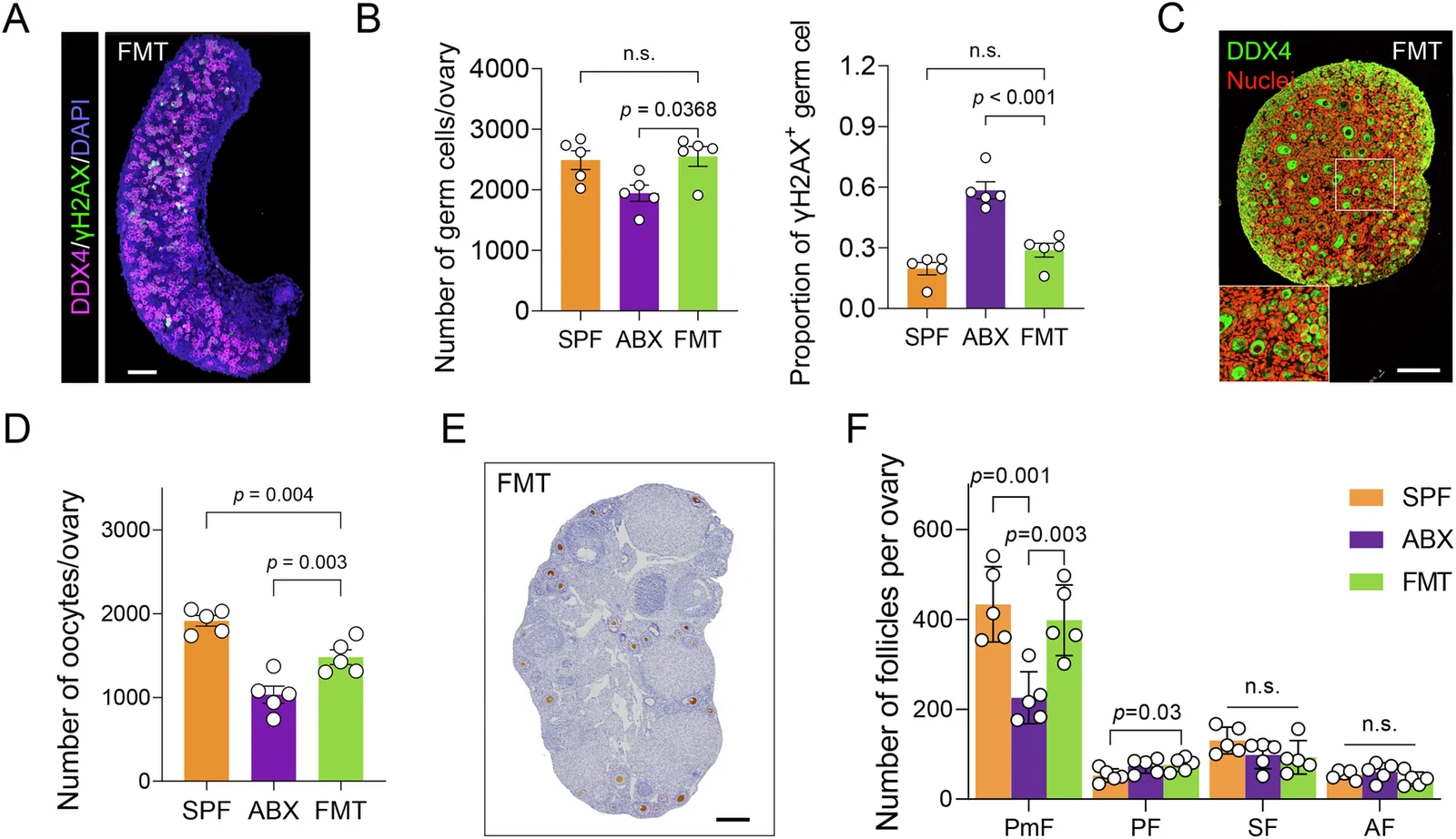
Prevention of maternal microbiome disruption-induced DOR in offspring by FMT. (**A**) Immunofluorescence staining of DDX4 (magenta) and γH2AX (green) expression in E14.5 fetal ovaries from FMT dams. Scale bars: 50 μm. (**B**) Comparison of the total number of germ cells per ovary and the proportion of γH2AX-positive germ cells in E14.5 ovaries from SPF (*n* = 5), ABX (*n* = 5), and FMT (*n* = 5) dams based on immunofluorescence analysis. (**C**) DDX4 (green) staining in P3 ovaries of offspring from FMT dams. PI (red) was used to stain nuclei. Scale bars: 100 μm. (**D**) Quantification of the total number of germ cells per ovary in P3 ovaries from SPF (*n* = 5), ABX (*n* = 5), and FMT (*n* = 5) dams. (**E**) Representative immunohistochemical staining image of DDX4 in the ovaries of 6-week-old offspring from the FMT dam. Scale bars: 200 μm. (**F**) Quantification of the number of ovarian follicles at different stages of development in the ovaries of 6-week-old offspring from SPF (*n* = 5), ABX (*n* = 5), and FMT (*n* = 5) dams. Data points in (**B**, **D**, **F**) represent offspring from different dams. Data were presented as mean ± SEM. Statistical significance was determined by one-way ANOVA followed by LSD multiple comparison test (**B**, **D**) and two-way ANOVA followed by Tukey’s multiple comparisons test (**F**). Source data are available online for this figure.

Using FMT-treated mice, we next reasoned whether FMT could rescue maternal microbiome disruption-induced DOR in offspring. Immunofluorescence analysis unveiled that the total number of germ cells in the E14.5 fetal ovaries from FMT dams was significantly elevated and was similar to the SPF group (Fig. 3A,B, left panel). Further comparing the percentage of γH2AX-positive germ cells in the fetuses of FMT dams with the fetuses of ABX dams unveiled that the percentage of γH2AX-positive germ cells significantly decreased after FMT (Fig. 3B, right panel; *p* < 0.001), and the percentage of γH2AX-positive germ cells in the FMT group was close to that of the SPF group.

Next, we counted the total number of oocytes at P3, and the results showed that FMT alleviated maternal microbiome disruption-induced germ cell loss in offspring (Fig. 3C,D). Furthermore, we further sectioned the ovaries of 6-week-old offspring of FMT dams (Fig. 3E), and compared the total number of ovarian follicles with those of ABX dams; the results revealed that FMT alleviated maternal microbiome disruption-induced DOR, and significantly increased the number of PmF in the offspring of FMT dams (Fig. 3F; *p* = 0.003). Together, these results suggested that FMT before conception in ABX dams alleviated maternal microbiome disruption-induced DOR in offspring.

### Key metabolite supplementation restores maternal microbiome disruption-induced DOR in F1 offspring

**Figure 4:**
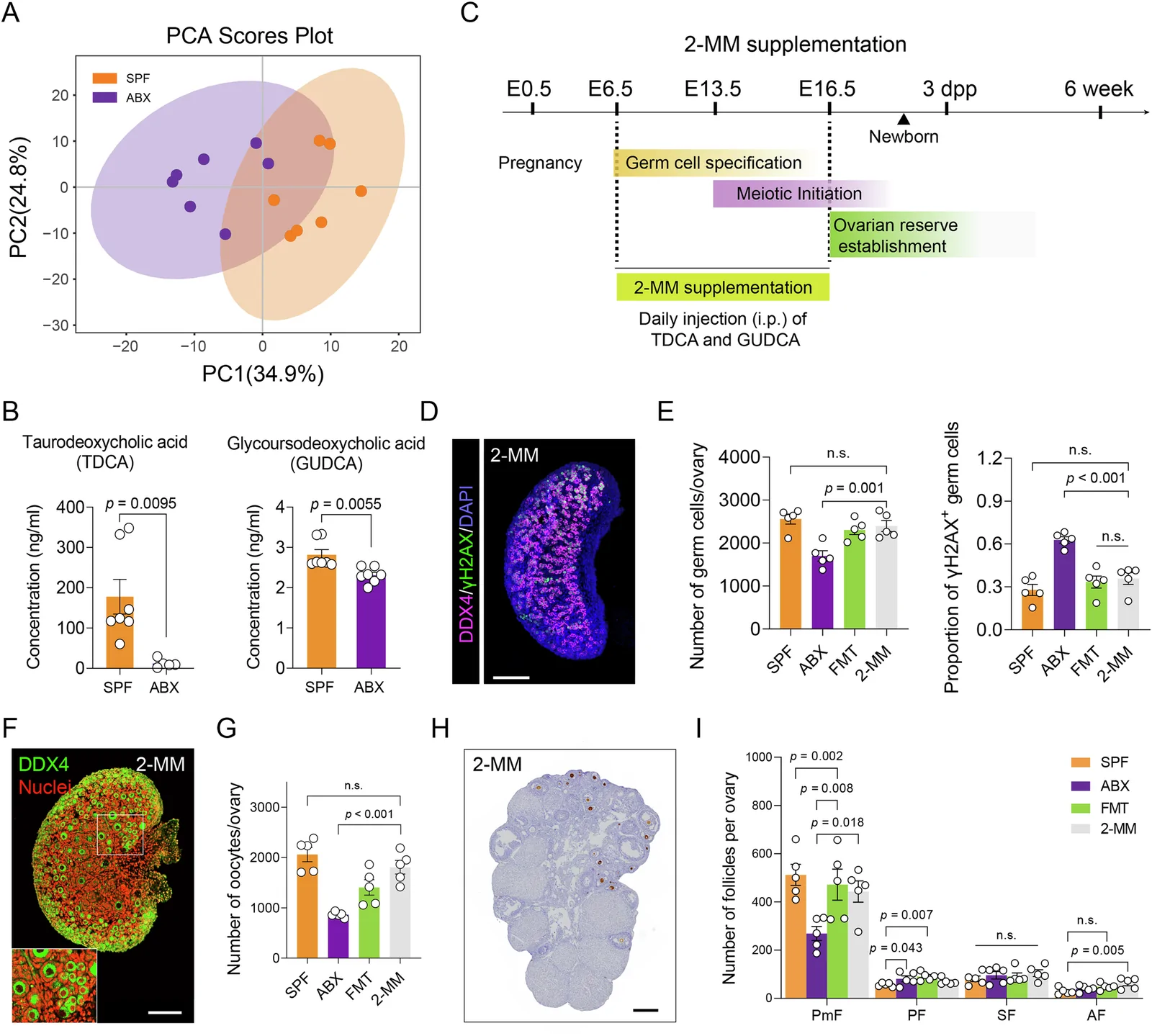
Supplementation of TDCA and GUDCA prevented maternal microbiome disruption-induced DOR in offspring. (**A**) Principal component analysis (PCA) on metabolomic profiles of maternal serum from the SPF (*n* = 7) and ABX (*n* = 7) dams at E14.5. For each group, serum samples were collected from seven different dams. (**B**) Quantification of TDCA (SPF, *n* = 7; ABX, *n* = 5, with two samples not detected) and GUDCA (SPF, *n* = 7; ABX, *n* = 7) levels in the maternal serum of SPF and ABX dams at E14.5 based on metabolomic analysis. (**C**) Brief timeline diagram illustrating selected metabolite supplementation procedure. (**D**) Immunofluorescence staining of DDX4 (magenta) and γH2AX (green) expression in E14.5 fetal ovaries from 2-MM dams. Scale bars: 100 μm. (**E**) Comparison of the total number of germ cells per ovary and the proportion of γH2AX-positive germ cells in E14.5 ovaries from SPF (*n* = 5), ABX (*n* = 5), FMT (*n* = 5), and 2-MM (*n* = 5) dams based on immunofluorescence analysis. (**F**) DDX4 (green) staining in P3 ovaries of offspring from 2-MM dams. PI (red) was used to stain nuclei. Scale bars: 100 μm. (**G**) Comparison of the total number of germ cells per ovary in P3 ovaries from SPF (*n* = 5), ABX (*n* = 5), FMT (*n* = 5), and 2-MM (*n* = 5) dams. (**H**) Representative immunohistochemical staining of DDX4 in the ovaries of 6-week-old offspring from 2-MM dams. Scale bars: 200 μm. (**I**) Comparison of the number of ovarian follicles at different stages of development in the ovaries of 6-week-old offspring from SPF (*n* = 5), ABX (*n* = 5), FMT (*n* = 5), and 2-MM (*n* = 5) dams. Data points in (**B**) represent serum samples obtained from different dams, and data points in (**E**, **G**, **I**) represent offspring from different dams. Data were presented as mean ± SEM. Statistical significance was determined by two-tailed unpaired *t*-test (**B**), one-way ANOVA followed by LSD multiple comparison test (**E**,** G**) and two-way ANOVA followed by Tukey’s multiple comparisons test (**I**). Source data are available online for this figure.

**Figure EV4:**
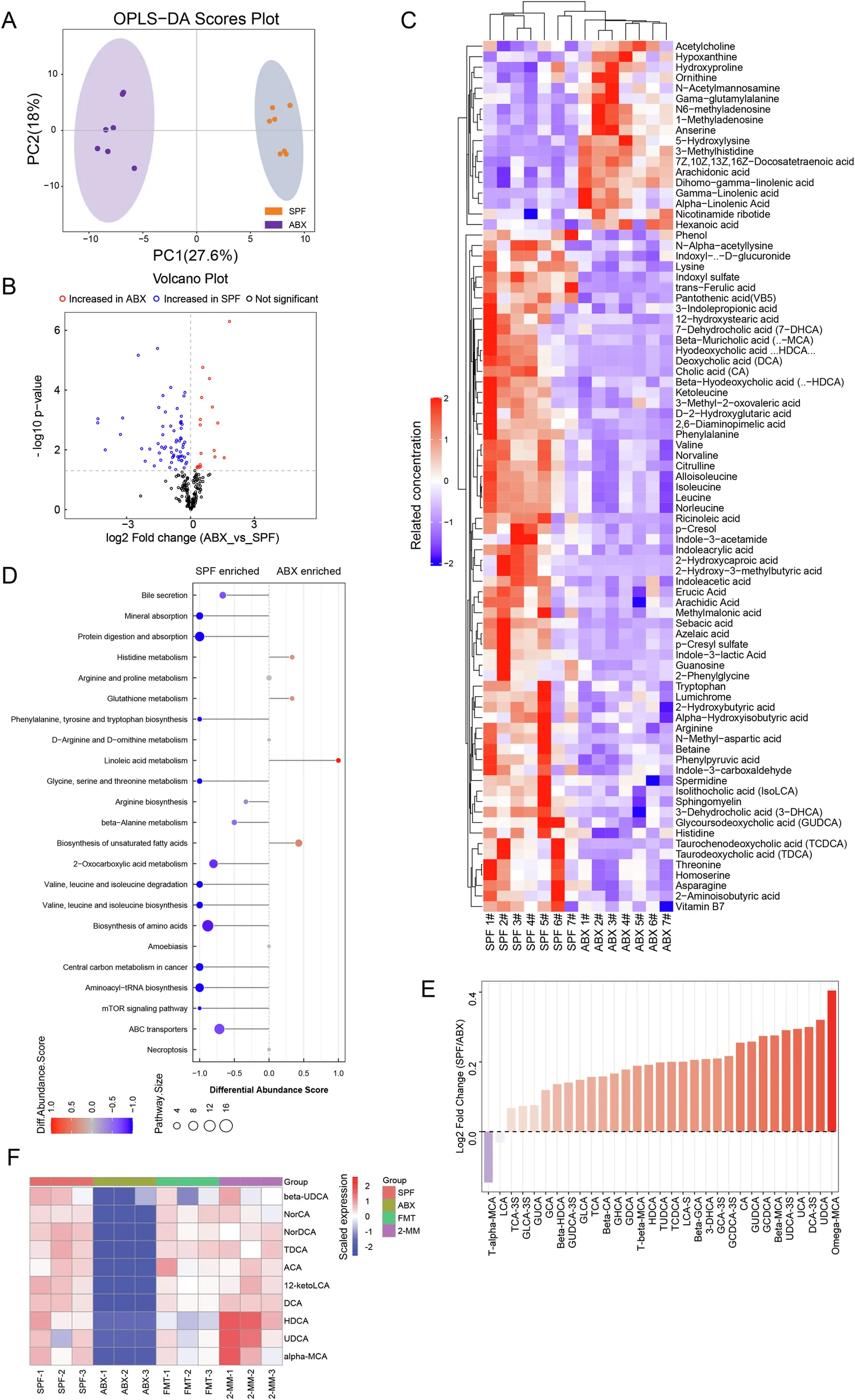
Metabolomic profiling of E14.5 maternal serum in SPF and ABX dams. (**A**) Orthogonal partial least squares discriminant analysis (OPLS-DA) on metabolomic profiles of maternal serum from SPF (*n* = 7) and ABX (*n* = 7) dams at E14.5. For each group, serum samples were collected from seven different dams. (**B**) Volcano plot illustrating differentially accumulated metabolites in maternal serum of SPF (*n* = 7) and ABX (*n* = 7) dams. (**C**) Heatmap illustrating the relative expression level of identified differentially accumulated metabolites between maternal serum from SPF and ABX dams. (**D**) Differential abundance (DA) score analysis of KEGG-enriched pathways in maternal serum from SPF and ABX dams. (**E**) Bar chart illustrating the Log2 fold change (SPF/ABX) of 31 detected bile acids in fetal ovaries from SPF and ABX dams at E14.5. For each group, at least 30 ovaries were included for analysis, and the final concentrations were normalized to cell number. (**F**) Heatmap illustrating the scaled abundance levels of ten representative bile acids in the maternal serum of SPF, ABX, FMT, and 2-MM dams. Each group includes three replicates, with each replicate representing a serum sample from a different dam. Statistical significance was determined by a two-tailed unpaired *t*-test (**B**).

The maternal microbiota has been demonstrated to affect fetal development through mediating metabolites in the maternal serum (Vuong et al, 2020). To determine which metabolites may be involved in maternal microbiome disruption-induced DOR, we performed metabolomic analysis using maternal serum from SPF and ABX dams at E14.5 of gestation. Notably, principal component analysis (PCA) and orthogonal projections to latent structures discriminant analysis (OPLS-DA) revealed that the maternal metabolites showed distinct clustering patterns (Figs. 4A and EV4A) between SPF and ABX groups, indicating significant alterations of metabolomic profiles after maternal microbiome disruption. By performing differential abundance analysis, we identified 83 differentially accumulated metabolites (Fig. EV4B; Dataset EV1), of which 65 showed higher abundance in the maternal serum of SPF dams, while 18 showed higher abundance in the maternal serum of ABX dams (Fig. EV4C). Furthermore, combined analysis of Kyoto Encyclopedia of Genes and Genomes (KEGG) enrichment and differential abundance score analysis using differentially accumulated metabolites indicated that the pathway of “Bile secretion” was under-represented in the maternal serum of ABX dams (Fig. EV4D), indicating maternal microbiome disruption induced alteration in bile acid metabolism in the maternal serum of ABX dams. To further verify whether changes in maternal serum metabolomic profiles affect those in the fetal ovary, we performed targeted metabolomics to evaluate representative bile acid levels in fetal ovaries from SPF and ABX dams. The results revealed that 29 out of the total 31 detected bile acids showed reduced abundance in the fetal ovaries of ABX dams compared to SPF dams (Fig. EV4E; Table EV1), suggesting that maternal microbiome disruption-induced alterations in bile acid metabolism in the maternal serum are reflected within the fetal ovary.

Next, we reasoned whether supplementation of key metabolites involved in bile acid metabolism rescued maternal microbiome disruption-induced DOR in F1 offspring. According to metabolomic analysis, we focused on two bile acids, taurodeoxycholic acid (TDCA) and glycoursodeoxycholic acid (GUDCA), which are significantly decreased in the maternal serum of ABX dams (Fig. 4B) and have been known to regulate bile acid metabolism (Chen et al, 2023). We then supplemented the ABX dams with TDCA and GUDCA (2-MM group) daily from E6.5 to E16.5 of gestation (Fig. 4C), the developmental timepoint during which germ cell specification starts (Saitou and Yamaji, 2012). Notably, maternal supplementation of TDCA and GUDCA rescued maternal microbiome disruption-induced DOR (Fig. 4D–I). Specifically, in the offspring of 2-MM dams, the total number of germ cells per ovary was significantly increased (Fig. 4E, left panel, *p* = 0.001), and the percentage of γH2AX-positive germ cells significantly decreased when compared with offspring from ABX dams (Fig. 4E, right panel, *p* < 0.001). Besides, we observed that the DOR phenotypes in the ABX offspring were prevented after 2-MM supplementation at P3 (Fig. 4F,G). In the adult stage, 2-MM supplementation also exhibited rescuing effects, with 2-MM supplementation significantly increasing the total number of PmF in the 6-week-old ovaries (Fig. 4H,I).

Furthermore, to verify that key metabolites involved in bile acid metabolism act to rescue maternal microbiome disruption-induced DOR in F1 offspring, we compared the relative levels of ten representative bile acids in the maternal serum of SPF, ABX, FMT, and 2-MM dams using targeted liquid chromatography-mass spectrometry (Fig. EV4F; Table EV2). The results showed that these bile acids had the lowest expression levels in the maternal serum of ABX dams, while their serum levels were elevated in the FMT and 2-MM dams. Together, these data suggest that microbially mediated bile acid metabolism in the maternal serum plays an indispensable role in mediating ovarian reserve establishment in offspring.

### *L. gasseri* colonization rescues maternal microbiome disruption-induced DOR in F1 offspring

**Figure EV5:**
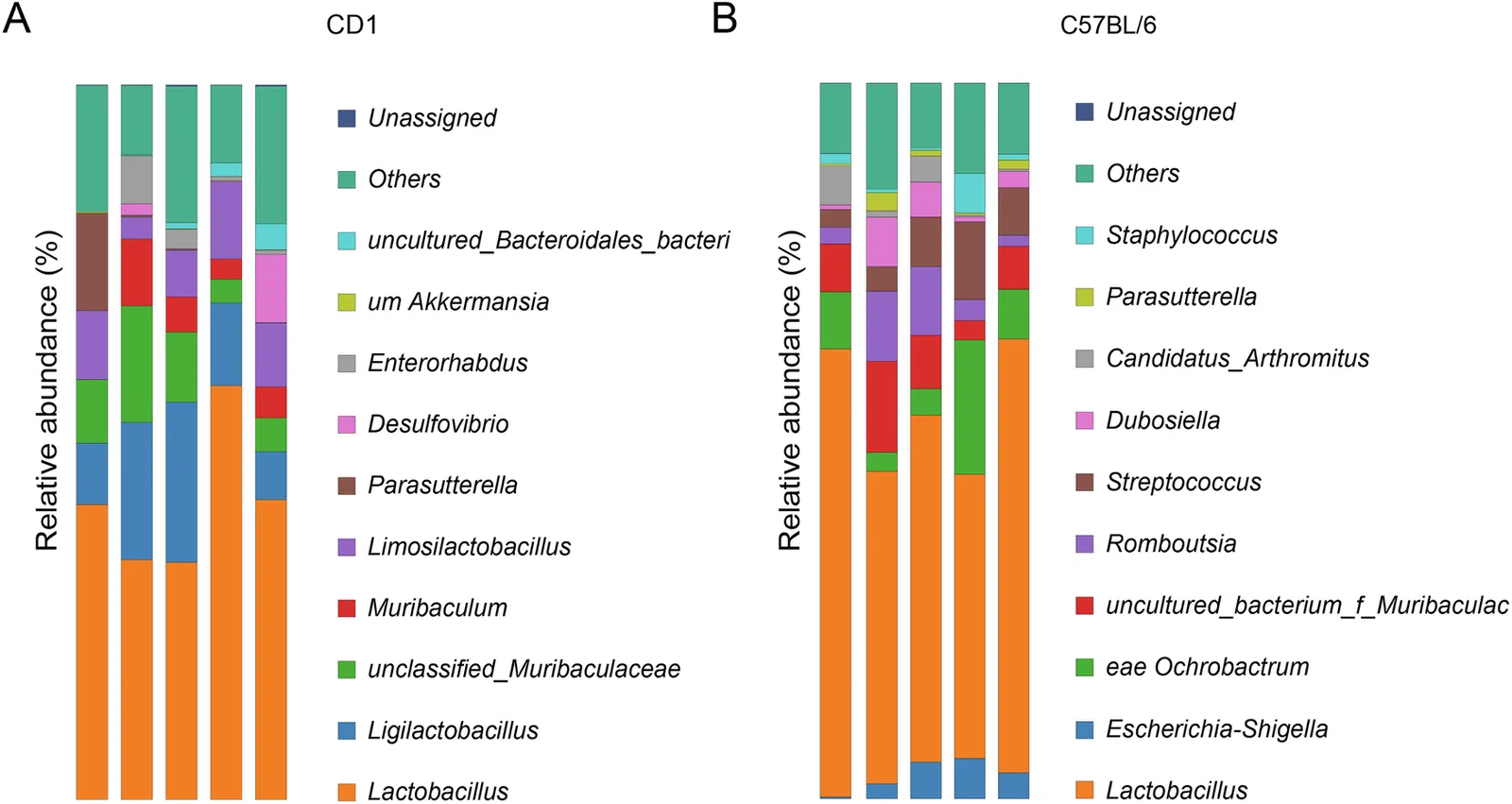
Comparison of microbiota conservation between CD1 and C57 mice. (**A**) Genus-level taxonomic diversity of maternal fecal microbiota collected from SPF mice of the CD1 strain. (**B**) Genus-level taxonomic diversity of maternal fecal microbiota collected from SPF mice of the C57BL/6 strain.

**Figure 5:**
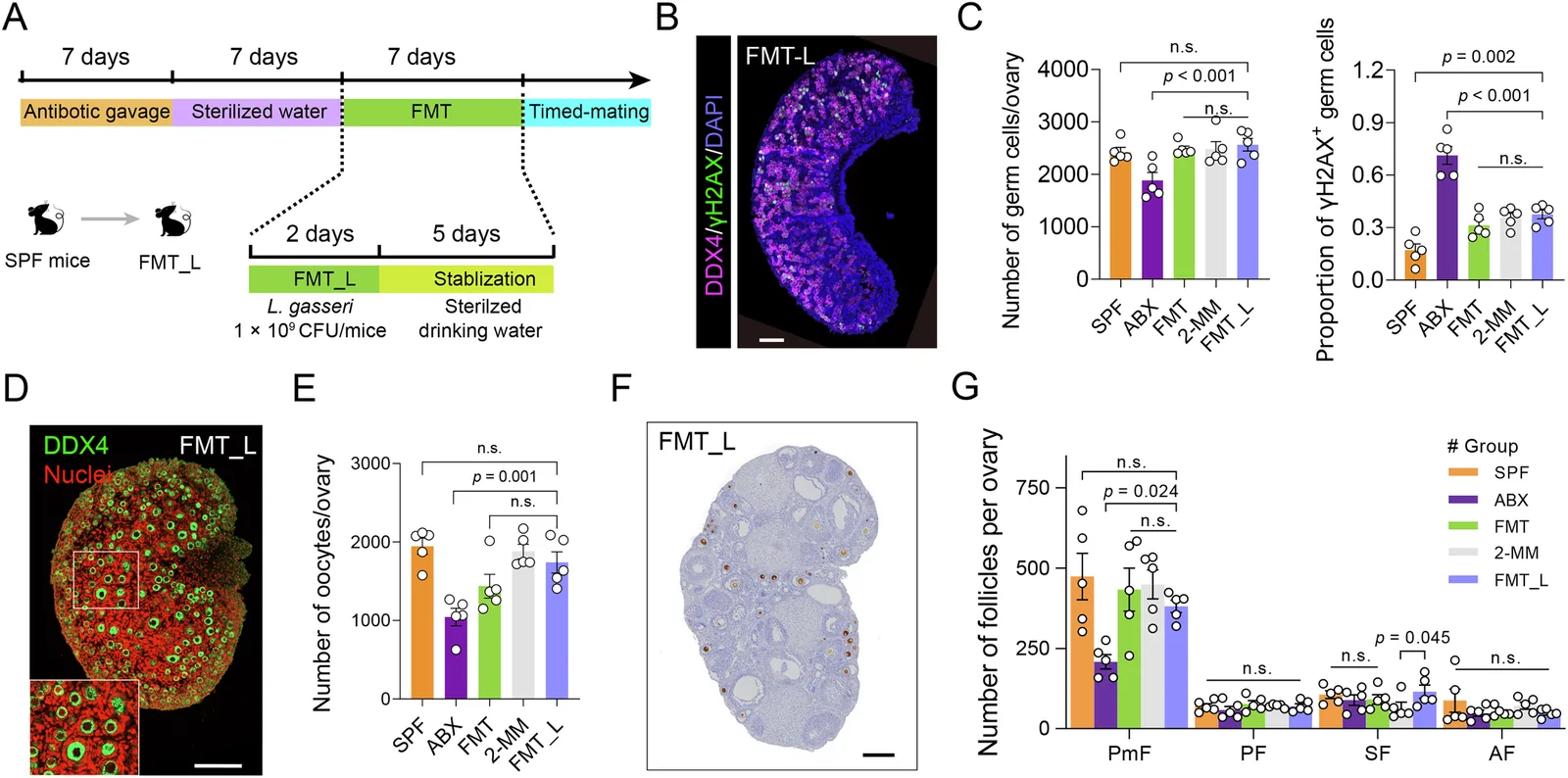
Prevention of maternal microbiome disruption-induced DOR in offspring by *L. gasseri* colonization. (**A**) Brief timeline diagram illustrating the *L. gasseri* colonization procedure. (**B**) Immunofluorescence staining of DDX4 (magenta) and γH2AX (green) expression in E14.5 fetal ovaries from FMT_L dams. Scale bars: 50 μm. (**C**) Comparison of the total number of germ cells per ovary and the proportion of γH2AX-positive germ cells in E14.5 ovaries from SPF (*n* = 5), ABX (*n* = 5), FMT (*n* = 5), 2-MM (*n* = 5), and FMT_L (*n* = 5) dams based on immunofluorescence analysis. (**D**) DDX4 (green) staining in P3 ovaries of offspring from FMT_L dams. PI (red) was used to stain nuclei. Scale bars: 100 μm. (**E**) Comparison of the total number of germ cells per ovary in P3 ovaries from SPF (*n* = 5), ABX (*n* = 5), FMT (*n* = 5), 2-MM (*n* = 5), and FMT_L (*n* = 5) dams. (**F**) Representative immunohistochemical staining of DDX4 in the ovaries of 6-week-old offspring from FMT_L dams. Scale bars: 200 μm. (**G**) Comparison of the number of ovarian follicles at different stages of development in the ovaries of 6-week-old offspring from SPF (*n* = 5), ABX (*n* = 5), FMT (*n* = 5), 2-MM (*n* = 5), and FMT_L (*n* = 5) dams. Data points in (**C**, **E**, **G**) represent offspring from different dams. Data were presented as mean ± SEM. Statistical significance was determined by one-way ANOVA followed by LSD multiple comparison test (**C**,** E**) and two-way ANOVA followed by Tukey’s multiple comparisons test (**G**). Source data are available online for this figure.

We next investigated whether specific microbiome components were involved in maternal microbiome disruption-induced DOR. As disruption of the maternal microbiome induced DOR in offspring in both CD1 and C57BL/6 mice, we reasoned that particular bacteria may be involved in regulating ovarian reserve establishment in both. Based on this hypothesis, we applied 16S amplicon sequencing analysis of the gut microbiota from the SPF group of both strains. Notably, analyzing genus-level abundance unveiled that *Lactobacillus* was the dominant genera in both mouse strains (Fig. EV5A,B), thereby indicating a potential role of *Lactobacillus* genera in mediating maternal microbiome disruption-induced DOR. Importantly, *L. gasseri* of the *Lactobacillus* genera has been demonstrated to promote bile acid excretion (Foley et al, 2021; Usman and Hosono, 2001), and together with our findings above that supplementation of key metabolites involved in bile acid metabolism alleviated maternal microbiome disruption-induced DOR, we next investigated whether colonization of *Lactobacillus* genera probiotic *L. gasseri* (hereafter termed the FMT_L group) in ABX mice rescued maternal microbiome disruption-induced DOR in F1 offspring (Fig. 5A) and found it did so (Fig. 5B–G). Specifically, compared with the fetuses from ABX dams, colonization of *L. gasseri* in ABX dams abrogated maternal microbiome disruption-induced germ cell loss at E14.5 (Fig. 5C, left panel, *p* < 0.001). Moreover, it was observed that germ cells at E14.5 showed a significantly decreased percentage of germ cells expressing γH2AX (Fig. 5C, right panel, *p* < 0.001).

Furthermore, when evaluating ovarian reserve establishment in P3 mouse ovaries in the FMT_L group, we also observed that colonization of *L. gasseri* in ABX dams alleviated the adverse effects of maternal microbiome disruption-induced DOR, and the number of oocytes per ovary was restored to levels similar to those in the FMT group (Fig. 5D,E). In the 6-week-old offspring ovaries, we observed a greater number of PmF in ovaries from offspring of FMT_L dams (Fig. 5F). Counting the ovarian follicles per ovary further confirmed that colonization of *L. gasseri* in ABX dams significantly elevated the ovarian follicles at the PmF stage (Fig. 5G, *p* = 0.024). Overall, these results demonstrated that *L. gasseri* may be the dominant bacterium involved in rescuing effects of maternal microbiome disruption-induced DOR.

### Maternal microbiome modulates oocyte quality in offspring

**Figure 6:**
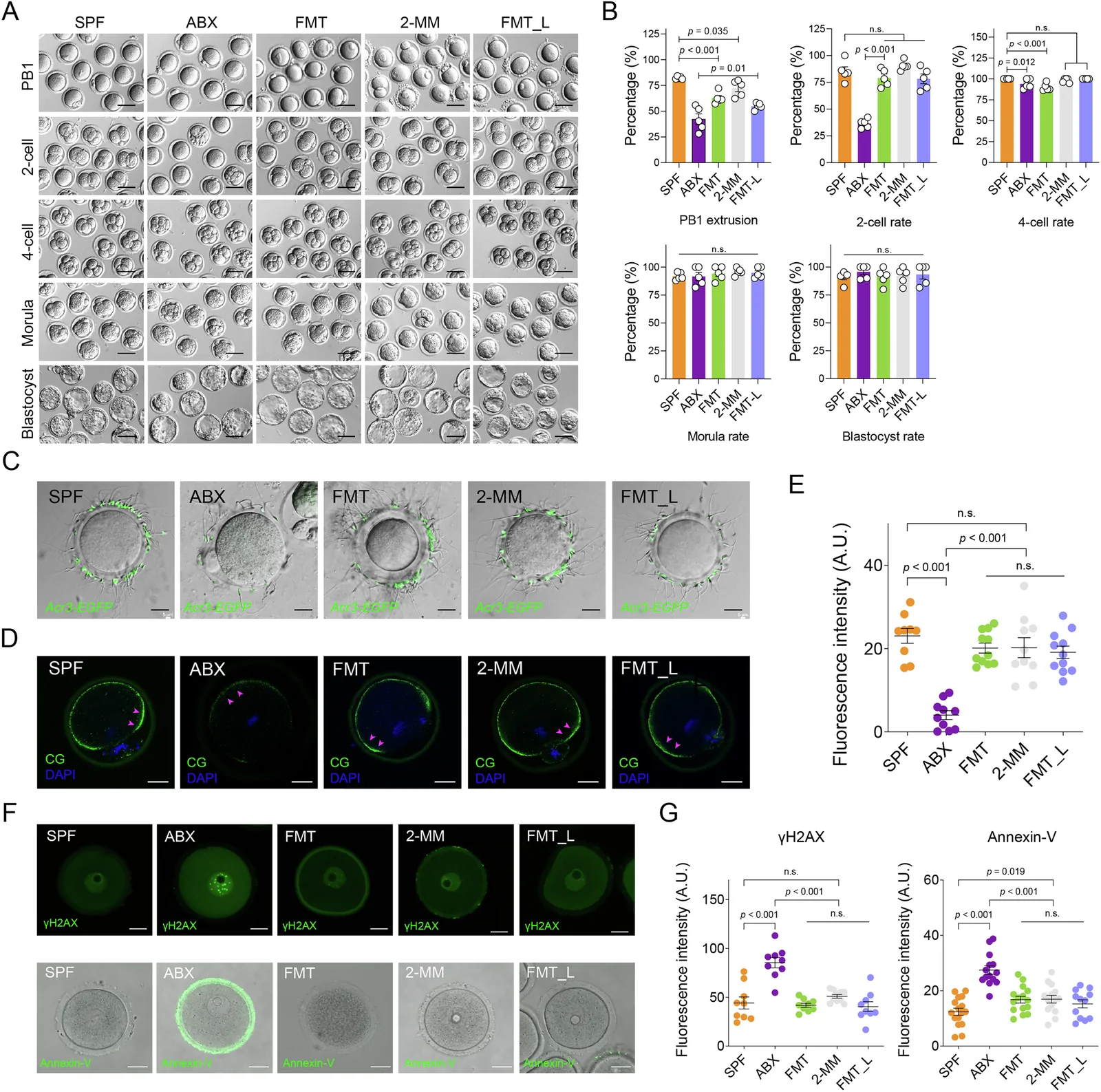
Maternal microbiome disruption induced decreased fertilization capacity and embryonic development potential of oocytes in offspring. (**A**) Representative images of PB1 and early embryos using oocytes collected from offspring of the SPF, ABX, FMT, 2-MM, and FMT_L dams. Scale bars: 100 μm. (**B**) Comparative analysis of PB1, fertilization, 4-cell, morula, and blastocyst rates using oocytes collected from offspring of the SPF, ABX, FMT, 2-MM, and FMT_L dams. The proportion of oocytes at each developmental stage was calculated using the total number of oocytes that reached the preceding stage as the denominator. At least five independent repeats were included for each group. (**C**) Representative images of sperm binding to the zona pellucida surrounding oocytes at metaphase II using oocytes collected from offspring of SPF, ABX, FMT, 2-MM, and FMT_L dams. Sperm were collected from the same donor mice with equalized sperm concentration, and pictures were taken 1 h after in vitro fertilization. Scale bars: 20 μm. (**D**) Representative images of CG distribution in oocytes at metaphase II from offspring of the SPF, ABX, FMT, 2-MM, and FMT_L dams. Scale bars: 20 μm. (**E**) Comparison of the CG signal fluorescence intensity in oocytes at metaphase II collected from offspring of SPF (*n* = 9), ABX (*n* = 10), FMT (*n* = 11), 2-MM (*n* = 10), and FMT_L (*n* = 11) dams. AU arbitrary units. (**F**) Representative immunofluorescence images of γH2AX (top panel) and Annexin-V (bottom panel) expression in oocytes from offspring of the SPF, ABX, FMT, 2-MM, and FMT_L dams. (**G**) Comparison of the γH2AX (left panel, *n* = 9, 9, 9, 10, 9 for SPF, ABX, FMT, 2‑MM, and FMT_L, respectively) and Annexin-V (right panel, *n* = 16, 14, 15, 14, 12 for SPF, ABX, FMT, 2‑MM, and FMT_L, respectively) staining intensity in oocytes from offspring of SPF, ABX, FMT, 2-MM, and FMT_L dams. Data points in (**B**, **E**, **G**) represent offspring from different dams. Data were presented as mean ± SEM. Statistical significance was determined by one-way ANOVA followed by the LSD multiple comparison test. Source data are available online for this figure.

Colonization of *L. gasseri* and supplementation of 2-MM in the ABX dams increased the ovarian reserve in their offspring, and the percentage of γH2AX germ cells in the fetal ovaries of the FMT, FMT_L, and 2-MM dams was significantly decreased; however, when compared with offspring from SPF dams, the percentage remained higher (Fig. 5C, right panel). We further reasoned whether oocyte quality in these groups was compromised in offspring. To this end, we compared the fertilization capacity and early embryo development ability of oocytes isolated from offspring of different groups (Fig. 6A,B; Table EV3). The results showed that oocytes from offspring of ABX dams had the lowest maturation and fertilization rate, indicating compromised development. Notably, after FMT, FMT_L, and 2-MM supplementation, the adverse effects were alleviated (Fig. 6B). For the late stage of embryonic development, no obvious differences were observed among these five groups, indicating that disruption of the maternal microbiome mainly affected early oocyte maturation and fertilization capacity in offspring.

**Figure EV6:**
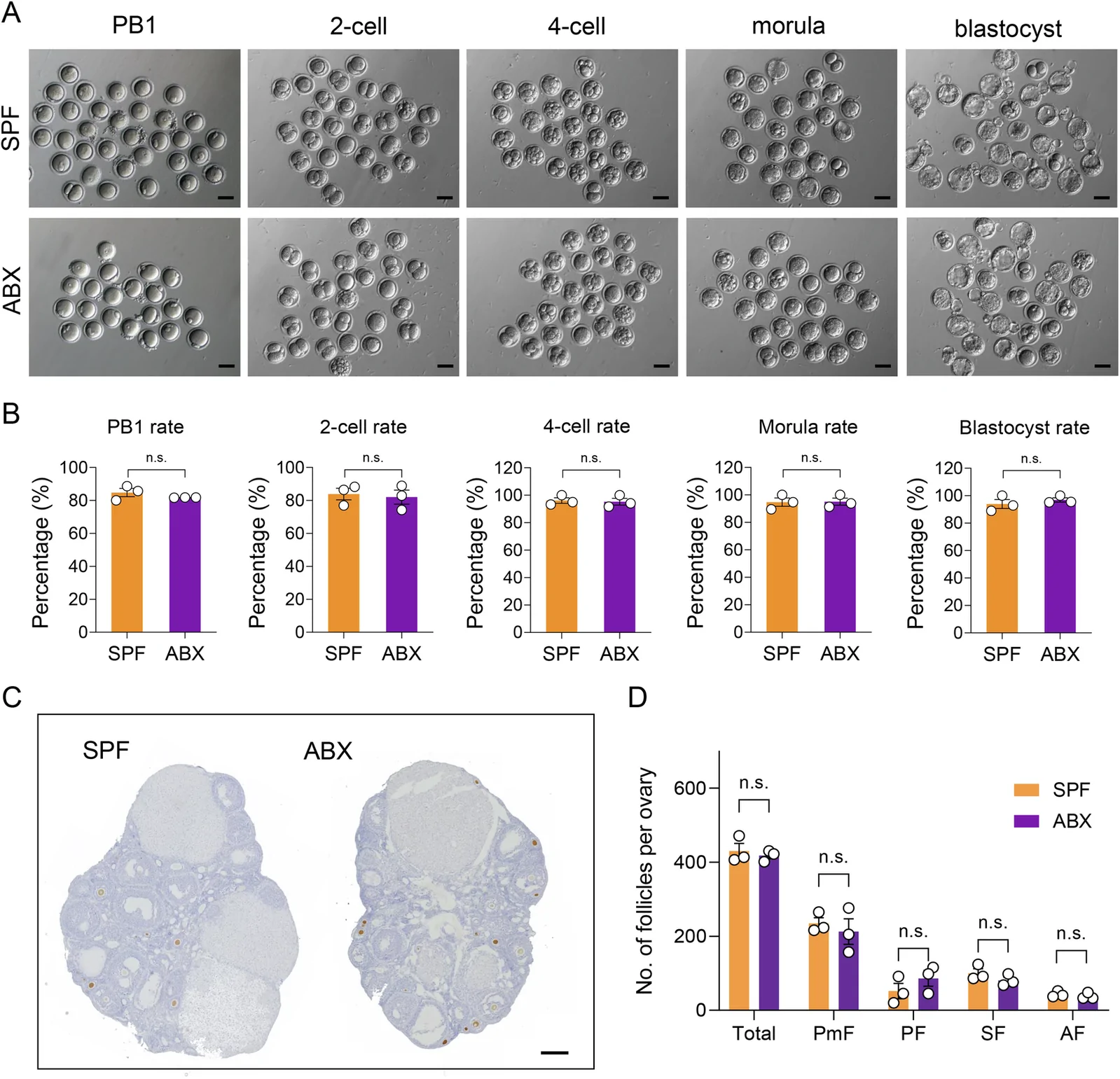
Comparison of the developmental ability of oocytes from SPF and ABX dams. (**A**) Representative images of PB1 and early embryos using oocytes collected from SPF and ABX mice. Scale bars: 100 μm. (**B**) Comparative analysis of PB1, 2-cell, 4-cell, morula, and blastocyst rates using oocytes collected from SPF and ABX dams. At least three repeats were included for each group. (**C**) Immunohistochemical staining of DDX4 in the ovaries of SPF and ABX dams. Ovarian tissues were collected from the dams of the adult offspring. Scale bars: 200 μm. (**D**) Quantification of the total number of ovarian follicles and the number of ovarian follicles at different stages of development in the ovaries of SPF (*n* = 3) and ABX (*n* = 3) dams. Data points in (**B**) represent different mice. Data are presented as mean ± SEM. Statistical significance was determined by a two-tailed unpaired *t*-test.

Furthermore, to investigate whether the DOR phenotype or compromised oocyte quality in offspring may be affected by decreased maternal oocyte quality, we compared the fertilization and early embryo development capacity of oocytes isolated from the SPF and ABX dams; the results confirmed their similarity (Fig. EV6A,B). Besides, we investigated whether antibiotic-mediated disruption of the maternal microbiome directly affected the maternal ovarian reserve. To this end, we sectioned ovarian tissues from dams for histological analysis, and the results showed that the total number of ovarian follicles and the number of follicles at different stages of folliculogenesis were not statistically different between SPF and ABX groups (Fig. EV6C,D). Together, these results suggest that the DOR phenotype in the offspring of ABX dams may be mainly contributed by maternal microbiome disruption, but not decreased maternal oocyte quality.

Next, we investigated how disruption of the maternal microbiome decreased early oocyte maturation and fertilization capacity in offspring. To this end, we performed a sperm binding assay using transgenic mice with sperm carrying a green fluorescent protein (GFP) in their acrosome (Hasuwa et al, 2010). Notably, incubating oocytes with the same concentration of sperm from the same donor mice during in vitro fertilization (IVF) revealed that oocytes from the offspring of ABX dams showed a decreased number of sperm with head binding to the zona pellucida of oocytes, while for the FMT, FMT_L, and 2-MM groups, the number increased (Fig. 6C). These results therefore suggest that disruption of the maternal microbiome compromised the sperm-binding capacity of oocytes in offspring. We further performed a cortical granule (CG) staining assay to explore CG dynamics as oocyte cytoplasmic maturation was associated with fertilization capacity (Fig. 6D) (Miao et al, 2020). In the oocytes of ABX dam offspring, the fluorescence intensity of CGs was significantly decreased and showed compromised distribution in the oocyte subcortical region (Fig. 6E). For the SPF group, CG signals were evenly distributed in the oocyte subcortical region and showed significantly higher intensity signals compared with the ABX group. More importantly, it was found that after FMT and supplementation of key metabolites (including FMT, FMT_L, and 2-MM groups), the fluorescence intensity of CG signals was significantly elevated (Fig. 6E). These results together suggest that maternal microbiome disruption impairs cytoplasmic maturation and sperm-binding capacity in oocytes of adult offspring, which in turn leads to compromised maturation and fertilization rates during early embryo development.

We further analyzed the expression level of γH2AX in oocytes of adult offspring, as maternal microbiome disruption induced elevated levels of γH2AX expression in E14.5 germ cells (Fig. 6F, top panel). Notably, the results also showed elevated levels of γH2AX expression in oocytes of ABX offspring (Fig. 6G, left panel), while after FMT, FMT_L, and 2-MM supplementation, the expression levels of γH2AX were significantly decreased. It’s therefore plausible that the accumulated DNA damage levels persist to adulthood in ABX offspring, which is consistent with previous findings that DNA damage in fetal oocytes can remain unrepaired through ovarian follicle formation and into adulthood due to limited DNA double-strand break repair capacity in oocytes during perinatal and adult stages (Carroll and Marangos, 2013; Sun et al, 2024; Zhu et al, 2024). Because high levels of γH2AX in oocytes usually result in apoptosis and impair embryonic developmental ability (Kerr et al, 2012; Miao et al, 2020), we next detected apoptosis levels in oocytes in adult offspring through Annexin-V staining (Fig. 6F, bottom panel). The results showed that oocytes from offspring of ABX dams showed the highest levels of Annexin-V staining intensity (Fig. 6G, right panel), indicating increased levels of oocyte apoptosis, while this was not the case for FMT, FMT_L, and 2-MM groups. Taken together, these results suggest that maternal microbiome disruption increases DNA damage in fetal germ cells, and this accumulated DNA damage may persist into adulthood in offspring, leading to elevated apoptosis and ultimately compromising oocyte maturation and fertilization capacity.

## Discussion

Antibiotic drugs are commonly used during daily life, however, increasing evidence shows that they are major sources of disturbance to the human gut microbiome (Fishbein et al, 2023; Patangia et al, 2022); for example, humans are prescribed a combination of antibiotics during *Helicobacter pylori* treatment (Graham and Fischbach, 2010; Matsumoto et al, 2019). Specifically, a recent meta-analysis investigating the impact of *Helicobacter pylori* treatment on the human gut microbiota demonstrated that antibiotic exposure significantly reduced the microbiota diversity in nearly all studies, and importantly, these effects can last for approximately one year (Du et al, 2024). Together with the fact that the maternal microbiome plays a critical role in mediating fetal in-utero development, disturbance to the maternal microbiome may cause adverse effects even during the adulthood of the offspring. It is therefore important to systematically investigate how the maternal microbiome affects in-utero fetal development. Here, using an antibiotic-induced microbiome disruption, we verified that antibiotic-mediated maternal gut dysbiosis induced DOR in offspring. In-depth analysis demonstrated that the maternal microbiome may modulate fetal ovarian reserve establishment through microbially mediated bile acid metabolism.

Similar to the murine scenario, the establishment of human ovarian reserve initiates at the embryonic stage (Ford et al, 2020; Ge et al, 2019). Thus, together with the fact that 40% of pregnant women receive antibiotics during pregnancy, it is therefore plausible that maternal microbiome disturbance may induce underappreciated adverse effects on the fetal ovaries in humans. In this regard, more clinical data are needed to systematically evaluate whether the observed DOR phenotypes in mice also occur in a human scenario. Importantly, by applying FMT and selected metabolite supplementation during the critical stage of pregnancy, we showed that maternal microbiome disruption-induced DOR phenotypes can be alleviated. This is of particular significance as it opens the possibility of developing targeted therapeutic strategies to treat the adverse effects of maternal microbiota disruption on offspring sterility, especially in cases where antibiotic use is unavoidable during pregnancy. However, how bile acid metabolites in the maternal serum specifically target germ cells in the fetal ovaries requires further investigation. In this regard, the recent development of uterus-on-a-chip technology in vitro (Xiao et al, 2017) is a promising tool that may help unveil the underlying mechanism between bile acid metabolites and the reproductive system.

Through metabolomics profiling and selected metabolite supplementation, we unveiled a critical role of microbially derived metabolites involved in bile acid metabolism in mediating in-utero ovarian reserve establishment in offspring. Comparatively, it is notable that *L. gasseri* transplantation showed modest rescuing effects, while FMT of small intestine luminal contents from aged-mated SPF mice had a greater rescuing effect. These results suggest that other maternal microbiota involved in bile acid metabolism may also play a role in this process. Supporting this hypothesis, a recent study by Munyoki et al reported that postnatal gut microbiota colonization abrogates DOR phenotypes in germ-free mice (Munyoki et al, 2025). Further investigation revealed that postnatal gut microbiota can safeguard offspring ovarian reserve via the regulation of short-chain fatty acids metabolism, also emphasizing the importance of microbiota-derived metabolites in establishing ovarian reserve. Notably, the establishment of ovarian reserve in mammals is initiated at the embryonic stage and completed shortly after birth (Ge et al, 2019; Rodrigues et al, 2021). Together with our findings here, we propose a two-stage hypothesis in which microbiota regulates ovarian reserve establishment: during the fetal stage, maternal microbiota-derived metabolites regulate the in-utero establishment of the ovarian reserve, whereas after birth, the maturation of the gut microbiome in offspring contributes to postnatal ovarian reserve establishment. However, the differences between maternal microbiota-derived metabolites and metabolites derived from offspring’s gut microbiota, as well as how they collectively promote the ovarian reserve establishment, remain to be investigated.

Besides, a recent work by Zhang et al reported that spermidine supplementation rescues compromised oocyte cytoplasmic maturation capacity in aged mice (Zhang et al, 2023). Combined with our metabolomic profiling, which showed reduced spermidine levels in the maternal serum of maternal microbiota-disrupted dams, it is plausible that further supplementation with spermidine on top of 2-MM may further elevate oocyte quality in offspring. This is an important issue that warrants further exploration, as it addresses the restoration of reproductive capacity in offspring of maternal microbiota-disrupted dams by improving both the quantity and quality of their oocytes.

In conclusion, our study here demonstrates that the maternal microbiome during pregnancy plays an important role in mediating the ovarian reserve establishment in offspring, and the perturbation of the maternal microbiome induces DOR in offspring. Importantly, through FMT and selected metabolite supplementation, we show that the maternal microbiome may regulate in-utero fetal ovarian reserve establishment through microbially mediated bile acid metabolism in the maternal serum. Our findings here highlight the critical role of the maternal microbiome on the fertility of offspring and will have implications for developing potential strategies for addressing maternal microbiota dysbiosis-associated fertility decline in offspring.

While our findings here support the conclusion that antibiotic-mediated maternal gut dysbiosis induces diminished ovarian reserve in offspring, there are some limitations of our study. Firstly, as we indicated in the discussion section, the rescuing effects of FMT and key metabolite supplementation on the oocyte quality of offspring remain limited; whether combined supplementation with other metabolites, such as spermidine, will abrogate such adverse effects remains to be explored. Secondly, we did not investigate whether maternal microbiome disruption-induced DOR and the decline of oocyte quality in F1 offspring caused adverse effects on fertility in F2 offspring. This is also an interesting aspect that warrants further exploration.

## Methods

**Table:** Reagents and tools table

Reagent/resource	Reference or source	Identifier or catalog number
**Experimental models**		
CD1 (*M. musculus*)	Beijing Vital River Laboratory Animal Technology Co., Ltd	N/A
C57BL/6 (*M. musculus*)	Beijing Vital River Laboratory Animal Technology Co., Ltd	N/A
B6D2-Tg (CAG/Su9-DsRed2, Acr3-EGFP) (*M. musculus*)	PMID: 20224175	Hasuwa et al
**Antibodies**		
Rabbit anti-DDX4, polyclonal	Abcam	Cat # ab13840, RRID: AB_443012
Mouse anti-DDX4, monoclonal	Abcam	Cat # ab27591, RRID: AB_11139638
Rabbit anti-STRA8, monoclonal	Abcam	Cat # ab308124, RRID: AB_3107210
Mouse anti-gamma H2AX, monoclonal	Abcam	Cat # ab26350, RRID: AB_470861
Rabbit anti-GAPDH	Affnity	Cat # Af7021, RRID: AB_2839421
Donkey anti-Rabbit IgG (H + L) Secondary Antibody, Alexa Fluor 555	Abcam	Cat # ab150074, RRID: AB_2636997
Donkey anti-Mouse lgG (H + L) Secondary Antibody, Alexa Fluor 555	Abcam	Cat # ab150106, RRID: AB_2857373
Goat anti-Mouse IgG (H + L) Secondary Antibody, Alexa Fluor 488	Abcam	Cat # ab150113, RRID: AB_2576208
Goat anti-Rabbit IgG (H + L) Secondary Antibody, Alexa Fluor 488	Abcam	Cat # ab150077, RRID: AB_2630356
HRP-conjugated goat anti- rabbit IgG	Beyotime	Cat # A0258, RRID: N/A
**Oligonucleotides and other sequence-based reagents**		
Primers	This study	Table EV4
**Chemicals, enzymes and other reagents**		
TDCA	Sigma	T0875
GUDCA	Sigma	06863
Neomycin	Sigma	N6386
Vancomycin	Sangon Biotech	A600983-001
Ampicillin	Sigma	A9518
Metronidazole	Sigma	M1547
DAPI	MedChem Express	HY-D0814
PMSG	Ningbo Second Hormone Factory	110254564
HCG	Ningbo Second Hormone Factory	110251282
M2 culture media	Sigma	M7167
M16 culture media	Sigma	M7292
TYH media	AibeiBio	M2030
HTF media	AibeiBio	M1130
KSOM medium	AibeiBio	M1430
Goat serum	Boster	AR0009
Triton X-100	Solarbio	T8200
Bovine Serum Albumin	Solarbio	A8020
Propidium Iodide	Beyotime	ST511
4% paraformaldehyde	Solarbio	P1110
DAB staining kit	ZSGB-Bio	ZLI-9017
RIPA lysis solution	Beyotime	P0013B
Collagenase IV	Sigma	C5138
Vectashield anti-fade mounting medium	Vector Lab	H-1000-10
Polyvinylidene difluoride membranes	Millipore	ISEQ00010
QIAquick Gel Extraction Kit	Qiagen	28706×4
BeyoECL Star Chemiluminescence Kit	Beyotime	P0018S
TruSeq® DNA PCR-Free Sample Preparation Kit	Illumina	20015963
LCA-FITC (lens culinaris agglutinin antibody)	Xi’an Ruixi Biological Technology Co., Ltd.	R-Y-C-1014
Annexin-V-FITC Apoptosis Detection Kit	Beyotime	C1062
SPARKeasy Tissue & Cell Rapid RNA Extraction Kit	SparkJade	AC0202-A
SPARKscript Ⅱ RT Plus Kit	SparkJade	AG0304-B
2×SYBR Green qPCR Mix kit	SparkJade	AH0104-B
Phusion® High-Fidelity PCR Master Mix	NEB	M0531L
10× Chromium Single Cell 3’ V3 Gel Beads Kit	10× Genomics	PN-1000075
**Software**		
R	https://www.R-project.org/	v.4.0.3
Seurat	https://satijalab.org/seurat/	v.4.4.0
Velocyto	https://velocyto.org/	v.0.17.17
scVelo	https://scvelo.readthedocs.io/en/stable/	v.0.3.1
CellRanger	https://github.com/10XGenomics/cellranger	v.6.1.2
ImageJ	https://imagej.net/ij/	
FlowJo	FlowJo LLC, BD Biosciences	v.7.6.1
MultiQuant / Analyst	AB SCIEX	
CFX Maestro Software	Bio-Rad Laboratories	v.2.3
Multi Automatic chemiluminescence image analysis system	Tanon	5200
**Other**		
*L. gasseri*	CICC	CICC 24878
Microtome	Leica	RM2235
UHPLC	Agilent Technologies	1290 Infinity LC
QTRAP MS	AB Sciex	AB 6500+
Leica confocal microscope	Leica	TCS SP5 and SP8
Metascape	https://metascape.org/gp/index.html#/main/step1	
BMKcloud platform	https://www.bmkgene.com/	
40 μm cell strainer	BD Falcon	352340
Spectrophotometer	Inesayt	722N
Light microscope	Olympus	BX51
Zeiss confocal microscope	Zeiss	LSM900
Nikon AX confocal microscope	Nikon	Nikon AX
EPS Power Supply	Cytiva	EPS 601
Real-Time PCR System	Bio-Rad Laboratories	CFX96 Touch
FACSAria flow cytometer	BD Biosciences	FACSAria II
ExionLC™ AD UHPLC system	AB SCIEX	ExionLC™ AD
Illumina HiSeq X Ten sequencer	Illumina	
Illumina NovaSeq platform	Illumina	
High-temperature-sterilized chow	Beijing Keao Xieli Feed Co., Ltd	1016706476803973120

### Experimental animals

The CD1 and C57BL/6 mice used in this study were purchased from Beijing Vital River Laboratory Animal Technology Co., Ltd. All mice were maintained in a specific pathogen-free (SPF) animal room with a 12 h light/12 h dark cycle, with free access to food and water before the experiments. All experimental procedures used were approved by the Animal Ethics Committee of Qingdao Agricultural University (No. SYXK-2022-0021).

### Establishment of animal models

ABX mice were established as previously described (Vuong et al, 2020). Briefly, six-week-old mice were randomly divided into five groups, including SPF, ABX, FMT, FMT_L, and 2-MM groups. Before the experiment, the mice were fed sterilized food and water. For experimental groups, all mice were intragastrically gavaged with autoclaved sterile water containing a mixture of neomycin (100 mg/kg, Sigma, N6386), metronidazole (100 mg/kg, Sigma, M1547), and vancomycin (50 mg/kg, Sangon Biotech, A600983-001) twice daily (9:00 h and 17:00 h) continuously for a week, and the control groups were intragastrically gavaged with an equal volume of sterile water twice daily. After that, the mice in the experimental groups were then drinking with sterilized water containing 1 mg/mL ampicillin (Sigma, A9518) for a week to allow the successful generation of ABX mice. Immediately after generation of ABX mice, these mice were subjected to different experiment groups: (1) ABX group with ad libitum access to sterilized water and food; (2) FMT group received 2 days of gavage of small intestine luminal contents from age-matched female SPF mice; (3) FMT_L group received 2 days of gavage of *L. gasseri*; (4) 2-MM group received intraperitoneal injection of metabolites from E6.5 to E16.5 of gestation. To avoid potential stress effects, all groups except the FMT (which received fecal microbiota transplantation) and FMT_L (which received *L. gasseri* transplantation) groups were administered an equivalent volume of saline by gavage. Additionally, all pregnant mice were intraperitoneally injected with an equal volume of solvent as that given to the 2-MM group from E6.5 to E16.5 of gestation. All mice were allowed free access to 60Co-γ irradiation and high-temperature-sterilized chow (Beijing Keao Xieli Feed Co., Ltd., Nutritional Indicators: Moisture ≤ 10%; Crude ash ≤8%; Crude protein ≥25%; Calcium 1.0–1.8%; Crude fat ≥7%; Total phosphorus 0.6–1.2%; Crude fiber ≤5%) and autoclaved water. For timed mating, female mice were mated with SPF males, and the presence of a vaginal plug the next morning was used to confirm pregnancy. After detecting the vaginal plug, the female mice were housed individually in cages with autoclaved bedding.

### Determination of microbiota abundance

To evaluate relative microbiota abundance in different groups, the mice were first humanely euthanized to prepare small intestine luminal contents, of which aliquots of 5 µl were mixed with 5 mL of liquid culture medium and incubated at 37 °C in a constant temperature shaker for 12 h. After incubation, the optical density (OD) at 595 nm was measured using a spectrophotometer (Inesayt, 722N). An empty culture medium was set as the negative control.

### Immunofluorescence of ovarian sections

Immunofluorescence of ovarian sections was performed as recently described (Ge et al, 2023). Briefly, immediately after dissection, the tissues were fixed in 4% paraformaldehyde overnight at 4 °C. Following a dehydration step with gradient alcohol solutions, tissues were then embedded in paraffin and sectioned at 5 μm using a microtome (Leica, RM2235). For the immunofluorescence staining assay, the sections were firstly deparaffinized in a 100% xylene solution for 30 min, followed by hydration through an ethanol series. Then, antigen retrieval was performed using 0.01 M sodium citrate buffer (pH = 6.0) for 10 min at 96 °C. After cooling at room temperature (RT), the tissues were blocked using a 3% BSA (Solarbio, A8020) and 10% goat serum (Boster, AR0009) in 0.05 M Tris-HCl buffer for 30 min at RT. After incubation, primary antibodies diluted in blocking buffer were then added and incubated with the slides at 4 °C overnight. Corresponding secondary antibodies were added the next morning, and the slides were incubated at 37 °C for 45 min. Mounting was performed using a Vectashield anti-fade mounting medium containing DAPI to stain nuclei, and pictures were taken using a Zeiss confocal microscope (Zeiss, LSM900) or Nikon AX confocal microscope (Nikon, AX).

### Immunohistochemistry

Adult ovaries were first embedded with paraffin as described above. Then, the tissues were sectioned at 5 μm using a microtome (Leica, RM2235), followed by rinsing with xylene solution and hydration with an ethanol series. The slides were subjected to the antigen retrieval step using 0.01 M sodium citrate buffer (pH = 6.0) for 10 min at 96 °C, and then incubated with 3% H_2_O_2_ solution to block peroxidase at RT for 10 min. Following three washes with PBS to remove the remaining H_2_O_2_, primary antibodies were then incubated with the slides at 4 °C overnight. The next morning, HRP secondary antibodies were added, and the slides were incubated at 37 °C for 45 min. A DAB staining kit (ZSGB-Bio, ZLI-9017) was used, slides were mounted with neutral resins, and pictures were taken under an Olympus BX51 microscope.

### Quantification of germ cells

To quantify the ovarian reserve, the embedded ovaries were first sectioned at a thickness of 5 μm, then the ovarian sections were immunostained with DDX4 to label germ cells. Quantification of P3 ovarian samples was performed by counting the DDX4-positive germ cells in every fifth section, and on average, 50–60 sections could be obtained for each ovary. For 6-week-old ovaries, ovarian follicles with a clear oocyte nucleus were counted in every eighth section and about 160–240 ovarian sections can be obtained for each ovary. Finally, the total number of DDX4-positive germ cells was used to represent the ovarian reserve.

To characterize the progression of ovarian follicle development, we used a previously established standard (Pedersen and Peters, 1968): (1) Primordial follicles: DDX4-positive not surrounded by granulosa cells or enclosed by a single layer of flattened granulosa cells; (2) Primary follicles: oocytes surrounded by a single layer of uniform cuboidal granulosa cells; (3) Secondary follicles: primary oocytes surrounded by two to three layers of granulosa cells; (4) Antral follicles: primary oocytes surrounded by more than 3 layers of polygonal granulosa cells, with some showing an observable follicular antrum.

### Quantitative reverse transcription PCR (RT-qPCR)

For total RNA extraction, about 10 ovarian tissues were pooled for each sample, and the total RNA was extracted using SPARKeasy Tissue & Cell Rapid RNA Extraction Kit (SparkJade, AC0202-A). Reverse transcription was performed using SPARKscript Ⅱ RT Plus Kit (SparkJade, AG0304-B) following the manufacturer’s instructions. After that, the resulting cDNA was used to prepare a PCR reaction mixture using 2×SYBR Green qPCR Mix kit (SparkJade, AH0104-B), and Real-time qPCR was performed on a CFX96 Touch Real-Time PCR System (Bio-Rad Laboratories), according to the manufacturer’s instructions. Quantification of relative gene expression changes was performed using the comparative cycle threshold method (ΔΔCT). *Gapdh* was used as a loading control, and data analysis was performed using the CFX Maestro Software (Version 2.3, Bio-Rad Laboratories). The primer sequences are listed in Table EV4.

### Flow cytometry analysis

For flow cytometry analysis, the obtained ovaries were first dissociated with 2 mg/mL collagenase IV solution for 15 min at 37 °C. After pipetting, the resulting suspension was filtered with a 40-μm cell strainer to obtain single-cell pellets. After centrifugation, the single-cell pellets were fixed with 4% PFA on ice for 15 min. After two washes with ice-cold PBS, these cells were incubated with PBS containing 0.5% Triton X-100 for permeabilization for 10 min at room temperature. Following two washes with ice-cold PBS, the single-cell pellets were blocked with permeabilization solution supplemented with 10% goat serum (Boster, AR0009) for 30 min at 4 °C. The primary antibody diluted in the blocking buffer was incubated with the cells for 30 min at 4 °C, followed by two washes with permeabilization solution. The corresponding secondary antibody was then added, and the samples were incubated on ice for 15 min. Following two washes with blocking buffer, the resulting single-cell pellets were then counterstained with DAPI (HY-D0814, MedChem Express) for 5 min on ice to stain nuclei. Finally, the single-cell suspension was analyzed with a FACSAria flow cytometer (BD Biosciences). Data visualization was performed using FlowJo v7.6.1 (FlowJo LLC, BD Biosciences) software.

### Western blotting

Western blotting was performed as recently described (Ge et al, 2023). Briefly, after dissection, the ovarian tissues were first washed three times with ice-cold PBS to remove contaminants. Then, the tissues were lysed using a RIPA lysis solution (Beyotime, P0013B). Following electrophoresis on a 10–12% SDS-polyacrylamide gel, the proteins were transferred onto polyvinylidene difluoride membranes (Millipore, ISEQ00010) using the Bio-Rad Trans-Blot. The resulting membranes were then incubated with 5% BSA for 2–3 h at RT for blocking. Subsequently, the membranes were incubated with primary antibodies at 4 °C overnight, and corresponding secondary antibodies were added the next morning. Chemiluminescence was performed using a BeyoECL Star Chemiluminescence Kit (Beyotime, P0018S), and pictures were taken using a Multi Automatic chemiluminescence image analysis system (Tanon, 5200). The quantification of gel bands was analyzed using ImageJ Software (Version 1.52n, NIH).

### Single-cell RNA-sequencing and data analysis

The obtained ovaries were first dissociated using a recently described method (Ge et al, 2021). After that, the resulting single-cell suspension was subjected to single-cell barcoding using a 10× Genomics Chromium controller. A cDNA library was constructed using a 10× Genomics’ Chromium Single Cell 3’ V3 Gel Beads Kit (10× Genomics, PN-1000075) following the manufacturer’s instructions. Sequencing was performed by Novogene, Inc. using an Illumina HiSeq X Ten sequencer with pair-ended 150 bp (PE150) reads generated.

For downstream analysis, the resulting raw sequencing files were first subjected to CellRanger (10× Genomics, v.6.1.2) software for genome alignment and quantification of UMI counts following the user tutorial. Then, downstream visualization was performed using the Seurat pipeline following the standard online tutorials (https://satijalab.org/seurat/) (Butler et al, 2018). For RNA velocity analysis, the *velocyto* package was first used to determine single-cell velocity vectors using the *velocyto run10x* function based on Cellranger output files (La Manno et al, 2018). Subsequently, the *scVelo* package was used to visualize RNA velocity vectors projected on the UMAP plot following the online tutorial (https://scvelo.readthedocs.io/en/stable/), with default parameters (Bergen et al, 2020). GO enrichment was performed using Metascape web tools (https://metascape.org/) with default settings (Zhou et al, 2019).

### 16S rRNA gene sequencing and data analysis

To perform 16S rRNA sequencing, we first extracted the genomic DNA of the small intestine using the CTAB/SDS method. After checking the purity of the genomic DNA using 1% agarose gel, the V3–V4 region of the 16S rRNA gene was amplified using the specific primers. PCR was then performed using a Phusion® High-Fidelity PCR Master Mix (NEB, M0531L) kit following the manufacturer’s instructions. Then, the PCR products were subjected to electrophoresis (Cytiva, EPS 601) on 2% agarose gel for detection and purified using a QIAquick Gel Extraction Kit (Qiagen, 28706×4). Sequencing libraries were generated using the TruSeq® DNA PCR-Free Sample Preparation Kit (Illumina, 20015963) following the manufacturer’s instructions and were sequenced on an Illumina NovaSeq platform. For data visualization, raw 16S rRNA data quality control and downstream visualization were performed using the BMKcloud platform (https://www.bmkgene.com/products/bmkcloud/) with default parameters.

### Targeted metabolomic profiling

For targeted metabolomic profiling, maternal serum samples were analyzed by Shanghai Applied Protein Technology Co., Ltd. (Shanghai, China), while fetal ovarian samples were detected by Beijing Novogene Bioinformatics Technology Co., Ltd. (Tianjin, China). Both analyses were performed using the same AB Sciex QTRAP 6500+ mass spectrometer. Specifically, an Agilent 1290 Infinity LC system was applied for serum samples, while an ExionLC™ AD UHPLC system (AB SCIEX) was applied for fetal ovarian bile acid detection. Briefly, E14.5 dams were fasted with access to water provided one day before sample collection, and the whole blood and fetal ovaries were collected the following day. For serum samples, the serum was obtained by centrifugation at 3000 rpm for 5 min at 4 °C, followed by a second centrifugation at 14,000×*g* for 20 min. After that, the supernatant was collected and vacuum dried, and the obtained samples were then reconstituted in acetonitrile/water (1:1, v/v) and centrifuged at 14,000×*g* for 15 min. Next, the obtained samples were subjected to UHPLC-MS analysis using a UHPLC system (Agilent 1290 Infinity) coupled with AB 6500 + QTRAP mass spectrometer (AB SCIEX).

For ovarian samples, each group contained at least 30 ovaries, and the pooled ovarian tissues were then dissociated into single-cell pellets. The total number of cells was then quantified for normalization of final bile acid concentrations. After centrifugation, the resulting single-cell pellets were freeze-dried and resuspended in 100 μL of water. Ultrasonication was performed for 10 min, and the resulting samples were then homogenized with 300 μL of acetonitrile/methanol at a ratio of (8:2). Following an additional 10 min of ultrasonication, samples were incubated at −20 °C for 60 min, then centrifuged at 12,000 rpm for 10 min. The resulting supernatant was then subjected to UHPLC-MS analysis using a UHPLC system (ExionLC™ AD, AB SCIEX) coupled with an AB 6500 + QTRAP mass spectrometer (AB SCIEX). All procedures were performed according to the manufacturer’s instructions. Finally, MultiQuant or Analyst was applied for data analysis, and differential metabolites were identified using an unpaired Student’s *t*-test, with *p* < 0.05 considered as statistically significant.

### Microbiota transplant and *L. gasseri* colonization

Microbiota transplants were performed as previously described (Zhang et al, 2020). Briefly, small intestine luminal contents (gut microbiota) were first collected from age-matched SPF mice; then, the contents were homogenized and diluted with 20% sterile glycerol at a ratio of 1:1. Subsequently, the resulting suspensions were passed through a 40-μm cell strainer (BD Falcon, 352340) and then diluted tenfold with 0.9% saline prior to gavage. For microbiota transplantation, ABX mice were intragastrically gavaged with 100 μL of the resulting suspension once a day for 2 days. For administration of *L. gasseri*, *L. gasseri* was purchased from the China Center of Industrial Culture Collection (CICC), and about 1 × 10^9^ colony-forming units of *L. gasseri* were transplanted for each mouse via oral gavage once daily for 2 days. After that, the recipient mice were housed in a sterile environment for 5 days for colonization.

### Metabolite supplementation

For metabolite supplementation, 50 mg/kg/d taurodeoxycholic acid (TDCA) (Quante et al, 2021) and 50 mg/kg/d glycoursodeoxycholic acid (GUDCA) (Zhao et al, 2023) mixture was injected intraperitoneally as previously described, and the metabolite mixtures were intraperitoneally injected into E6.5 dams once a day until E16.5. The control group was injected with an equal volume of solvent.

### Oocyte in vitro maturation

Female mice were intraperitoneally injected with 8 IU pregnant mare serum gonadotropin (PMSG; NSHF Inc.) 48 h before being humanely sacrificed. Ovaries were collected for the retrieval of cumulus-oocyte complexes in M2 culture media (Sigma, M7167). Then, the surrounding cumulus cells were removed by repeated pipetting and were cultured for maturation in M16 media (Sigma, M7292) at 37 °C in a CO_2_ incubator containing 5% CO_2_.

### In vitro fertilization (IVF) and sperm binding assay

For IVF analysis, MII oocytes were retrieved following PMSG injection and human chorionic gonadotrophin (HCG; NSHF Inc.) was administered to female mice via intraperitoneal injection at 48 h and 14 h before ovary collection, respectively. Sperm were collected from the cauda epididymides of 10-week-old male mice and were capacitated in TYH media (AibeiBio Inc., M2030) at 37 °C and 5% CO_2_ for 1 h prior to IVF. Subsequently, the obtained oocytes were first transferred to the HTF media (AibeiBio Inc., M1130), and the sperm were then added to allow IVF. Six hours after IVF, the embryos were transferred to KSOM medium (AibeiBio Inc., M1430), and 2-cell rates, 4-cell rates, morula rates, and blastocyst rates were observed after 24, 48, 72, and 80 h cultures, respectively.

For the sperm binding assay, we used a transgenic mouse model with sperm carrying a GFP protein in their head. Specifically, sperm were collected from B6D2-Tg(CAG/Su9-DsRed2, Acr3-EGFP) RBGS002Osb transgenic mice and were capacitated in TYH media at 37 °C and 5% CO_2_ for 1 h before IVF. After that, the MII stage oocytes were then incubated with the capacitated sperm in HTF media at 37 °C and 5% CO_2_ for 1 h. After that, the oocytes were washed with M2 solution to remove unbound sperm and fixed with 4% PFA for 40 min at RT. Finally, the samples were mounted with a Vectashield anti-fade mounting medium containing DAPI to stain nuclei, and pictures were taken using a Leica TCS SP8 confocal microscope.

### Fluorescence staining of cortical granules and γH2AX

After oocyte retrieval, the oocytes were first fixed with 4% paraformaldehyde at room temperature for 40 min and then subjected to the permeabilization procedure. Permeabilization was performed using PBS solution supplemented with 0.5% Triton X-100 at room temperature for 20 min. After that, the samples were blocked with PBS supplemented with 1% BSA at room temperature for 1 h and then incubated with FITC-conjugated lens culinaris agglutinin antibody (LCA-FITC; Xi’an Ruixi Biological Technology Co., Ltd., R-Y-C-1014) for 20 min at RT, or incubated with 1:1000 blocking buffer diluted γH2AX antibody (Abcam, AB26350) overnight at 4 °C. For the γH2AX staining assay, the samples were washed three times with PBS solution supplemented with 0.1% Tween-20 and then incubated with 1:200 blocking buffer diluted with secondary antibody at 37 °C for 1 h. Finally, both samples were mounted with a Vectashield anti-fade mounting medium containing DAPI to stain nuclei, and pictures were taken using a Leica TCS SP8 confocal microscope.

### Annexin-V staining assay

Annexin-V staining assay was performed using an Annexin-V-FITC apoptosis detection kit (Beyotime, C1062) and all procedures were performed following the manufacturer’s instructions. Briefly, the collected oocytes were washed three times with PBS solution supplemented with 0.1% PVA, and then incubated with 1:20 diluted Annexin-V staining buffer for 15 min at 37 °C in a CO_2_ incubator containing 5% CO_2_. After incubation, the samples were transferred into pre-warmed binding buffer and were immediately imaged using a Leica TCS SP5 confocal microscope.

### Statistical analysis

All statistical analyses and graph generation were performed using GraphPad Prism 8 (GraphPad Software, Inc., v8.0.2), and data were presented as mean ± SEM. For comparisons between two groups, a two-tailed Student’s *t*-test was performed to determine statistical differences. For comparisons of two or more groups with one variable, a one-way analysis of variance (ANOVA) followed by LSD multiple comparison tests was applied. For comparisons of two or more groups with two variables, a two-way ANOVA followed by Tukey’s test was used. Significance was determined when *p* < 0.05. No formal blinding was applied during data acquisition or analysis.

## Supplementary information

Table EV1

Table EV2

Table EV3

Table EV4

Peer Review File

Dataset EV1

Source data Fig. 1

Source data Fig. 2

Source data Fig. 3

Source data Fig. 4

Source data Fig. 5

Source data Fig. 6

Expanded View Figures

## Data Availability

Data were available in a public, open-access repository. Single-cell RNA-sequencing data and 16S rRNA gene sequencing generated in this study are deposited in the Genome Sequence Archive at the National Genomics Data Center with the accession numbers CRA010862 (https://ngdc.cncb.ac.cn/gsa/browse/CRA010862) and CRA017793 (https://ngdc.cncb.ac.cn/gsa/browse/CRA017793). The source data of this paper are collected in the following database record: biostudies:S-SCDT-10_1038-S44319-026-00912-3.

## References

[CR1] Anderson EL, Baltus AE, Roepers-Gajadien HL, Hassold TJ, de Rooij DG, van Pelt AM, Page DC (2008) Stra8 and its inducer, retinoic acid, regulate meiotic initiation in both spermatogenesis and oogenesis in mice. Proc Natl Acad Sci USA 105:14976–14980 10.1073/pnas.0807297105 PMC2542382 18799751

[CR2] Bergen V, Lange M, Peidli S, Wolf FA, Theis FJ (2020) Generalizing RNA velocity to transient cell states through dynamical modeling. Nat Biotechnol 38:1408–1414 10.1038/s41587-020-0591-3 32747759

[CR3] Butler A, Hoffman P, Smibert P, Papalexi E, Satija R (2018) Integrating single-cell transcriptomic data across different conditions, technologies, and species. Nat Biotechnol 36:411–420 10.1038/nbt.4096 PMC6700744 29608179

[CR4] Carroll J, Marangos P (2013) The DNA damage response in mammalian oocytes. Front Genet 4:117 10.3389/fgene.2013.00117 PMC3690358 23805152

[CR5] Chen B, Bai Y, Tong F, Yan J, Zhang R, Zhong Y, Tan H, Ma X (2023) Glycoursodeoxycholic acid regulates bile acids level and alters gut microbiota and glycolipid metabolism to attenuate diabetes. Gut Microbes 15:2192155 10.1080/19490976.2023.2192155 PMC10054359 36967529

[CR6] Di Gesu CM, Matz LM, Bolding IJ, Fultz R, Hoffman KL, Gammazza AM, Petrosino JF, Buffington SA (2022) Maternal gut microbiota mediate intergenerational effects of high-fat diet on descendant social behavior. Cell Rep 41:111461 10.1016/j.celrep.2022.111461 PMC9597666 36223744

[CR7] Du L, Chen B, Cheng F, Kim J, Kim JJ (2024) Effects of Helicobacter pylori therapy on gut microbiota: a systematic review and meta-analysis. Dig Dis 42:102–112 10.1159/000527047 36228588

[CR8] Dupont C, Cordier AG, Junien C, Mandon-Pepin B, Levy R, Chavatte-Palmer P (2012) Maternal environment and the reproductive function of the offspring. Theriogenology 78:1405–1414 10.1016/j.theriogenology.2012.06.016 22925651

[CR9] Fishbein SRS, Mahmud B, Dantas G (2023) Antibiotic perturbations to the gut microbiome. Nat Rev Microbiol 21:772–788 10.1038/s41579-023-00933-y PMC12087466 37491458

[CR10] Foley MH, O’Flaherty S, Allen G, Rivera AJ, Stewart AK, Barrangou R, Theriot CM (2021) Lactobacillus bile salt hydrolase substrate specificity governs bacterial fitness and host colonization. Proc Natl Acad Sci USA 118:e2017709118 10.1073/pnas.2017709118 PMC8017965 33526676

[CR11] Ford EA, Beckett EL, Roman SD, McLaughlin EA, Sutherland JM (2020) Advances in human primordial follicle activation and premature ovarian insufficiency. Reproduction 159:R15–R29 10.1530/REP-19-0201 31376814

[CR12] Gao Y, Nanan R, Macia L, Tan J, Sominsky L, Quinn TP, O’Hely M, Ponsonby AL, Tang MLK, Collier F et al (2021) The maternal gut microbiome during pregnancy and offspring allergy and asthma. J Allergy Clin Immunol 148:669–678 10.1016/j.jaci.2021.07.011 34310928

[CR13] Ge W, Li L, Dyce PW, De Felici M, Shen W (2019) Establishment and depletion of the ovarian reserve: physiology and impact of environmental chemicals. Cell Mol Life Sci 76:1729–1746 10.1007/s00018-019-03028-1 PMC11105173 30810760

[CR14] Ge W, Sun YC, Qiao T, Liu HX, He TR, Wang JJ, Chen CL, Cheng SF, Dyce PW, De Felici M et al (2023) Murine skin-derived multipotent papillary dermal fibroblast progenitors show germline potential in vitro. Stem Cell Res Ther 14:17 10.1186/s13287-023-03243-5 PMC9898921 36737797

[CR15] Ge W, Wang JJ, Zhang RQ, Tan SJ, Zhang FL, Liu WX, Li L, Sun XF, Cheng SF, Dyce PW et al (2021) Dissecting the initiation of female meiosis in the mouse at single-cell resolution. Cell Mol Life Sci 78:695–713 10.1007/s00018-020-03533-8 PMC11072979 32367190

[CR16] Graham DY, Fischbach L (2010) Helicobacter pylori treatment in the era of increasing antibiotic resistance. Gut 59:1143–1153 10.1136/gut.2009.192757 20525969

[CR17] Hasuwa H, Muro Y, Ikawa M, Kato N, Tsujimoto Y, Okabe M (2010) Transgenic mouse sperm that have green acrosome and red mitochondria allow visualization of sperm and their acrosome reaction in vivo. Exp Anim 59:105–107 10.1538/expanim.59.105 20224175

[CR18] Hsu CN, Yu HR, Lin IC, Tiao MM, Huang LT, Hou CY, Chang-Chien GP, Lin S, Tain YL (2022) Sodium butyrate modulates blood pressure and gut microbiota in maternal tryptophan-free diet-induced hypertension rat offspring. J Nutr Biochem 108:109090 10.1016/j.jnutbio.2022.109090 35724813

[CR19] Huang F, Cao Y, Liang J, Tang R, Wu S, Zhang P, Chen R (2024) The influence of the gut microbiome on ovarian aging. Gut Microbes 16:2295394 10.1080/19490976.2023.2295394 PMC10766396 38170622

[CR20] Kerr JB, Hutt KJ, Michalak EM, Cook M, Vandenberg CJ, Liew SH, Bouillet P, Mills A, Scott CL, Findlay JK et al (2012) DNA damage-induced primordial follicle oocyte apoptosis and loss of fertility require TAp63-mediated induction of Puma and Noxa. Mol Cell 48:343–352 10.1016/j.molcel.2012.08.017 PMC3496022 23000175

[CR21] Kerr JB, Myers M, Anderson RA (2013) The dynamics of the primordial follicle reserve. Reproduction 146:R205–R215 10.1530/REP-13-0181 23929903

[CR22] Kimura I, Miyamoto J, Ohue-Kitano R, Watanabe K, Yamada T, Onuki M, Aoki R, Isobe Y, Kashihara D, Inoue D et al (2020) Maternal gut microbiota in pregnancy influences offspring metabolic phenotype in mice. Science 367:eaaw8429 10.1126/science.aaw8429 32108090

[CR23] Kiselev VY, Kirschner K, Schaub MT, Andrews T, Yiu A, Chandra T, Natarajan KN, Reik W, Barahona M, Green AR et al (2017) SC3: consensus clustering of single-cell RNA-seq data. Nat Methods 14:483–486 10.1038/nmeth.4236 PMC5410170 28346451

[CR24] La Manno G, Soldatov R, Zeisel A, Braun E, Hochgerner H, Petukhov V, Lidschreiber K, Kastriti ME, Lonnerberg P, Furlan A et al (2018) RNA velocity of single cells. Nature 560:494–498 10.1038/s41586-018-0414-6 PMC6130801 30089906

[CR25] Mah LJ, El-Osta A, Karagiannis TC (2010) gammaH2AX: a sensitive molecular marker of DNA damage and repair. Leukemia 24:679–686 10.1038/leu.2010.6 20130602

[CR26] Malki S, van der Heijden GW, O’Donnell KA, Martin SL, Bortvin A (2014) A role for retrotransposon LINE-1 in fetal oocyte attrition in mice. Dev Cell 29:521–533 10.1016/j.devcel.2014.04.027 PMC4056315 24882376

[CR27] Man L, Lustgarten Guahmich N, Vyas N, Tsai S, Arazi L, Lilienthal D, Schattman G, Rosenwaks Z, James D (2022) Ovarian reserve disorders, can we prevent them? A review. Int J Mol Sci 23:15426 10.3390/ijms232315426 PMC9737352 36499748

[CR28] Matsumoto H, Shiotani A, Graham DY (2019) Current and future treatment of Helicobacter pylori infections. Adv Exp Med Biol 1149:211–225 10.1007/5584_2019_367 PMC6918954 31016626

[CR29] Miao Y, Cui Z, Gao Q, Rui R, Xiong B (2020) Nicotinamide mononucleotide supplementation reverses the declining quality of maternally aged oocytes. Cell Rep 32:107987 10.1016/j.celrep.2020.107987 32755581

[CR30] Miyoshi J, Hisamatsu T (2022) The impact of maternal exposure to antibiotics on the development of child gut microbiome. Immunol Med 45:63–68 10.1080/25785826.2021.1963189 34392799

[CR31] Morel C, Martinez Sanchez I, Cherifi Y, Chartrel N, Diaz Heijtz R (2023) Perturbation of maternal gut microbiota in mice during a critical perinatal window influences early neurobehavioral outcomes in offspring. Neuropharmacology 229:109479 10.1016/j.neuropharm.2023.109479 36870672

[CR32] Munyoki SK, Goff JP, Reshke A, Wilderoter E, Mafarachisi N, Kolobaric A, Sheng Y, Mullett SJ, King GE, DeSchepper JD et al (2025) The microbiota extends the reproductive lifespan of mice by safeguarding the ovarian reserve. Cell Host Microbe 33:1731–1747.e8 10.1016/j.chom.2025.09.006 PMC13374525 41005310

[CR33] Nyangahu DD, Lennard KS, Brown BP, Darby MG, Wendoh JM, Havyarimana E, Smith P, Butcher J, Stintzi A, Mulder N et al (2018) Disruption of maternal gut microbiota during gestation alters offspring microbiota and immunity. Microbiome 6:124 10.1186/s40168-018-0511-7 PMC6035804 29981583

[CR34] Park SU, Walsh L, Berkowitz KM (2021) Mechanisms of ovarian aging. Reproduction 162:R19–R33 10.1530/REP-21-0022 PMC9354567 33999842

[CR35] Patangia DV, Anthony Ryan C, Dempsey E, Paul Ross R, Stanton C (2022) Impact of antibiotics on the human microbiome and consequences for host health. Microbiologyopen 11:e1260 10.1002/mbo3.1260 PMC8756738 35212478

[CR36] Pedersen T, Peters H (1968) Proposal for a classification of oocytes and follicles in the mouse ovary. J Reprod Fertil 17:555–557 10.1530/jrf.0.0170555 5715685

[CR37] Perera F, Herbstman J (2011) Prenatal environmental exposures, epigenetics, and disease. Reprod Toxicol 31:363–373 10.1016/j.reprotox.2010.12.055 PMC3171169 21256208

[CR38] Qi X, Yun C, Sun L, Xia J, Wu Q, Wang Y, Wang L, Zhang Y, Liang X, Wang L et al (2019) Gut microbiota-bile acid-interleukin-22 axis orchestrates polycystic ovary syndrome. Nat Med 25:1225–1233 10.1038/s41591-019-0509-0 PMC7376369 31332392

[CR39] Quante M, Iske J, Heinbokel T, Desai BN, Cetina Biefer HR, Nian Y, Krenzien F, Matsunaga T, Uehara H, Maenosono R et al (2021) Restored TDCA and valine levels imitate the effects of bariatric surgery. Elife 10:e62928 10.7554/eLife.62928 PMC8257250 34155969

[CR40] Rasool S, Shah D (2017) Fertility with early reduction of ovarian reserve: the last straw that breaks the Camel’s back. Fertil Res Pract 3:15 10.1186/s40738-017-0041-1 PMC5637249 29046817

[CR41] Rodrigues P, Limback D, McGinnis L, Marques M, Aibar J, Plancha CE (2021) Germ-somatic cell interactions are involved in establishing the follicle reserve in mammals. Front Cell Dev Biol 9:674137 10.3389/fcell.2021.674137 PMC8236641 34195191

[CR42] Saitou M, Yamaji M (2012) Primordial germ cells in mice. Cold Spring Harb Perspect Biol 4:a008375 10.1101/cshperspect.a008375 PMC3536339 23125014

[CR43] Sanidad KZ, Amir M, Ananthanarayanan A, Singaraju A, Shiland NB, Hong HS, Kamada N, Inohara N, Nunez G, Zeng MY (2022) Maternal gut microbiome-induced IgG regulates neonatal gut microbiome and immunity. Sci Immunol 7:eabh3816 10.1126/sciimmunol.abh3816 PMC9375732 35687695

[CR44] Sun F, Ali NN, Londono-Vasquez D, Simintiras CA, Qiao H, Ortega MS, Agca Y, Takahashi M, Rivera RM, Kelleher AM et al (2024) Increased DNA damage in full-grown oocytes is correlated with diminished autophagy activation. Nat Commun 15:9463 10.1038/s41467-024-53559-w PMC11530536 39487138

[CR45] Sun Z, Lee-Sarwar K, Kelly RS, Lasky-Su JA, Litonjua AA, Weiss ST, Liu YY (2023) Revealing the importance of prenatal gut microbiome in offspring neurodevelopment in humans. EBioMedicine 90:104491 10.1016/j.ebiom.2023.104491 PMC9996363 36868051

[CR46] Tai SK, Lin YH, Lin CH, Lin MC (2024) Antibiotic exposure during pregnancy increases risk for childhood atopic diseases: a nationwide cohort study. Eur J Med Res 29:189 10.1186/s40001-024-01793-9 PMC10953187 38504329

[CR47] Usman, Hosono A (2001) Hypocholesterolemic effect of Lactobacillus gasseri SBT0270 in rats fed a cholesterol-enriched diet. J Dairy Res 68:617–624 10.1017/s0022029901005179 11928958

[CR48] Vuong HE, Pronovost GN, Williams DW, Coley EJL, Siegler EL, Qiu A, Kazantsev M, Wilson CJ, Rendon T, Hsiao EY (2020) The maternal microbiome modulates fetal neurodevelopment in mice. Nature 586:281–286 10.1038/s41586-020-2745-3 PMC7554197 32968276

[CR49] Wilkosz P, Greggains GD, Tanbo TG, Fedorcsak P (2014) Female reproductive decline is determined by remaining ovarian reserve and age. PLoS One 9:e108343 10.1371/journal.pone.0108343 PMC4195570 25310678

[CR50] Xiao S, Coppeta JR, Rogers HB, Isenberg BC, Zhu J, Olalekan SA, McKinnon KE, Dokic D, Rashedi AS, Haisenleder DJ et al (2017) A microfluidic culture model of the human reproductive tract and 28-day menstrual cycle. Nat Commun 8:14584 10.1038/ncomms14584 PMC5379057 28350383

[CR51] Zhang P, Liu J, Xiong B, Zhang C, Kang B, Gao Y, Li Z, Ge W, Cheng S, Hao Y et al (2020) Microbiota from alginate oligosaccharide-dosed mice successfully mitigated small intestinal mucositis. Microbiome 8:112 10.1186/s40168-020-00886-x PMC7382812 32711581

[CR52] Zhang Y, Bai J, Cui Z, Li Y, Gao Q, Miao Y, Xiong B (2023) Polyamine metabolite spermidine rejuvenates oocyte quality by enhancing mitophagy during female reproductive aging. Nat Aging 3:1372–1386 10.1038/s43587-023-00498-8 37845508

[CR53] Zhao Q, Dai MY, Huang RY, Duan JY, Zhang T, Bao WM, Zhang JY, Gui SQ, Xia SM, Dai CT et al (2023) Parabacteroides distasonis ameliorates hepatic fibrosis potentially via modulating intestinal bile acid metabolism and hepatocyte pyroptosis in male mice. Nat Commun 14:1829 10.1038/s41467-023-37459-z PMC10067939 37005411

[CR54] Zhou Y, Zhou B, Pache L, Chang M, Khodabakhshi AH, Tanaseichuk O, Benner C, Chanda SK (2019) Metascape provides a biologist-oriented resource for the analysis of systems-level datasets. Nat Commun 10:1523 10.1038/s41467-019-09234-6 PMC6447622 30944313

[CR55] Zhu J, Guo S, Cao J, Zhao H, Ma Y, Zou H, Ju H, Liu Z, Li J (2024) Epigenetic modifications are involved in transgenerational inheritance of cadmium reproductive toxicity in mouse oocytes. Int J Mol Sci 25:10996 10.3390/ijms252010996 PMC11507422 39456778

