# Supplementary material for: Maternal microbiome promotes offspring ovarian reserve via bile acid metabolism
Source: EMBO Rep. 2026 Aug 27;27(20):5752–78. doi: 10.1038/s44319-026-00912-3 (PMC13694719; doi:10.1038/s44319-026-00912-3)
Supplement: Supplementary file 1 [file 44319_2026_912_MOESM13_ESM.pdf]

Expanded View Figures

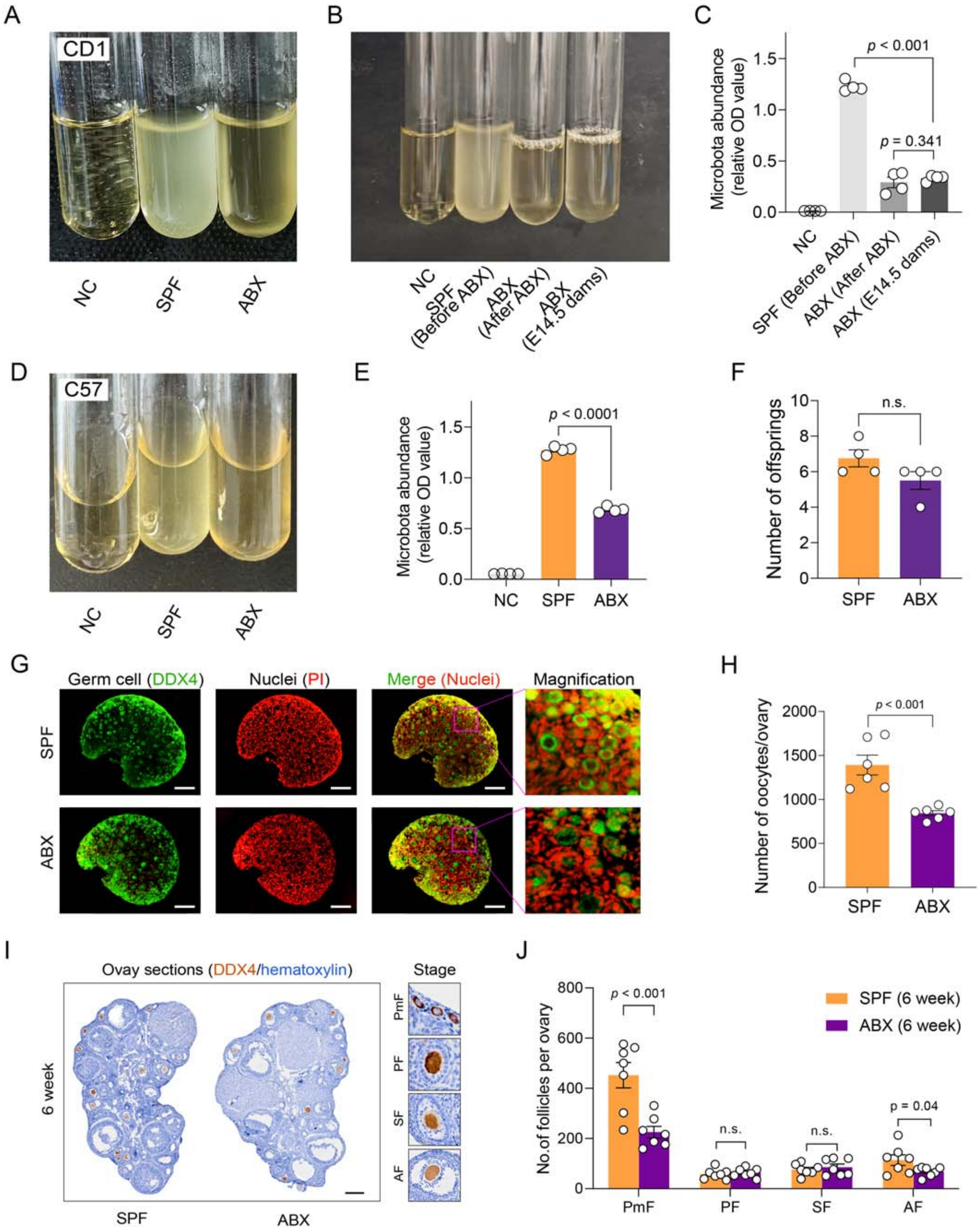

Figure EV1. Validation of the ABX mouse model and comparison of ovarian reserve in offspring mice between SPF and ABX groups.

(**A**) Representative images of bacterial cultures from the small intestine luminal contents of different groups of CD1 mice after antibiotic treatment. Images were captured after 12 h of in vitro culture. (**B**) Representative images of bacterial cultures from small intestine luminal contents in ABX mice before antibiotic administration ($n = 4$), after antibiotic administration ($n = 4$), and at E14.5 during gestation ($n = 4$). NC ($n = 4$) indicates the negative control. Images were captured after 12 h of in vitro culture. (**C**) Relative bacterial biomass abundance of intestinal contents from SPF (Before ABX), ABX (After ABX) and ABX (E14.5 dams) groups following 12 h of in vitro culture. Data were quantified using a spectrophotometer at 595 nm. (**D**) Representative images of bacterial cultures from the small intestine luminal contents of different groups of C57BL/6 mice after antibiotic treatment. Images were captured after 12 h of in vitro culture. (**E**) Relative bacterial biomass abundance of intestinal contents from different groups in C57BL/6 mice ($n = 4$) after 12 h of in vitro culture. Data were quantified using a spectrophotometer at 595 nm. (**F**) Comparison of the number of offspring in SPF ($n = 4$) and ABX ($n = 4$) dams of C57BL/6 mice. (**G**) DDX4 (green) staining in P3 ovaries of offspring from SPF and ABX dams in C57BL/6 mice. PI (red) was used to stain nuclei. Scale bars: 100 µm. (**H**) Comparison of the total number of germ cells per ovary in P3 ovaries from SPF ($n = 6$) and ABX ($n = 6$) dams of C57BL/6 mice. (**I**) Immunohistochemical staining of DDX4 in the ovaries of 6-week-old offspring from SPF and ABX dams of C57BL/6 mice. Representative images of ovarian follicles at different stages of development are displayed on the right panel. Scale bars: 200 µm. PmF primordial follicle, PF primary follicle, SF secondary follicle, AF antral follicle. (**J**) Quantification of the number of ovarian follicles at different stages of development in ovaries of 6-week-old offspring from SPF ($n = 7$) and ABX ($n = 7$) dams of C57BL/6 mice. Statistical significance was determined by one-way ANOVA followed by LSD multiple comparison test (**C**, **E**) and two-tailed unpaired *t*-test (**F**, **H**, **J**).

© The Author(s)

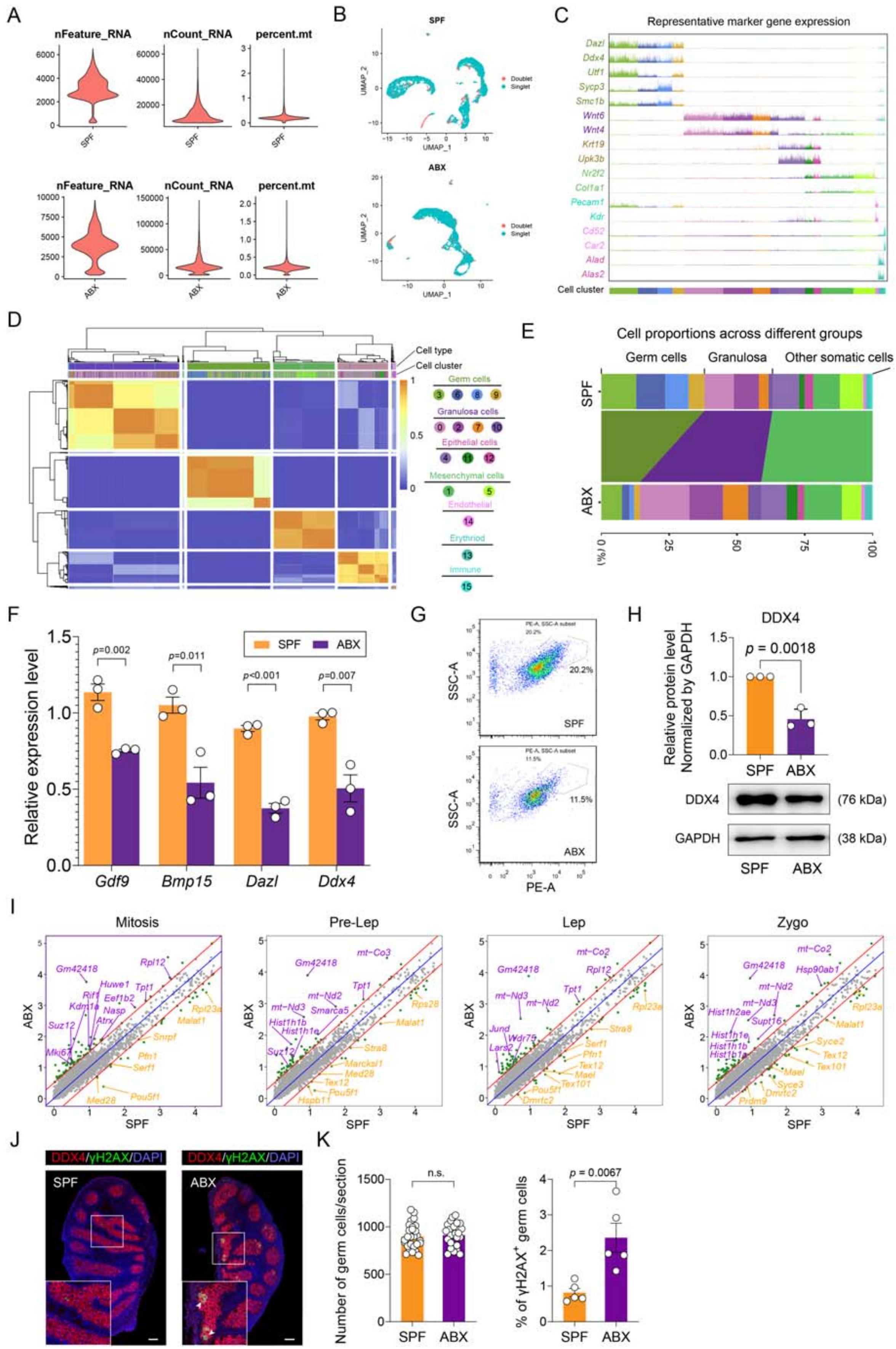
A
nFeature_RNA
nCount_RNA
percent.mt
SPF
ABX
B
SPF
ABX
UMAP_1
UMAP_2
Doublet
Singlet
C
Representative marker gene expression
Dazl
Ddx4
Utf1
Sycp3
Smc1b
Wnt6
Wnt4
Krt19
Upk3b
Nr2f2
Col1a1
Pecam1
Kdr
Cd52
Car2
Alad
Alas2
Cell cluster
D
Cell type
Cell cluster
Germ cells
Granulosa cells
Epithelial cells
Mesenchymal cells
Endothelial
Erythriod
Immune
E
Cell proportions across different groups
Germ cells
Granulosa
Other somatic cells
SPF
ABX
(%)
F
Relative expression level
SPF
ABX
p=0.002
p=0.011
p<0.001
p=0.007
Gdf9
Bmp15
Dazl
Ddx4
G
SSC-A
PE-A
20.2%
11.5%
SPF
ABX
H
DDX4
p = 0.0018
Relative protein level
Normalized by GAPDH
SPF
ABX
DDX4
(76 kDa)
GAPDH
(38 kDa)
SPF
ABX
I
Mitosis
Pre-Lep
Lep
Zygo
ABX
SPF
J
DDX4/γH2AX/DAPI
SPF
ABX
K
n.s.
Number of germ cells/section
SPF
ABX
p = 0.0067
% of γH2AX+ germ cells
SPF
ABX

Figure EV2. Quality control of scRNA seq datasets and validation of DDX4 expression in offspring ovaries from SPF and ABX dams.

(**A**) Comparison of the number of genes detected in each cell (nFeature_RNA), number of UMIs per cell (nCount_RNA), and the percentage of mitochondrial genes (percent.mt) of scRNA seq data from the SPF and ABX groups. (**B**) UMAP plot indicates potential doublets and singlets of scRNA seq data from the SPF and ABX groups. Doublet indicates two cells encapsulated into one reaction volume, while a singlet indicates one cell encapsulated. (**C**) Trackplot indicating representative cell marker gene expression in different cell clusters. (**D**) Consensus clustering of single-cell RNA-seq data from the SPF and ABX groups. (**E**) Comparison of cell cluster proportions between the SPF and ABX groups based on scRNA seq. (**F**) RT-qPCR comparison of representative germ cell marker gene expression between SPF ($n = 3$) and ABX ($n = 3$) fetal ovaries at E14.5. *Gapdh* was used as a loading control. Data were presented as mean ± SEM. (**G**) Flow cytometry quantification of the percentage of DDX4$^+$ germ cells between SPF and ABX fetal ovaries at E14.5. For each group, ovaries were collected from at least five independent fetuses. (**H**) Western blotting of germ cell marker DDX4 expression between the SPF ($n = 3$) and ABX ($n = 3$) groups. The expression level of DDX4 was normalized by GAPDH. (**I**) Comparative analysis and identifying germ cell-specific differentially expressed genes at various stages of meiosis between the SPF and ABX groups based on scRNA seq. (**J**) Representative immunofluorescence images showing DDX4 (red) and γH2AX (green) expression in E14.5 fetal testes from SPF and ABX dams. Scale bars, 50 μm. (**K**) Comparison of the total number of germ cells per section (left panel, $n = 27$, 26 for SPF and ABX, respectively) and the proportion of γH2AX-positive germ cells (right panel, $n = 5$, 5 for SPF and ABX, respectively) in E14.5 testes from SPF and ABX dams. Data were presented as mean ± SEM. Statistical significance was determined by a two-tailed unpaired *t*-test.

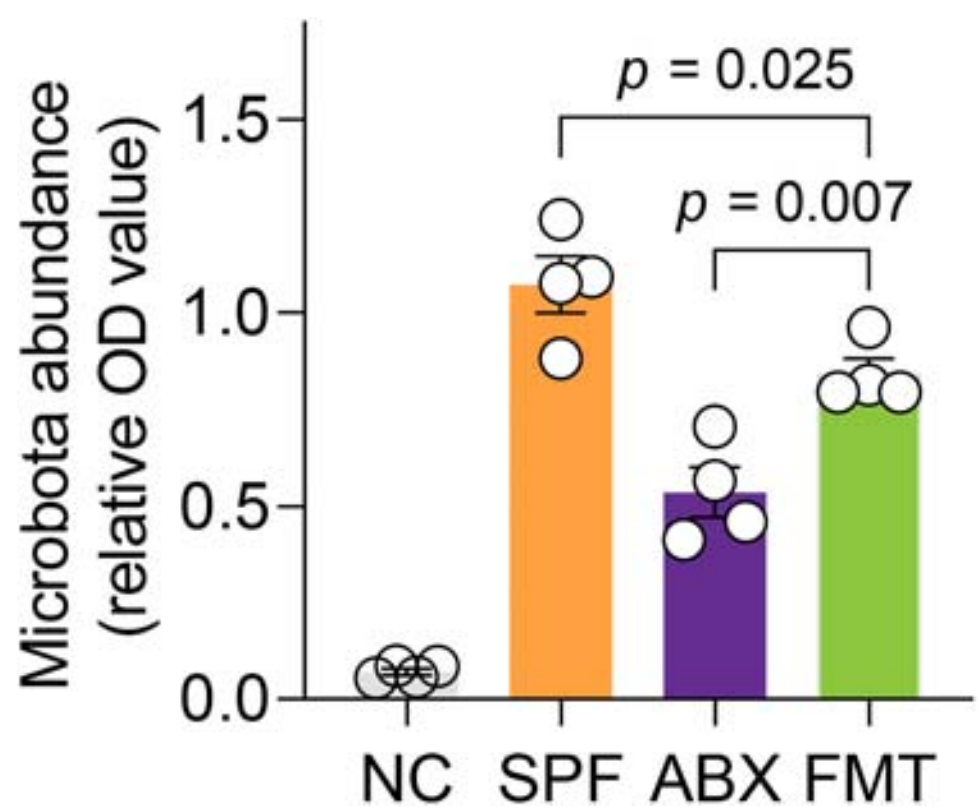

Figure EV3. Validation of FMT on rescuing intestinal microbiota abundance in CD1 mice.

Intestinal microbiota abundance in different groups analyzed by a spectrometer at 595 nm. Data were presented as mean ± SEM. Statistical significance was determined by one-way ANOVA followed by the LSD multiple comparison test.

 © The Author(s)

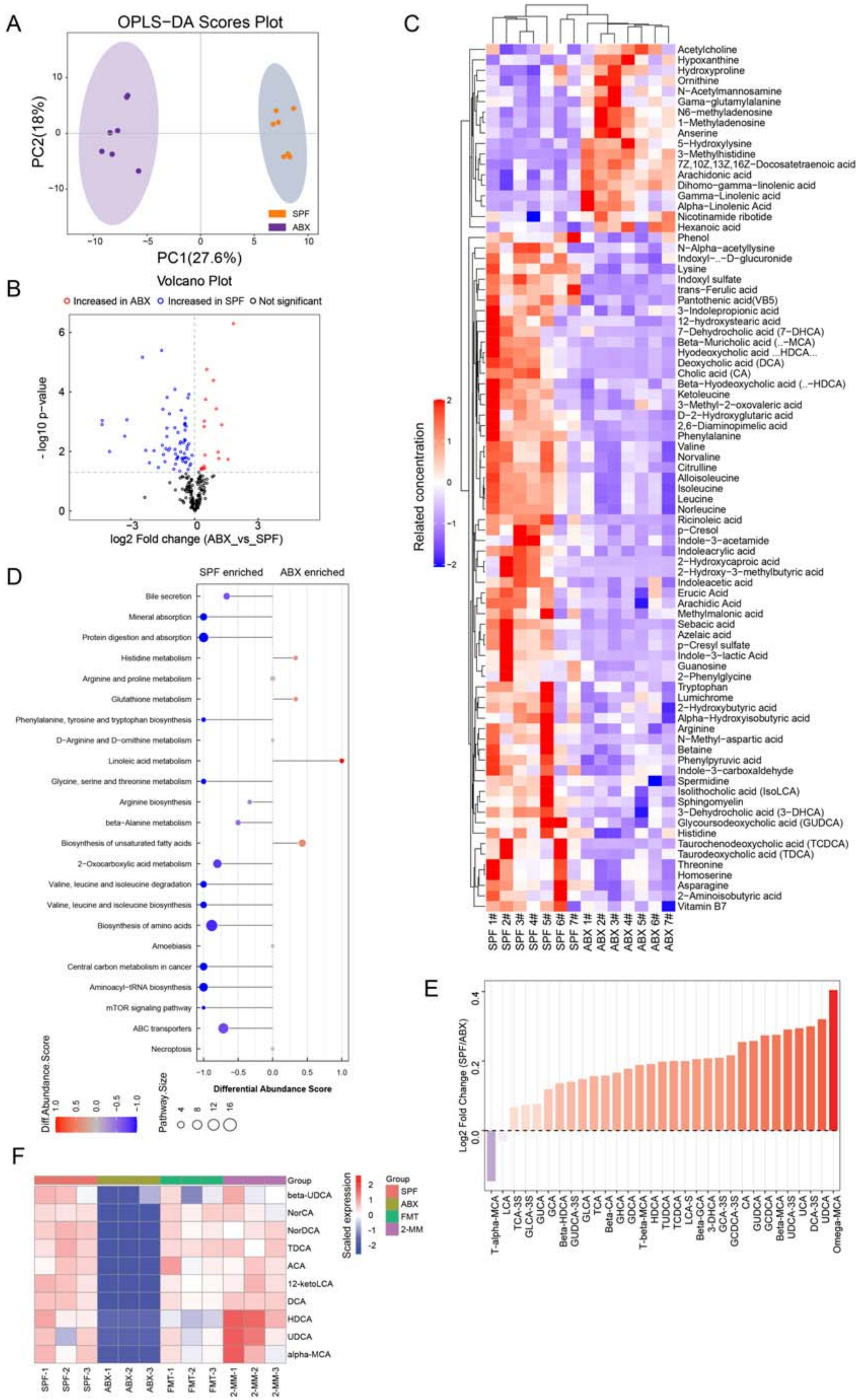
A
OPLS-DA Scores Plot
PC2(18%)
PC1(27.6%)
SPF
ABX
B
Volcano Plot
Increased in ABX
Increased in SPF
Not significant
- log10 p-value
log2 Fold change (ABX_vs_SPF)
C
Related concentration
Acetylcholine
Hypoxanthine
Hydroxyproline
Ornithine
N-Acetylmannosamine
Gama-glutamylalanine
N6-methyladenosine
1-Methyladenosine
Anserine
5-Hydroxylysine
3-Methylhistidine
7Z,10Z,13Z,16Z-Docosatetraenoic acid
Arachidonic acid
Dihomo-gamma-linolenic acid
Gamma-Linolenic acid
Alpha-Linolenic Acid
Nicotinamide ribotide
Hexanoic acid
Phenol
N-Alpha-acetyllysine
Indoxyl-..-D-glucuronide
Lysine
Indoxyl sulfate
trans-Ferulic acid
Pantothenic acid(VB5)
3-Indolepropionic acid
12-hydroxystearic acid
7-Dehydrocholic acid (7-DHCA)
Beta-Muricholic acid (..-MCA)
Hyodeoxycholic acid ...HDCA...
Deoxycholic acid (DCA)
Cholic acid (CA)
Beta-Hyodeoxycholic acid (..-HDCA)
Ketoleucine
3-Methyl-2-oxovaleric acid
D-2-Hydroxyglutaric acid
2,6-Diaminopimelic acid
Phenylalanine
Valine
Norvaline
Citrulline
Alloisoleucine
Isoleucine
Leucine
Norleucine
Ricinoleic acid
p-Cresol
Indole-3-acetamide
Indoleacrylic acid
2-Hydroxycaproic acid
2-Hydroxy-3-methylbutyric acid
Indoleacetic acid
Erucic Acid
Arachidic Acid
Methylmalonic acid
Sebacic acid
Azelaic acid
p-Cresyl sulfate
Indole-3-lactic Acid
Guanosine
2-Phenylglycine
Tryptophan
Lumichrome
2-Hydroxybutyric acid
Alpha-Hydroxyisobutyric acid
Arginine
N-Methyl-aspartic acid
Betaine
Phenylpyruvic acid
Indole-3-carboxaldehyde
Spermidine
Isolithocholic acid (IsoLCA)
Sphingomyelin
3-Dehydrocholic acid (3-DHCA)
Glycoursodeoxycholic acid (GUDCA)
Histidine
Taurochenodeoxycholic acid (TCDCA)
Taurodeoxycholic acid (TDCA)
Threonine
Homoserine
Asparagine
2-Aminoisobutyric acid
Vitamin B7
SPF 1# SPF 2# SPF 3# SPF 4# SPF 5# SPF 6# SPF 7# ABX 1# ABX 2# ABX 3# ABX 4# ABX 5# ABX 6# ABX 7#
D
SPF enriched
ABX enriched
Bile secretion
Mineral absorption
Protein digestion and absorption
Histidine metabolism
Arginine and proline metabolism
Glutathione metabolism
Phenylalanine, tyrosine and tryptophan biosynthesis
D-Arginine and D-ornithine metabolism
Linoleic acid metabolism
Glycine, serine and threonine metabolism
Arginine biosynthesis
beta-Alanine metabolism
Biosynthesis of unsaturated fatty acids
2-Oxocarboxylic acid metabolism
Valine, leucine and isoleucine degradation
Valine, leucine and isoleucine biosynthesis
Biosynthesis of amino acids
Amoebiasis
Central carbon metabolism in cancer
Aminoacyl-tRNA biosynthesis
mTOR signaling pathway
ABC transporters
Necroptosis
Differential Abundance Score
Diff.Abundance.Score
Pathway.Size
E
Log2 Fold Change (SPF/ABX)
T-alpha-MCA
LCA
TCA-3S
GLCA-3S
GUCA
GCA
Beta-HDCA
GUDCA-3S
GLCA
TCA
Beta-CA
GHCA
GDCA
T-beta-MCA
HDCA
TUDCA
TCDCA
LCA-S
Beta-GCA
3-DHCA
GCA-3S
GCDCA-3S
CA
GUDCA
GCDCA
Beta-MCA
UDCA-3S
UCA
DCA-3S
UDCA
Omega-MCA
F
Group
beta-UDCA
NorCA
NorDCA
TDCA
ACA
12-ketoLCA
DCA
HDCA
UDCA
alpha-MCA
Scaled expression
SPF
ABX
FMT
2-MM
SPF-1 SPF-2 SPF-3 ABX-1 ABX-2 ABX-3 FMT-1 FMT-2 FMT-3 2-MM-1 2-MM-2 2-MM-3

Figure EV4. Metabolomic profiling of E14.5 maternal serum in SPF and ABX dams.

(**A**) Orthogonal partial least squares discriminant analysis (OPLS-DA) on metabolomic profiles of maternal serum from SPF ($n = 7$) and ABX ($n = 7$) dams at E14.5. For each group, serum samples were collected from seven different dams. (**B**) Volcano plot illustrating differentially accumulated metabolites in maternal serum of SPF ($n = 7$) and ABX ($n = 7$) dams. (**C**) Heatmap illustrating the relative expression level of identified differentially accumulated metabolites between maternal serum from SPF and ABX dams. (**D**) Differential abundance (DA) score analysis of KEGG-enriched pathways in maternal serum from SPF and ABX dams. (**E**) Bar chart illustrating the Log2 fold change (SPF/ABX) of 31 detected bile acids in fetal ovaries from SPF and ABX dams at E14.5. For each group, at least 30 ovaries were included for analysis, and the final concentrations were normalized to cell number. (**F**) Heatmap illustrating the scaled abundance levels of ten representative bile acids in the maternal serum of SPF, ABX, FMT, and 2-MM dams. Each group includes three replicates, with each replicate representing a serum sample from a different dam. Statistical significance was determined by a two-tailed unpaired *t*-test (**B**).

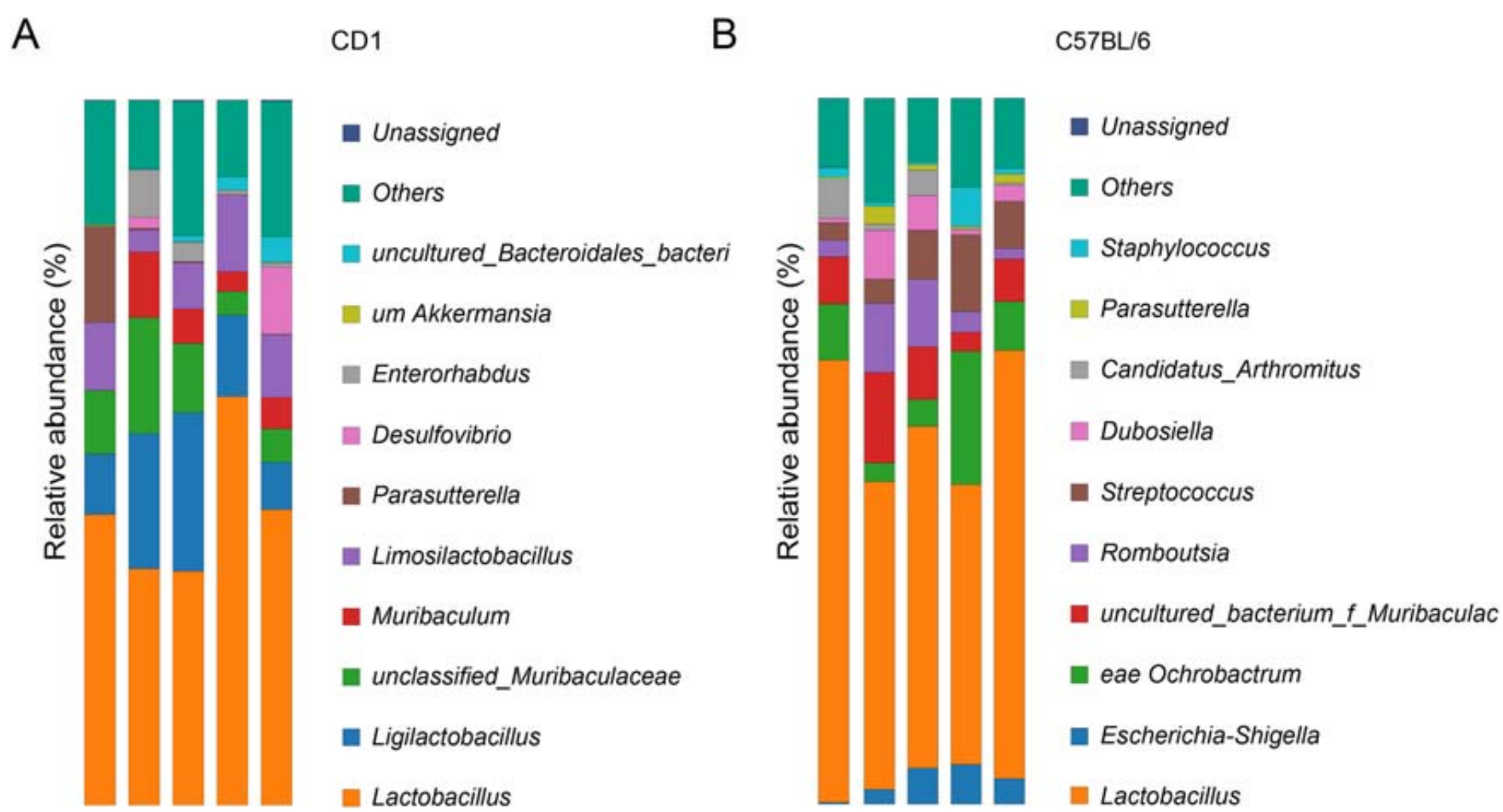

Figure EV5. Comparison of microbiota conservation between CD1 and C57 mice.

(**A**) Genus-level taxonomic diversity of maternal fecal microbiota collected from SPF mice of the CD1 strain. (**B**) Genus-level taxonomic diversity of maternal fecal microbiota collected from SPF mice of the C57BL/6 strain.

 © The Author(s)

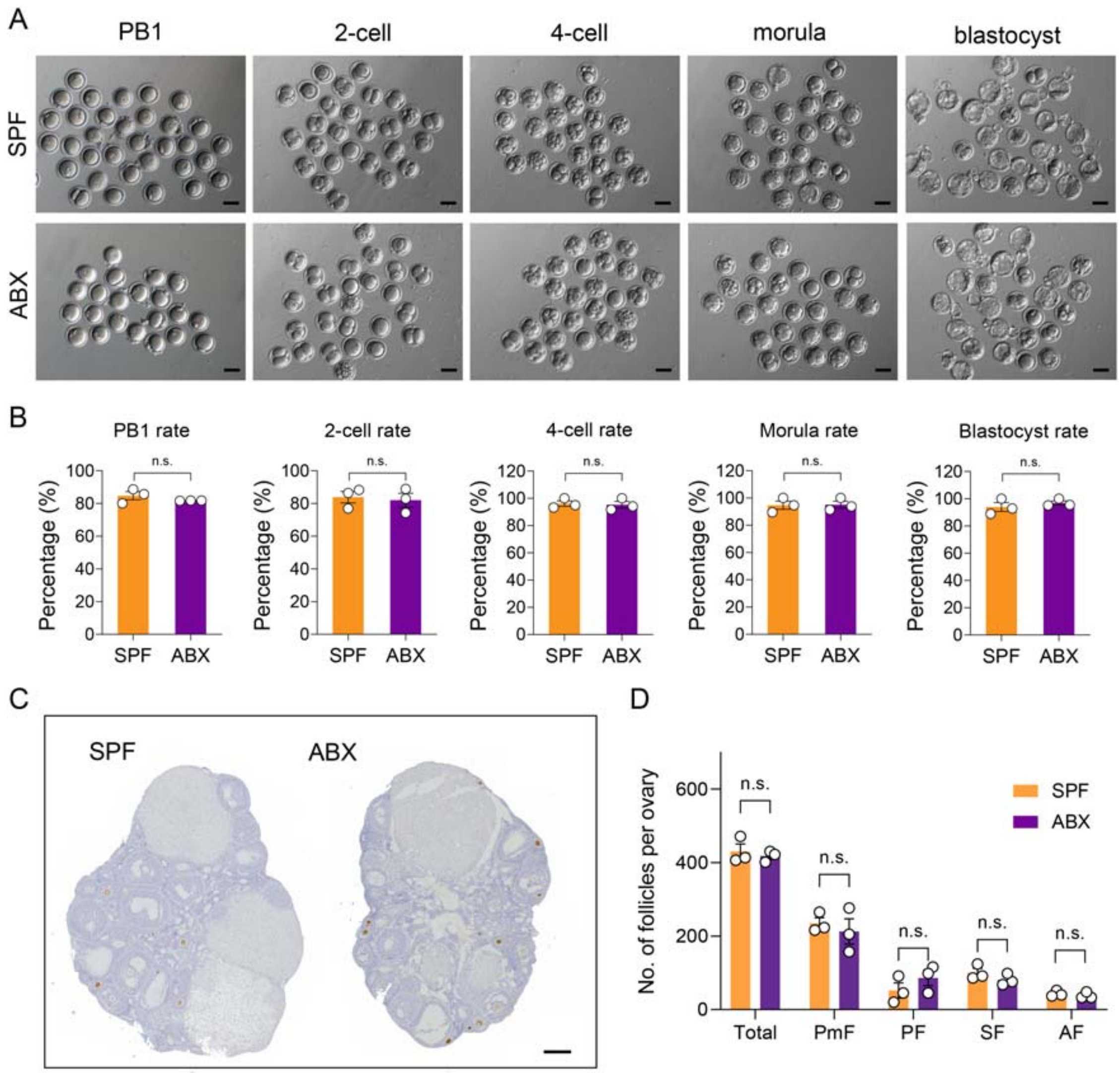

Figure EV6. Comparison of the developmental ability of oocytes from SPF and ABX dams.

(**A**) Representative images of PB1 and early embryos using oocytes collected from SPF and ABX mice. Scale bars: 100 µm. (**B**) Comparative analysis of PB1, 2-cell, 4-cell, morula, and blastocyst rates using oocytes collected from SPF and ABX dams. At least three repeats were included for each group. (**C**) Immunohistochemical staining of DDX4 in the ovaries of SPF and ABX dams. Ovarian tissues were collected from the dams of the adult offspring. Scale bars: 200 µm. (**D**) Quantification of the total number of ovarian follicles and the number of ovarian follicles at different stages of development in the ovaries of SPF ($n = 3$) and ABX ($n = 3$) dams. Data points in (**B**) represent different mice. Data are presented as mean ± SEM. Statistical significance was determined by a two-tailed unpaired t-test.
