# Supplementary material for: Maternal microbiome promotes offspring ovarian reserve via bile acid metabolism
Source: EMBO Rep. 2026 Aug 27;27(20):5752–78. doi: 10.1038/s44319-026-00912-3 (PMC13694719; doi:10.1038/s44319-026-00912-3)
Supplement: Supplementary file 2 [file 44319_2026_912_MOESM5_ESM.pdf]

EMBO reports

Maternal microbiome promotes offspring ovarian reserve via bile acid metabolism

Han Wang, Yu-Kang Li, Yilin Niu, Xin Chen, Jing Liu, Weiting Feng, Jingru Cao, Haixia Liu, Chang Liu, Xiaoying Liang, Ziqing Ge, Jun-Jie Wang, Xinlei Feng, Shun-Feng Cheng, Wei Shen, and Wei Ge

Corresponding author(s): Wei Ge (gewei0901@qau.edu.cn) , Wei Shen (wshen@qau.edu.cn)

Review Timeline:

Submission Date:	23rd Jan 26
Editorial Decision:	5th Mar 26
Revision Received:	9th Jun 26
Editorial Decision:	28th Jul 26
Revision Received:	8th Aug 26
Accepted:	13th Aug 26

Editor: Achim Breiling

Transaction Report:

(Note: With the exception of the correction of typographical or spelling errors that could be a source of ambiguity, letters and reports are not edited. Depending on transfer agreements, referee reports obtained elsewhere may or may not be included in this compilation. Referee reports are anonymous unless the Referee chooses to sign their reports.)

1st Editorial Decision **5th Mar 2026**

Dear Dr. Ge,

Thank you for the submission of your manuscript to EMBO reports that has been previously reviewed at another venue. I have now received report from the arbitrator I asked to evaluate your study, which can be found at the end of this email. As you will see, the arbitrator feels that the experiments and results are convincing and have far-reaching importance, but s/he has several comments, concerns, and suggestions to improve the manuscript, indicating that a further revision of the manuscript is necessary to allow publication of the study in EMBO reports.

Given these constructive comments, I would like to invite you to further revise your manuscript with the understanding that the concerns of the referees must be addressed in the revised manuscript and/or in a detailed further point-by-point response. Acceptance of your manuscript will depend on a positive outcome of a final round of review, using the same arbitrator.

Revised manuscripts should be submitted within three months of a request for revision. Please contact me to discuss the revision (also by video chat) if you have questions or comments, or should you need additional time.

Moreover, your manuscript needs formatting to our journal style. Please also carefully review the instructions that follow below.

PLEASE NOTE THAT upon resubmission revised manuscripts are subjected to an initial quality control prior to exposition to re-review. Upon failure in the initial quality control, the manuscripts are sent back to the authors, which may lead to delays. Frequent reasons for such a failure are the lack of the data availability section (please see below) and the presence of statistics based on n=2 (the authors are then asked to present scatter plots or provide more data points).

When submitting your revised manuscript, we will require:

1) a .docx formatted version of the final manuscript text (including legends for main figures, EV figures and tables), but without the figures included. Figure legends should be compiled at the end of the manuscript text.

2) individual production quality figure files as .eps, .tif, .jpg (one file per figure), of main figures and EV figures. Please upload these as separate, individual files upon re-submission.

The Expanded View format, which will be displayed in the main HTML of the paper in a collapsible format, has replaced the Supplementary information. You can submit up to 6 images as Expanded View. Please follow the nomenclature Figure EV1, Figure EV2 etc. The figure legend for these should be included in the main manuscript document file in a section called Expanded View Figure Legends after the main Figure Legends section. Additional Supplementary material should be supplied as a single pdf file labeled Appendix. The Appendix should have page numbers and needs to include a table of content on the first page (with page numbers) and legends for all content. Please follow the nomenclature Appendix Figure Sx, Appendix Table Sx etc. throughout the text, and also label the figures and tables according to this nomenclature.

For more details, please refer to our guide to authors:
https://link.springer.com/journal/44319/submission-guidelines#cms-Revised-submissions

Please consult our guide for figure preparation:
https://media.springernature.com/original/springer-cms/rest/v1/content/27825798/data/v1

3) a .docx formatted letter INCLUDING the reviewers' reports and your detailed point-by-point responses to their comments. As part of the EMBO Press transparent editorial process, the point-by-point response is part of the Review Process File (RPF), which will be published alongside your paper.

4) a complete author checklist, which you can download from our author guidelines (https://link.springer.com/journal/44319/submission-guidelines#cms-Revised-submissions). Please insert page numbers in the checklist to indicate where the requested information can be found in the manuscript. The completed author checklist will also be part of the RPF.

Please also follow our guidelines for the use of living organisms, and the respective reporting guidelines:
https://link.springer.com/partners/embo-press/editorial-policies#Animal%20and%20human%20studies

5) that primary datasets produced in this study (e.g. RNA-seq, ChIP-seq, structural and array data) are deposited in an appropriate public database. If no primary datasets have been deposited, please also state this in a dedicated section (e.g. 'No primary datasets have been generated and deposited'), see below.

See also: https://link.springer.com/partners/embo-press/editorial-policies#Data%20deposition

Please remember to provide a reviewer password if the datasets are not yet public.

The accession numbers and database should be listed in a formal "Data Availability" section that follows the model below. This is now mandatory (like the COI statement). Please note that the Data Availability Section is restricted to new primary data that are part of this study. This section is mandatory. As indicated above, if no primary datasets have been deposited, please state this in this section

Data availability

The datasets produced in this study are available in the following databases:

- RNA-Seq data: Gene Expression Omnibus GSE46843 (https://www.ncbi.nlm.nih.gov/geo/query/acc.cgi?acc=GSE46843)
- [data type]: [name of the resource] [accession number/identifier/doi] ([URL or identifiers.org/DATABASE:ACCESSION])

*** Note - All links should resolve to a page where the data can be accessed. ***

See also: https://link.springer.com/partners/embo-press/editorial-policies#Data%20availability%20statement

Moreover, I have these editorial requests:

6) We now request the publication of original source data with the aim of making primary data more accessible and transparent to the reader. You will receive a separate email with instructions for providing source data with your revised manuscript, including information how to upload and organize the files.

7) Our journal encourages inclusion of *data citations in the reference list* to directly cite datasets that were re-used and obtained from public databases. Data citations in the article text are distinct from normal bibliographical citations and should directly link to the database records from which the data can be accessed. In the main text, data citations are formatted as follows: "Data ref: Smith et al, 2001" or "Data ref: NCBI Sequence Read Archive PRJNA342805, 2017". In the Reference list, data citations must be labeled with "[DATASET]". A data reference must provide the database name, accession number/identifiers and a resolvable link to the landing page from which the data can be accessed at the end of the reference. Further instructions are available at: https://link.springer.com/journal/44319/submission-guidelines#cms-Reference-guidelines

8) Regarding data quantification and statistics, please make sure that the number "n" for how many independent experiments were performed, their nature (biological versus technical replicates), the bars and error bars (e.g. SEM, SD) and the test used to calculate p-values is indicated in the respective figure legends (also for EV and Appendix figures). Please also check that all the p-values are explained in the legend, and that these fit to those shown in the figure. Please provide statistical testing where applicable. Please avoid the phrase 'independent experiment' but clearly state if these were biological or technical replicates. Please also indicate (e.g. with n.s.) if testing was performed, but the differences are not significant. In case n=2, please show the data as separate datapoints without error bars and statistics. See also: https://link.springer.com/journal/44319/submission-guidelines#cms-Figure-and-data-presentation

If n<5, please show single datapoints for diagrams.

9) Please add scale bars of similar style and thickness to microscopic images, using clearly visible black or white bars (depending on the background). Please place these in the lower right corner of the images themselves. Please do NOT write on or near the bars in the image but define the size in the respective figure legend.

10) Please also note our reference format: https://link.springer.com/journal/44319/submission-guidelines#cms-Reference-guidelines

11) We updated our journal's competing interests policy in January 2022 and request authors to consider both actual and perceived competing interests. Please name this section 'Disclosure and Competing Interests Statement' and put it after the Acknowledgements section.
See: https://link.springer.com/partners/embo-press/editorial-policies#Competing%20interest%20disclosures

12) We now use CRediT to specify the contributions of each author in the journal submission system. CRediT replaces the author contribution section. Please use the free text box to provide more detailed descriptions and do NOT provide your final manuscript text file with an author contributions section. See also our guide to authors (section 'Author contributions'): https://link.springer.com/journal/44319/submission-guidelines#cms-Revised-submissions

13) All Materials and Methods need to be described in the main text using our 'Structured Methods' format, which is required for all research articles. According to this format, the Methods section should include a Reagents and Tools Table (listing key reagents, experimental models, software, and relevant equipment and including their sources and relevant identifiers), uploaded as separate file, and a Methods section in which we encourage the authors to describe their methods using a step-by-step

protocol format with bullet points, to facilitate the adoption of the methodologies across labs. More information on how to adhere to this format as well as downloadable templates (.doc) for the Reagents and Tools Table can be found in our author guidelines (section 'Structured Methods'):

https://link.springer.com/journal/44319/submission-guidelines#cms-Manuscript-organisation-and-formatting

Please add all primer or antibody information directly to the Reagents and Tools table.

14) Please add up to five keywords and order the manuscript sections like this, using only these names:
Title page - Abstract (max. 175 words) - Keywords (max. 5) - Introduction - Results - Discussion - Methods - Data availability section - Acknowledgements (please include here all the funding information) - Disclosure and Competing Interests Statement - References - Figure legends - Expanded View Figure legends

15) Please make sure that all the funding information is also entered into the online submission system and that it is complete and similar to the one in the acknowledgement section of the manuscript text file.

We would also welcome the submission of cover suggestions, or motifs to be used by our Graphics Illustrator in designing a cover.

As part of the EMBO publication's Transparent Editorial Process, EMBO reports publishes online a Review Process File (RPF) to accompany accepted manuscripts. This File will be published in conjunction with your paper and will include the referee reports, your point-by-point response and all pertinent correspondence relating to the manuscript.

You are able to opt out of this by letting the editorial office know (emboreports@embo.org). If you do opt out, the Review Process File link will point to the following statement: "No Review Process File is available with this article, as the authors have chosen not to make the review process public in this case."

I look forward to seeing a revised form of your manuscript when it is ready.

Please use this link to submit your revision: https://embor.msubmit.net/cgi-bin/main.plex

Yours sincerely,

Achim Breiling
Senior Editor
EMBO Reports

Referee #1:

The authors explore how antibiotic-induced maternal microbiome perturbation leads to diminished ovarian reserve (DOR) in F1 offspring, and explore ways to reverse this outcome, using FMT, probiotic L gasseri, or supplementation of specific bile acid metabolites. They formulate their hypothesis based on previous work by Vuong et al 2020, Huang et al 2024, and Qi et al 2019: on maternal gut microbiota-fetal and gut microbiota-ovary interactions. Authors conducted several lines of experiments for testing and validating their hypothesis using mouse models and applied scRNAseq, 16S, DDX4 IF, DNA damage marker γH2AX and LC-MS assays. The authors demonstrated ABX-induced dysbiosis impaired germline development around mid-gestation and caused DNA damage, resulting in DOR and compromised oocyte quality in F1 offspring. Mechnistically, ABX-dysbiosis, which results in a deficiency of TDCA, GUDCA and other bile acids, causes a high rate of meiotic defects (ie. DNA damage) during germline development, which can be rescued by gavaging FMT or L gasseri before conception or by supplementing with TDCA and GUDCA between E6.5-E16.5 in ABX mice. Overall, the experiments and the results are convincing and have far-reaching importance for women's reproductive health care, however, the mechanisms described and the conclusions drawn still need additional lines of experimental evidence.

The manuscript needs to address the following major remarks:

1. IF and scRNAseq are not sufficient to reach this conclusion, since both methods are prone to subjective interpretation and technical differences in GEM generation using 10X kit, respectively. This finding needs to be validated by using FACS to demonstrate a reduction in germ cell numbers in gonads between ABX vs SPF, as well as qPCR to demonstrate a reduction in germline marker genes such as Gdf9, ZP, Dppa3, and Bmp15...etc. These additional data are needed to make a definitive conclusion.

2. Authors conclude that the DOR phenotype observed in the F1 offspring is mainly caused by antibiotic-induced maternal microbiome disruption rather than decreased F0 maternal oocyte quality. Hence, this is not a F0 germline mediated effect, but rather a dysbiosis-induced in utero effect that occurs from E6.5 to E16.5 during primordial germ cell (PGC) development. As this

window period applies to both male and female gonads (PGC) development, it is expected that the F1 offspring germline effect (DNA damage) can also manifest in the P3 testis of males or E14.5 gonads. This way, both phenotype and in utero effects can be conclusive, otherwise showing only female germline effects is not convincing. Regardless of the results, data on male gonadal development should be included in the manuscript.

3. Authors didn't report fetal gonadal bile acid levels, raising the possibility that this adverse outcome could be the result of stress or hypoxia rather than a deficiency in bile acid metabolites that protect the germline. The recommended method is to use C13-labeled TDCA and GUDCA metabolites tracers to map the mechanisms that link these specific bile acids to specific outcomes; if the authors cant' use C13-labeled metabolites, they must at least quantify ABX vs SPF bile acid levels in the fetal gonads. Especially since they didn't show how the lack of these metabolites (bile acid) causes DNA damage, measuring bile acid levels in gonads would give more confidence that the DOR is at least partially bile acid-mediated, showing only maternal data is insufficient for conclusion the authors reached. Please also note that after Abx termination, there is at least 2-week recovery period from E0.5-E14.5. Previous studies have shown that TDCA and GUDCA are either stable or recover relatively quickly in 1 wk - please see Fig8 and Fig9 of this article: Choucair I et al, 2020: https://www.sciencedirect.com/science/article/pii/S0022227520435345#cesec10

Minor concerns:

Fig 1A, it seems that ABX is administered in utero between E6.5-E16.5, I find this confusing. If they treat the mice with ABX during this period, authors should clearly discuss this in the manuscript and method section as well. If this is not clarified well, the use of other non-absorable ABX might be recommended for this manuscript to exclude metronidazole's direct genotoxic effects. It is also important to note that ABX residues can also be found in mouse feces after ABX termination, causing mice to continue to be exposed to ABX after mating, unless the cage is changed with a new bedding. It would be useful if you could clarify this in the method section and remove the confustion from Fig 1A.

Based on the microbiome abundance levels shown in supp Fig 1A-C, it is not clear what time point they represent. Authors should include abundance data prior to ABX, right after ABX termination, and at E14.5 recovery. If the mice are kept in the same cage, diet and environment, their gut microbiome usually recovers faster, so it is important to show the relative abundance before and after ABX termination.

Why are only germline cells susceptible to DNA damage? How about the surrounding somatic cells? What protects somatic cells? Fig 2H showing γH2AX co-localized with DDX4 signals, indicating germ cell populations - we can't distinguish somatic from germline cells at this resolution, more clear magnification is needed.

In Figure 6A there are some images that don't match with authors claim, especially if you count from 2-cells to blastocyts, it lacks consistency in numbers e.g. FMT, on top of this the FMT blastocyts image looks more affected than the ABX blastocyt images. IVF experiments are quite noisy, if they are to be convincing, they need multiple experiments, and qualitative data taken from different IVF experiments rather than the same IVF experiment makes the data less convincing. Authors are strongly advised to put clear, nice and quantitatively consistent number of embroys figures to minimize reader confusion.

Line 400 " While our findings here support the conclusion that periconceptional antibiotic-mediated disruption of the maternal microbiome induces diminished ovarian reserve in offspring". This conclusion is hardly true for two reasons: First, matured oocytes are not affected in F0 mothers, and second, the dysbiotc condition remains even after mating, thus E14.5 metabolomics profile reveals a difference and E6.5-E16.5 supplements restored the phenotype. To conclude this is a periconceptional effect, either the antibiotic-mediated disruption must be fully recovered before mating, or one needs to perform IVF and transfer the 2-cell embryos to a pseudopregnant CD1 foster mother that are not exposed to ABX, followed by a gonads (germline) check at E14.5 and P3. In any case, if the dysbiosis in pregnant F0 dams is not completely recovered during pregnancy, the effects could be additive during and after pregnancy, but the evidence presented in this manuscript (F1 germline) supports that this effect is more likely to occur due to prolonged in-utero dysbiosis than periconceptional dysbiosis alone. In the manuscript, the author should remove the word "periconceptional line 348/400" and replace it with "maternal gut dysbiosis" or "pervasive maternal gut perturbations".

Point-by-point response

Title: Maternal microbiome promotes offspring ovarian reserve via bile acid metabolism

No: EMBOR-2026-63683V2

EMBO reports

First, we would like to thank the editors and the reviewer very much for their helpful comments.

Referee #1:

The authors explore how antibiotic-induced maternal microbiome perturbation leads to diminished ovarian reserve (DOR) in F1 offspring, and explore ways to reverse this outcome, using FMT, probiotic L gasseri, or supplementation of specific bile acid metabolites. They formulate their hypothesis based on previous work by Vuong et al 2020, Huang et al 2024, and Qi et al 2019: on maternal gut microbiota-fetal and gut microbiota-ovary interactions. Authors conducted several lines of experiments for testing and validating their hypothesis using mouse models and applied scRNAseq, 16S, DDX4 IF, DNA damage marker γH2AX and LC-MS assays. The authors demonstrated ABX-induced dysbiosis impaired germline development around mid-gestation and caused DNA damage, resulting in DOR and compromised oocyte quality in F1 offspring. Mechnistically, ABX-dysbiosis, which results in a deficiency of TDCA, GUDCA and other bile acids, causes a high rate of meiotic defects (ie. DNA damage) during germline development, which can be rescued by gavaging FMT or L gasseri before conception or by supplementing with TDCA and GUDCA between E6.5-E16.5 in ABX mice. Overall, the experiments and the results are convincing and have far-reaching importance for women's reproductive health care, however, the mechanisms described and the conclusions drawn still need additional lines of experimental evidence.

Response: We thank the reviewer for the very positive evaluation of our work.

<u>Major concerns:</u>

1. IF and scRNAseq are not sufficient to reach this conclusion, since both methods are prone to subjective interpretation and technical differences in GEM generation using 10X kit, respectively. This finding needs to be validated by using FACS to demonstrate a reduction in germ cell numbers in gonads between ABX vs SPF, as well as qPCR to demonstrate a reduction in germline marker genes such as Gdf9, ZP, Dppa3, and Bmp15...etc. These additional data are needed to make a definitive conclusion.

Response: We thank the reviewer for this critical and constructive comment. We fully agree that extra validation beyond IF and scRNA-seq is essential to substantiate our conclusion. In our revised manuscript, we performed RT-qPCR and flow cytometry analysis to further support our observations.

Specifically, RT-qPCR analysis revealed that the mRNA expression levels of germ cell marker genes *Gdf9*, *Bmp15*, *Dazl*, and *Ddx4* were significantly decreased in the E14.5 ovary of ABX offspring (**Figure EV2F**), consistent with our previous quantification analysis using IF and scRNAseq.

Furthermore, to quantify the percentage of DDX4^{+} germ cells between SPF and ABX fetal ovaries at E14.5 at the protein level, we performed flow cytometry analysis using dissociated fetal ovarian cells from both SPF and ABX groups at E14.5. Specifically, to ensure the accuracy of our analysis, for each group, a minimum of 10 ovaries from 5 independent donors were collected for flow cytometry analysis. The results also showed a notable reduction of the DDX4^{+} germ cells in the ABX group (11.5%) compared to the SPF group (20.2%) (**Figure EV2G**). Collectively, both gene expression level analysis and protein level analysis support our conclusion that disruption of the maternal microbiome impairs fetal germ cell development in offspring. All the updated figures have now been supplemented in our revised manuscript.

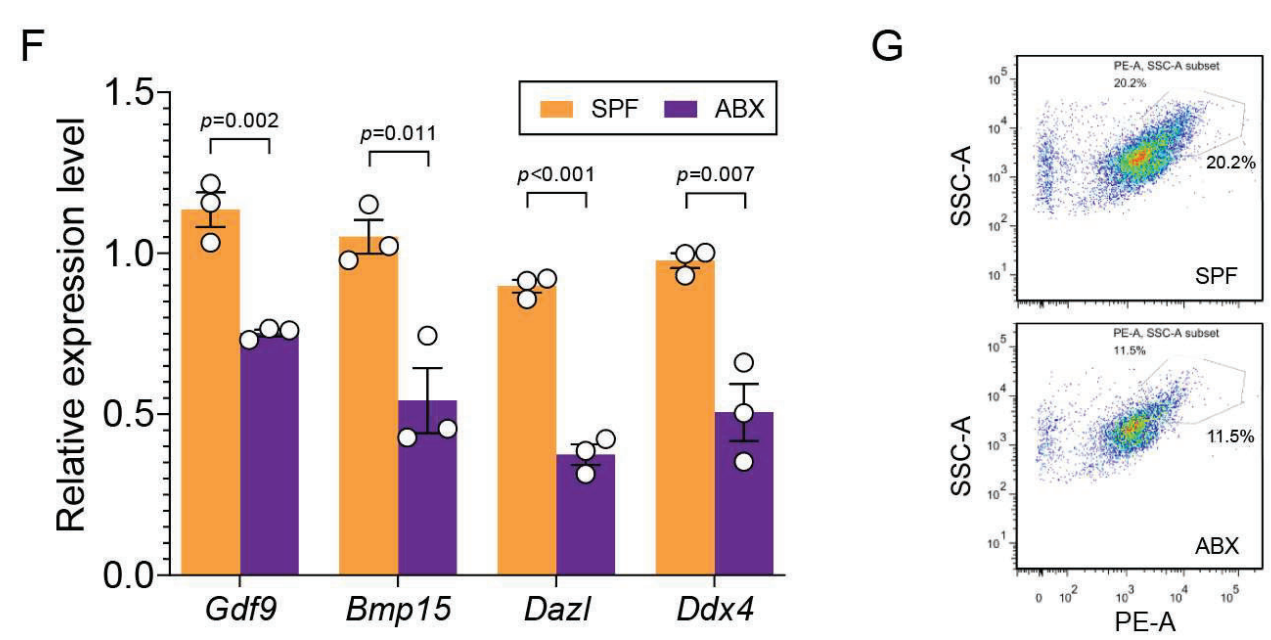

Appendix Figure EV2: *(F) RT-qPCR comparison of representative germ cell marker gene expression between SPF and ABX fetal ovaries at E14.5. Gapdh was used as a loading control. Statistics in the barplot are displayed as Mean ± SEM. (G) Flow cytometry quantification of the percentage of DDX4^{+} germ cells between SPF and ABX fetal ovaries at E14.5. For each group, ovaries were collected from at least five independent fetuses.*

2. Authors conclude that the DOR phenotype observed in the F1 offspring is mainly caused by antibiotic-induced maternal microbiome disruption rather than decreased F0 maternal oocyte quality. Hence, this is not a F0 germline mediated effect, but rather a dysbiosis-induced in utero effect that occurs from E6.5 to E16.5 during primordial germ cell (PGC) development. As this window period applies to both male and female gonads (PGC) development, it is expected that the F1 offspring germline effect (DNA damage) can also manifest in the P3 testis of males or E14.5 gonads. This way, both phenotype

and in utero effects can be conclusive, otherwise showing only female germline effects is not convincing. Regardless of the results, data on male gonadal development should be included in the manuscript.

Response: We thank the reviewer for this suggestion. As the reviewer suggested, in our revised manuscript, we used DDX4 and γH2AX staining to quantify the number of germ cells and germ cell damage between SPF and ABX fetal testes at E14.5 (**Figure EV2J,K**). The results showed that the number of DDX4 germ cells between SPF and ABX fetal testes at E14.5 showed no differences between SPF and ABX fetal testes at E14.5. However, we observed an increased number of γH2AX-positive germ cells in the ABX fetal testes at E14.5, even though the percentage is relatively low (about 2.4%). Together, both female and male data emphasize the importance of the maternal microbiome on germ cell development in the offspring.

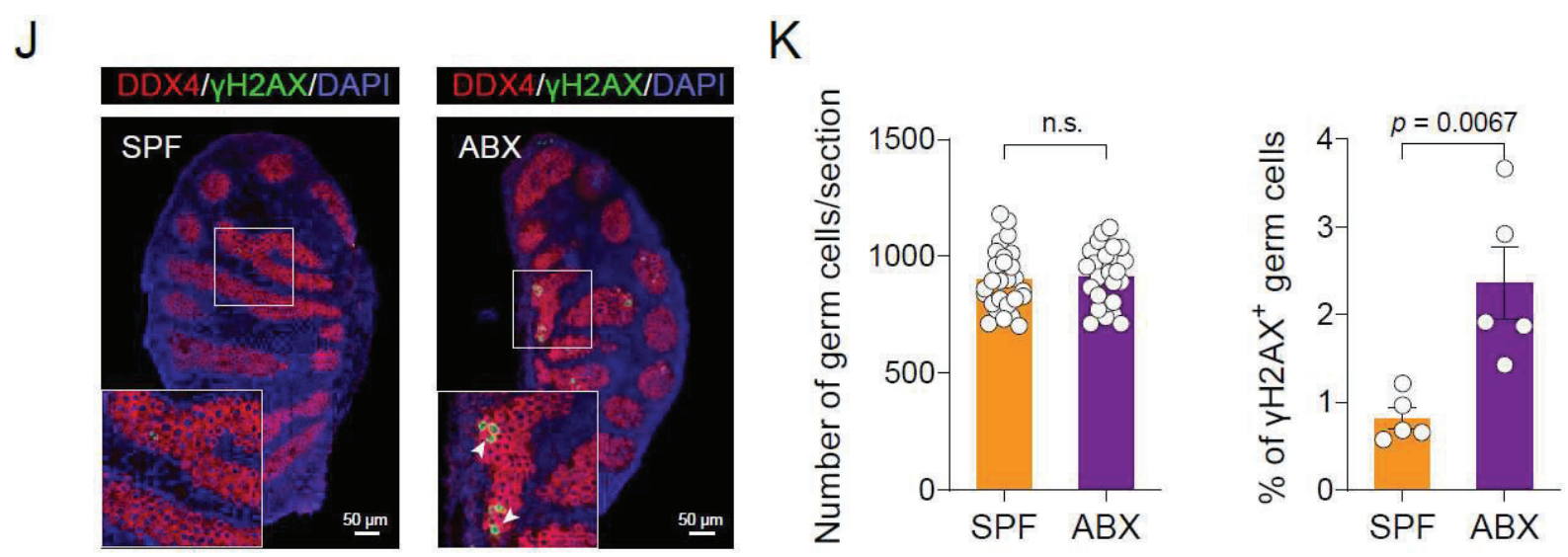

Appendix Figure EV2: *(J) Representative immunofluorescence images showing DDX4 (red) and γH2AX (green) expression in E14.5 fetal testes from SPF and ABX dams. Scale bars, 50 μm. (K) Comparison of the total number of germ cells per section and the proportion of γH2AX positive germ cells in E14.5 testes from SPF and ABX dams.*

Line 180-189: *In contrast, co-staining of DDX4 and γH2AX in E14.5 fetal testis revealed that the percentage of γH2AX positive germ cells was significantly increased in the fetuses of ABX dams, while the total number of germ cells was comparable between SPF and ABX groups. Notably, the magnitude of the increase in γH2AX-positive germ cells following maternal microbiome disruption was substantially lower in fetal testes than in fetal ovaries (~2.5% vs ~60%). These findings therefore suggest that maternal microbiome disruption may exert a sex-biased effect on fetal germ cell development.*

Specifically, when comparing the percentage of germ cell damage between female and male data, it is obvious that female germ cells were more vulnerable to antibiotic-induced maternal microbiome disruption. This is possible since the fact that female germ cells initiate the meiotic program at E13.5, during which SPO11 induces large-scale programmed DNA double-strand breaks (DSBs) to promote homologous chromosome pairing and crossover formation in mice (1), while for male germ cells, germ cells in the fetal testis do not enter meiotic program at E13.5, but undergo mitotic arrest at the G0/G1 phase, which persists until birth. For the progression of meiosis, it is not until postnatal days 8 to 10 that male spermatogonia initiate the meiotic program. It is therefore plausible that sexual developmental asynchrony in meiotic progression

may account for the different effects of antibiotic-induced maternal microbiome disruption in female and male offspring. Moreover, it's worth noting that substantial studies have demonstrated that female germ cells en route to the meiotic program in utero are vulnerable to environmental perturbations, such as chemotherapeutic drugs, endocrine-disrupting chemicals, and irradiation, which similarly lead to ovarian reserve depletion (3). Collectively, it is reasonable that antibiotic-induced maternal microbiome disruption may induce more prominent impacts on the germ cell development of female offspring.

In our revised manuscript, we have supplemented related information accordingly, and we believe that the updated contents have enhanced the manuscript quality as suggested by the reviewer.

Reference:

1. *Shin Y H, McGuire M M, Rajkovic A. Mouse HORMAD1 is a meiosis checkpoint protein that modulates DNA double-strand break repair during female meiosis[J]. Biology of reproduction, 2013, 89(2): 29, 1-12.*
2. *Yoshida S, Sukeno M, Nakagawa T, Ohbo K, Nagamatsu G, Suda T, Nabeshima Y. The first round of mouse spermatogenesis is a distinctive program that lacks the self-renewing spermatogonia stage. Development. 2006 Apr;133(8):1495-505.*
3. *Richardson MC, Guo M, Fauser BC, Macklon NS. Environmental and developmental origins of ovarian reserve[J]. Hum Reprod Update, 2014, May-Jun;20(3):353-69.*

3. Authors didn't report fetal gonadal bile acid levels, raising the possibility that this adverse outcome could be the result of stress or hypoxia rather than a deficiency in bile acid metabolites that protect the germline. The recommended method is to use C13-labeled TDCA and GUDCA metabolites tracers to map the mechanisms that link these specific bile acids to specific outcomes; if the authors cant' use C13-labeled metabolites, they must at least quantify ABX vs SPF bile acid levels in the fetal gonads. Especially since they didn't show how the lack of these metabolites (bile acid) causes DNA damage, measuring bile acid levels in gonads would give more confidence that the DOR is at least partially bile acid-mediated, showing only maternal data is insufficient for conclusion the authors reached. Please also note that after Abx termination, there is at least 2-week recovery period from E0.5-E14.5. Previous studies have shown that TDCA and GUDCA are either stable or recover relatively quickly in 1 wk - please see Fig8 and Fig9 of this article: Choucair I et al, 2020: https://www.sciencedirect.com/science/article/pii/S0022227520435345#cesec10

Response: We thank the Reviewer for raising this important and insightful question. We fully agree with the reviewer that quantifying ABX vs SPF bile acid levels in the fetal gonads is essential to strengthen our conclusions. Therefore, in our revised manuscript, we used targeted metabolomics to evaluate representative bile acid levels in fetal ovaries from SPF and ABX dams. Notably, given the extremely small size of fetal ovarian tissue, approximately 30 ovaries were pooled together for each group to

reach the lowest detection threshold. To minimize confounding factors such as variation in ovarian size, the final bile acid concentrations were normalized by total cell number for each group (**Table EV2**), which we believe will ensure more accurate conclusions. The results revealed that 29 out of the total 31 detected bile acids showed reduced abundance in the fetal ovaries of ABX dams compared to SPF dams (**Fig. EV4E**). Although the differences in bile acid concentrations appeared modest at the per-cell level (log2FC range from 0–0.4), it is important to note that the fetal ovary contains a large number of cells; therefore, even minor alterations in bile acid levels at the cellular level may contribute substantial differences at the whole ovary level. Together, these data suggest that maternal microbiome disruption-induced alterations in bile acid metabolism in the maternal serum are reflected within the fetal ovary.

Appendix Figure EV4: *(E) Bar chart illustrating the Log2 fold change (SPF/ABX) of 31 detected bile acids in fetal ovaries from SPF and ABX dams at E14.5. For each group, at least 30 ovaries were included for analysis and the final concentrations were normalized to cell number.*

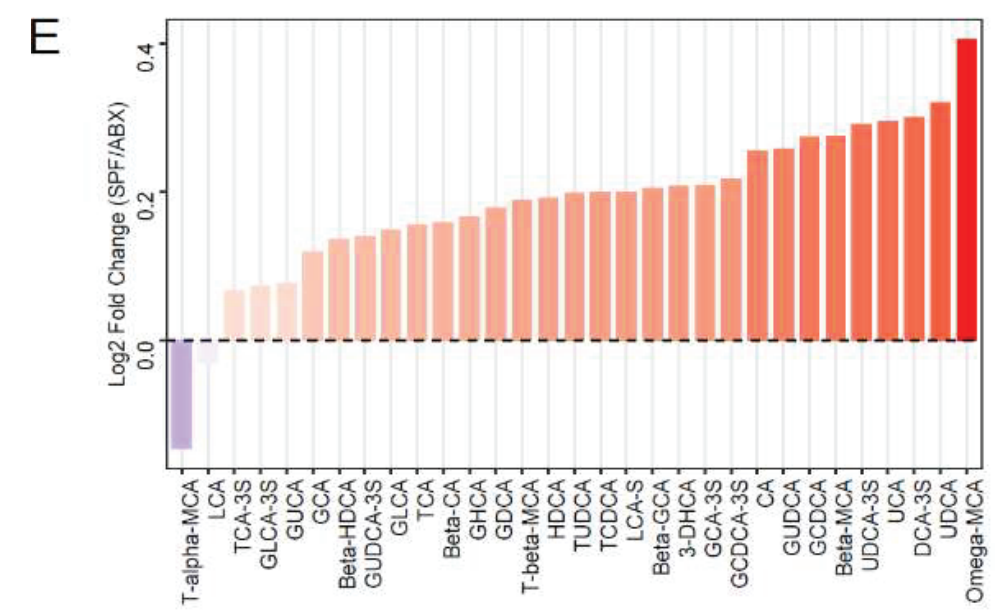

Regarding the recovery of TDCA and GUDCA, we would like to note that this discrepancy may be attributable to differences between experimental models. Supporting such a hypothesis, according to our supplemented relative bacterial biomass abundance of intestinal contents from SPF (Before ABX), ABX (After ABX) and ABX (E14.5 dams) groups following 12 h of in vitro culture (**see Minor Concerns, Point 2**), antibiotic-induced maternal microbiome disruption could persist until E14.5 (**Fig. EV1B,C; $p < 0.001$**). Furthermore, our targeted metabolomic data from both maternal serum and fetal ovarian tissue consistently support the conclusion that ABX mice showed reduced bile acid abundance at E14.5. Taken together, these findings suggest that the observed DOR phenotype in the ABX group is at least partially bile acid-mediated.

Minor concerns:

1. Fig 1A, it seems that ABX is administered in utero between E6.5-E16.5, I find this confusing. If they treat the mice with ABX during this period, authors should clearly discuss this in the manuscript and method section as well. If this is not clarified well, the use of other non-absorable ABX might be recommended for this manuscript to exclude metronidazole's direct genotoxic effects. It is also important to note that ABX residues can also be found in mouse feces after ABX termination, causing mice to continue to be exposed to ABX after mating, unless the cage is changed with a new bedding. It would be useful if you could clarify this in the method section and remove the confustion from Fig 1A.

Response: We thank the reviewer for this careful and important observation. We sincerely apologize for the ambiguity in the original Figure 1A, which we recognize may have conveyed the misleading information that antibiotic (ABX) treatment was administered during the E6.5–E16.5 gestational window. Actually, the letter "ABX" on the colored bars represents experimental group information, rather than antibiotic treatment. To avoid any potential misunderstanding, we have supplemented essential information in the Materials and Methods section of our revised manuscript. Furthermore, we have updated our Figure 1A in our revised manuscript. Specifically, we have removed group information from the colored bars and annotated related group information to precede each experimental group. We believe the revised Figure 1A is now clearly presented to the readers.

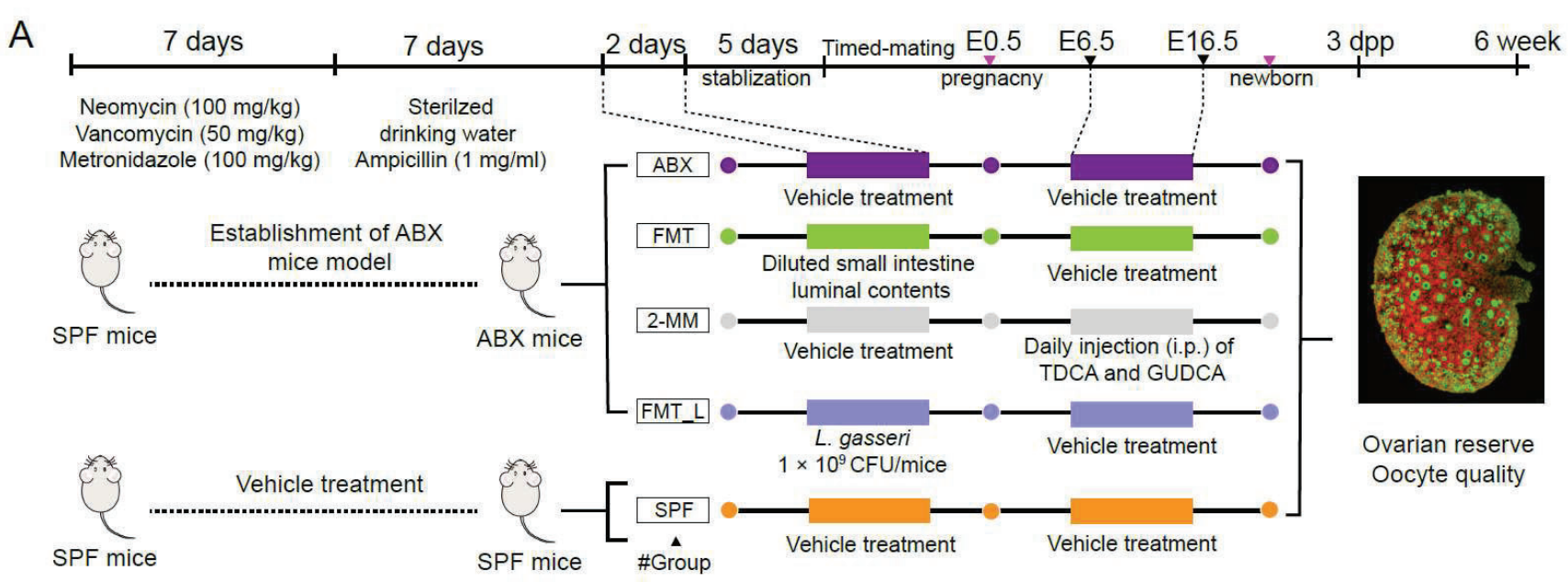

Fig. 1A: *(A) Brief timeline diagram of the experimental procedure.*

2. Based on the microbiome abundance levels shown in supp Fig 1A-C, it is not clear what time point they represent. Authors should include abundance data prior to ABX, right after ABX termination, and at E14.5 recovery. If the mice are kept in the same cage, diet and environment, their gut microbiome usually recovers faster, so it is important to show the relative abundance before and after ABX termination.

Response: We thank the reviewer for pointing this out. For supplementary Fig. 1A-C, the relative bacterial biomass density was analyzed using intestinal contents after antibiotics administration, and before conception. In our revised maniuscript, the related information has been added both in the main text and corresponding figure legends. Furthmore, we include abundance data prior to ABX, right after ABX termination, and at E14.5 recovery as the reviewer suggested (**Fig. EV1B,C**).

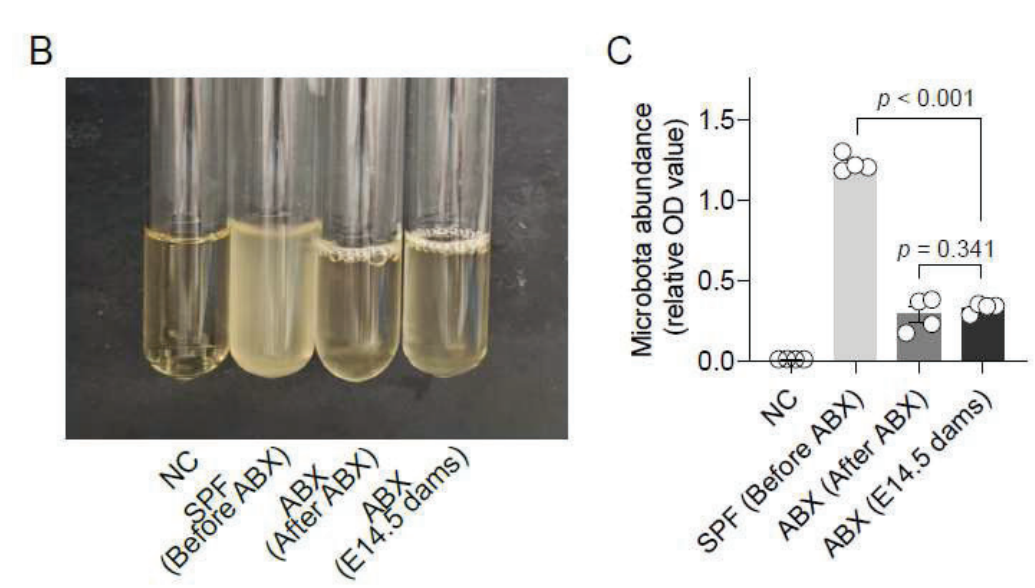

Appendix Figure EV1. *(B) Representative images of bacterial cultures from small intestine luminal contents in ABX mice before antibiotic administration, after antibiotic administration, and at E14.5 during gestation. Images were captured after 12 h of in vitro culture. (C) Relative bacterial biomass abundance of intestinal contents from SPF (Before ABX), ABX (After ABX) and ABX (E14.5 dams) groups following 12 h of in vitro culture. Data was quantified using a spectrophotometer at*

595 nm.

The results showed that antibiotic-induced maternal microbiome disruption could persist until E14.5, further verifying the effectiveness of antibiotic-induced disruption during pregnancy. The lasting effects of antibiotic-induced maternal microbiome disruption in our research is reasonable since that fact that the female mice were housed individually in cages with autoclaved bedding. Moreover, all mice were fed with 60Co-γ irradiation and high temperature-sterilized chow and autoclaved water. We believe that only under such strict housing conditions can we successfully establish a maternal microbiome dysbiosis model for this study.

3. Why are only germline cells susceptible to DNA damage? How about the surrounding somatic cells? What protects somatic cells? Fig 2H showing γH2AX co-localized with DDX4 signals, indicating germ cell populations - we can't distinguish somatic from germline cells at this resolution, more clear magnification is needed.

Response: We thank the reviewer for raising this important and insightful question. We sincerely apologize for the insufficient resolution of the original figure. In our revised manuscript, we have updated **Figure 2H**, **Figure 3A**, and **Figure 5B** with high-resolution images accordingly. Specifically, to ensure a clearer localization of the γH2AX signals in **Figure 2H**, we provide magnified split-channel images alongside the whole tissue images, which clearly show the colocalization of γH2AX and DDX4 signals.

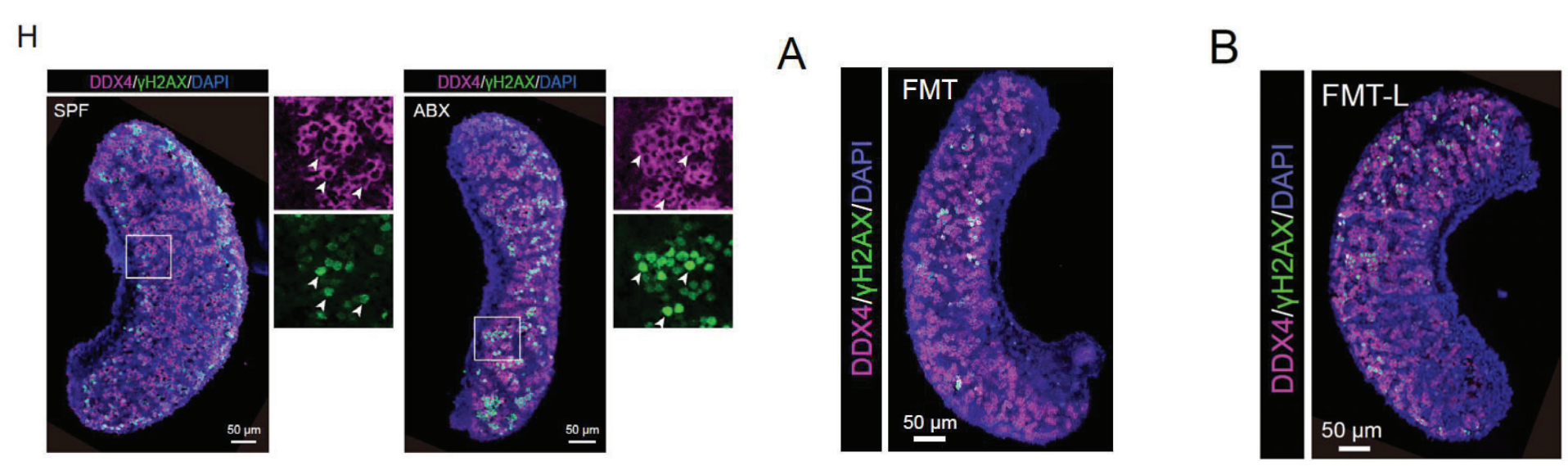

Revised Figure 2H, Figure 3A, and Figure 5B.

Regarding why only germline cells are susceptible to DNA damage, we propose that the progression of the meiotic program in germ cells, but not surrounding somatic cells, may account for this phenomenon. Mechanistically, female germ cells initiate the meiotic program at E13.5, during which SPO11 induces large-scale programmed DNA double-strand breaks (DSBs) to promote homologous chromosome pairing and crossover formation (1). Following that, these DSBs were then repaired by homologous recombination-associated factors (such as RAD51, etc.) to ensure the formation of crossover, a process that is essential for homologous recombination (2). Specifically, substantial studies have demonstrated that female germ cells en route to the meiotic program in utero are vulnerable to environmental perturbations, such as chemotherapeutic drugs, endocrine-disrupting chemicals, and irradiation, which similarly lead to ovarian reserve depletion (3). Together, this intrinsic mechanism may

account for why only germline cells showed elevated γH2AX signals but not surrounding somatic cells.

Reference:

1. *Shin Y H, McGuire M M, Rajkovic A. Mouse HORMAD1 is a meiosis checkpoint protein that modulates DNA double-strand break repair during female meiosis[J]. Biology of reproduction, 2013, 89(2): 29, 1-12.*
2. *Cohen P E, Pollack S E, Pollard J W. Genetic analysis of chromosome pairing, recombination, and cell cycle control during first meiotic prophase in mammals[J]. Endocrine reviews, 2006, 27(4): 398-426.*
3. *Richardson MC, Guo M, Fauser BC, Macklon NS. Environmental and developmental origins of ovarian reserve[J]. Hum Reprod Update, 2014, May-Jun;20(3):353-69.*

4. In Figure 6A there are some images that don't match with authors claim, especially if you count from 2-cells to blastocyts, it lacks consistency in numbers e.g. FMT, on top of this the FMT blastocyts image looks more affected than the ABX blastocyt images. IVF experiments are quite noisy, if they are to be convincing, they need multiple experiments, and qualitative data taken from different IVF experiments rather than the same IVF experiment makes the data less convincing. Authors are strongly advised to put clear, nice and quantitatively consistent number of embroys figures to minimize reader confusion.

Response: Thank the reviewer for pointing this out. In our original manuscript, the representative images were randomly selected, which may have caused inconsistency in our quantitative analysis. As the reviewer suggested, we have replaced the images that may cause any confusion in our revised manusctipt as the reviewer suggested.

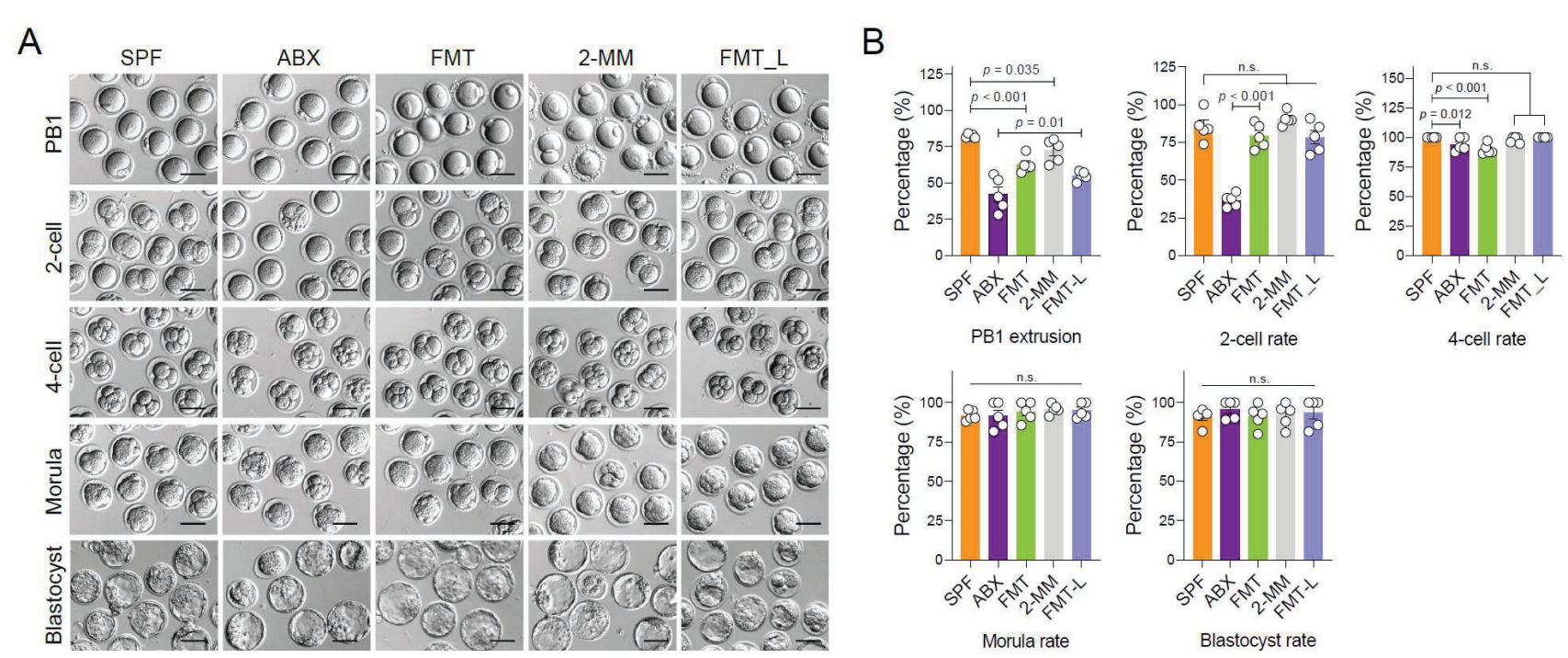

Figure 6: *(A) Representative images of PB1 and early embryos using oocytes collected from offspring of the SPF, ABX, FMT, 2-MM, and FMT_L dams. Scale bars: 100 µm. (B) Comparative analysis of PB1, fertilization, 4-cell, morula, and blastocyst rates using oocytes collected from offspring of the SPF, ABX, FMT, 2-MM, and FMT_L dams. At least five independent repeats were included for each group.*

Furthermore, the reviewer is right that the IVF experiments are quite noisy, and the qualitative data should be taken from different IVF experiments rather than the same

IVF experiment. In our original analysis, the quantitative analysis in Figure 6B was collected from different batches of experiments, but not the same IVF experiment, to maximize reliability and minimize batch-specific effects in our analysis. We have supplemented related information to our revised manuscript to minimize reader confusion. Thank you.

5. Line 400 " While our findings here support the conclusion that periconceptional antibiotic-mediated disruption of the maternal microbiome induces diminished ovarian reserve in offspring". This conclusion is hardly true for two reasons: First, matured oocytes are not affected in F0 mothers, and second, the dysbiotc condition remains even after mating, thus E14.5 metabolomics profile reveals a difference and E6.5-E16.5 supplements restored the phenotype. To conclude this is a periconceptional effect, either the antibiotic-mediated disruption must be fully recovered before mating, or one needs to perform IVF and transfer the 2-cell embryos to a pseudopregnant CD1 foster mother that are not exposed to ABX, followed by a gonads (germline) check at E14.5 and P3. In any case, if the dysbiosis in pregnant F0 dams is not completely recovered during pregnancy, the effects could be additive during and after pregnancy, but the evidence presented in this manuscript (F1 germline) supports that this effect is more likely to occur due to prolonged in-utero dysbiosis than periconceptional dysbiosis alone. In the manuscript, the author should remove the word "periconceptional line 348/400" and replace it with "maternal gut dysbiosis" or "pervasive maternal gut perturbations".

Response: We thank the reviewer for this insightful and constructive comment. We agree with the reviewer that maternal gut dysbiosis would be more accurate than the word “periconceptional.” We have revised the manuscript to remove the term “periconceptional” and replaced it with “maternal gut dysbiosis” as the reviewer suggested.

Once again, we would like to thank the editor and the reviewers for the helpful comments.

Wei Ge

Wei Ge, Ph.D.

College of Animal Science and Technology

Qingdao Agricultural University

Dear Dr. Ge

Thank you for the submission of your revised manuscript to EMBO reports. As my colleague Achim Breiling is currently traveling, I have temporarily taken over the handling of your manuscript. We have now received the report from the arbitrator who is very positive about your study and recommends publication.

Browsing through the manuscript myself, I noticed a few editorial things that we need before we can proceed with the official acceptance of your study.

- We noticed the following author name discrepancy, which needs to be resolved: Yiling Niu in the manuscript vs. Yi-Lin Niu in the online submission system.

- The references need to be alphabetical instead of numerical; et al needs to be used after 10 author names; DOIs should only be used for preprints and datasets that have not been published yet.

- Please provide a complete author checklist, which you can download from our author guidelines. Please insert information in the checklist that is also reflected in the manuscript. The completed author checklist will also be part of the RPF.

- Information on funding: in the online system the funders and grant numbers provided in the Comments box need to be removed from the box and entered as separate funders above (More Funders option). In the manuscript, the funding information needs to be provided in the Acknowledgments section. The separate Funding title should therefore be removed.

- EV tables:
* Table EV1 is a complex dataset and needs to be updated to Dataset EV1. Please change the nomenclature also in the file itself (legend in the .xls sheet) and update all callouts accordingly.
* Table EV5 could be part of the Reagents and Tools table. I think the information on antibodies is already listed in the Reagents table, isn't it? Please also add the RIDD identifiers in the Reagents table.
* Please note that the title of Table EV6 wrongly refers to Table EV5. We further noticed a typo in the title of Table EV2 (Ttable).
* Please remvoe the legends of the EV Tables from the manuscript. It is sufficient to have these in the .xls files themselves.

- Figure legends:
* Please indicate the statistical test used for data analysis in the legends of 1B,C,E,G; 2F-I; 3B,D,F; 4B,E,G,I; 5C,E,G; 6B,E,G; EV-1C,E,F,H,J; EV-3; EV-4B; EV-6B,D.
* Please provide information related to n in the legends 1B; 2F,I; 3B; 4E; 5C; 6E,G; EV-1C,E; EV-2A,F,H,K; EV-4B.
* Please define the error bars in the legends of figures EV-2F,H,K; EV-3.

- Please define the scale bar size only in the legends not in the figure panels.

- The title for the main figure legends should be "Figure legends".

- Source data: for Figure 4A you note that the data have been deposited. This refers to the metabolomics data. Have these (can they) be deposited on a public repository?

- Finally, EMBO Reports papers are accompanied online by
A) a short (1-2 sentences) summary of the findings and their significance,
B) 2-3 bullet points highlighting key results and
C) a schematic summary figure that provides a sketch of the major findings (not a data image).
Please provide the summary figure as a separate file in PNG or JPG format at a size of 550x300 pixels (width x height). Please note that the size is rather small and that text needs to be readable at the final size. Please send us this information along with the revised manuscript.

I look forward to seeing a new revised version of your manuscript as soon as possible. Please use this link to submit your revision: https://embor.msubmit.net/cgi-bin/main.plex

Kind regards,

Martina Rembold, PhD
Senior Editor
EMBO reports

==================

Referee #1/Arbitrator

Dear Editors,

The authors have adequately addressed my concerns and I recommend the manuscript for publication.

Kind regards,

Ayele

Point-by-point response

Title: Maternal microbiome promotes offspring ovarian reserve via bile acid metabolism

No: EMBOR-2026-63683V3

EMBO reports

First, we would like to thank the editors and the reviewer very much for their helpful comments.

Referee #1/Arbitrator:

The authors have adequately addressed my concerns and I recommend the manuscript for publication.

Response: We thank the reviewer for the very positive evaluation of our work. The reviewer's constructive comments have substantially improved the quality of this manuscript.

Editors:

1. We noticed the following author name discrepancy, which needs to be resolved: Yiling Niu in the manuscript vs. Yi-Lin Niu in the online submission system.

Response: Thank you for pointing this out, the correct name should be Yilin Niu, we have correct this in our revised manuscript.

2. The references need to be alphabetical instead of numerical; et al needs to be used after 10 author names; DOIs should only be used for preprints and datasets that have not been published yet.

Response: We have revised our references accordingly.

3. Please provide a complete author checklist, which you can download from our author guidelines. Please insert information in the checklist that is also reflected in the manuscript. The completed author checklist will also be part of the RPF.

Response: We have supplemented this file accordingly.

4. Information on funding: in the online system the funders and grant numbers provided in the Comments box need to be removed from the box and entered as separate funders above (More Funders option). In the manuscript, the funding information needs to be provided in the Acknowledgments section. The separate Funding title should therefore be removed.

Response: We have revised this in the the online system accordingly.

EV tables:

1. Table EV1 is a complex dataset and needs to be updated to Dataset EV1. Please change the nomenclature also in the file itself (legend in the .xls sheet) and update all callouts accordingly.

Response: We have revised this accordingly.

2. Table EV5 could be part of the Reagents and Tools table. I think the information on antibodies is already listed in the Reagents table, isn't it? Please also add the RIDD identifiers in the Reagents table.

Response: Thank you for pointing this out. In the revised manuscript, we have removed Table EV5 and supplemented the corresponding RIDD identifiers to the Reagents and Tools table.

3. Please note that the title of Table EV6 wrongly refers to Table EV5. We further noticed a typo in the title of Table EV2 (Ttable).

Response: Thank you for pointing this out, we have revised this accordingly.

4. Please remvoe the legends of the EV Tables from the manuscript. It is sufficient to have these in the .xls files themselves.

Response: We have revised this accordingly.

Figure legends:

1. Please indicate the statistical test used for data analysis in the legends of 1B,C,E,G; 2F-I; 3B,D,F; 4B,E,G,I; 5C,E,G; 6B,E,G; EV-1C,E,F,H,J; EV-3; EV-4B; EV-6B,D.

Response: We have supplemented related information accordingly.

2. Please provide information related to n in the legends 1B; 2F,I; 3B; 4E; 5C; 6E,G; EV-1C,E; EV-2A,F,H,K; EV-4B.

Response: We have supplemented related information accordingly.

3. Please define the error bars in the legends of figures EV-2F,H,K; EV-3.

Response: We have supplemented related information accordingly.

4. Please define the scale bar size only in the legends not in the figure panels.

Response: We have revised this accordingly.

5. The title for the main figure legends should be "Figure legends".

Response: We have revised this accordingly.

6. Source data: for Figure 4A you note that the data have been deposited. This refers to the metabolomics data. Have these (can they) be deposited on a public repository?

Response: The source file were provided in Dataset EV1. Thank you.

7. Finally, EMBO Reports papers are accompanied online by
A) a short (1-2 sentences) summary of the findings and their significance,
B) 2-3 bullet points highlighting key results and
C) a schematic summary figure that provides a sketch of the major findings (not a data image).
Please provide the summary figure as a separate file in PNG or JPG format at a size of 550x300 pixels (width x height). Please note that the size is rather small and that text needs to be readable at the final size. Please send us this information along with the revised manuscript.

Response: We have supplemented related information in our revised manuscript accordingly. A short (1-2 sentences) summary of the findings and their significance has been added to the revised manuscript right after the abstract, and the Synopsis image has also been uploaded via the submission system.

Once again, we would like to thank the editor and the reviewers for the helpful comments.

Wei Ge

Wei Ge, Ph.D.

College of Animal Science and Technology

Qingdao Agricultural University

2nd Revision - Editorial Decision **13th Aug 2026**

Dr. Wei Ge
Qingdao Agricultural University
College of Animal Science and Technology
China

Dear Dr. Ge,

I am very pleased to accept your manuscript for publication in the next available issue of EMBO reports. Thank you for your contribution to our journal.

Your manuscript will be processed for publication by EMBO Press. It will be copy edited and you will receive page proofs prior to publication. Please note that you will be contacted by Springer Nature Author Services to complete licensing and payment information.

You may qualify for financial assistance for your publication charges - either via a Springer Nature fully open access agreement or an EMBO initiative. Check your eligibility: https://link.springer.com/journal/44319/how-to-publish-with-us

Should you be planning a Press Release on your article, please notify the production team via this form in order to coordinate publication and release dates: https://forms.cloud.microsoft/e/eH4A8JVP6g

If you have any questions, please do not hesitate to contact the Editorial Office. Thank you for your contribution to EMBO Reports.

Yours sincerely,

Achim Breiling
Senior Editor
EMBO Reports

>>> Please note that it is EMBO Reports policy for the transcript of the editorial process (containing referee reports and your response letter) to be published as an online supplement to each paper. If you do NOT want this, you will need to inform the Editorial Office via email immediately. More information is available here: https://link.springer.com/partners/embo-press/editorial-policies#Peer%20review

EMBO Press Author Checklist

Corresponding Author Name: Wei Ge	
Journal Submitted to: EMBO Reports	
Manuscript Number: EMBOR-2026-63683V3	

USEFUL LINKS FOR COMPLETING THIS FORM
The EMBO Journal - Author Guidelines
EMBO Reports - Author Guidelines
Molecular Systems Biology - Author Guidelines
EMBO Molecular Medicine - Author Guidelines

Reporting Checklist for Life Science Articles (updated January
This checklist is adapted from Materials Design Analysis Reporting (MDAR) Checklist for Authors. MDAR establishes a minimum set of requirements in transparent reporting in the life sciences (see Statement of Task: 10.31222/osf.io/9sm4x). Please follow the journal's guidelines in preparing your
Please note that a copy of this checklist will be published alongside your article.

Abridged guidelines for figures
1. Data
The data shown in figures should satisfy the following conditions:
- the data were obtained and processed according to the field’s best practice and are presented to reflect the results of the experiments in an accurate and unbiased manner.
- ideally, figure panels should include only measurements that are directly comparable to each other and obtained with the same assay.
- plots include clearly labeled error bars for independent experiments and sample sizes. Unless justified, error bars should not be shown for technical
- if n<5, the individual data points from each experiment should be plotted. Any statistical test employed should be justified.
- Source Data should be included to report the data underlying figures according to the guidelines set out in the authorship guidelines on Data

2. Captions
Each figure caption should contain the following information, for each panel where they are relevant:
- a specification of the experimental system investigated (eg cell line, species name).
- the assay(s) and method(s) used to carry out the reported observations and measurements.
- an explicit mention of the biological and chemical entity(ies) that are being measured.
- an explicit mention of the biological and chemical entity(ies) that are altered/varied/perturbed in a controlled manner.
- the exact sample size (n) for each experimental group/condition, given as a number, not a range;
- a description of the sample collection allowing the reader to understand whether the samples represent technical or biological replicates (including how many animals, litters, cultures, etc.).
- a statement of how many times the experiment shown was independently replicated in the laboratory.
- definitions of statistical methods and measures:
  - common tests, such as t-test (please specify whether paired vs. unpaired), simple χ2 tests, Wilcoxon and Mann-Whitney tests, can be unambiguously identified by name only, but more complex techniques should be described in the methods section;
  - are tests one-sided or two-sided?
  - are there adjustments for multiple comparisons?
  - exact statistical test results, e.g., P values = x but not P values < x;
  - definition of ‘center values’ as median or average;
  - definition of error bars as s.d. or s.e.m.

Please complete ALL of the questions below.
Select "Not Applicable" only when the requested information is not relevant for your study.

Materials

Newly Created Materials	Information included in the manuscript?	In which section is the information available? (Reagents and Tools Table, Materials and Methods, Figures, Data Availability Section)
New materials and reagents need to be available; do any restrictions apply?	Not Applicable	

Antibodies	Information included in the manuscript?	In which section is the information available? (Reagents and Tools Table, Materials and Methods, Figures, Data Availability Section)
For **antibodies** provide the following information:<br>- Commercial antibodies: RRID (if possible) or supplier name, catalogue number and or/clone number<br>- Non-commercial: RRID or citation	Yes	Reagents and Tools Table, Materials and Methods

DNA and RNA sequences	Information included in the manuscript?	In which section is the information available? (Reagents and Tools Table, Materials and Methods, Figures, Data Availability Section)
**Short novel DNA or RNA including primers, probes**: provide the sequences.	Yes	Table EV4

Cell materials	Information included in the manuscript?	In which section is the information available? (Reagents and Tools Table, Materials and Methods, Figures, Data Availability Section)
**Cell lines:** Provide species information, strain. Provide accession number in repository **OR** supplier name, catalog number, clone number, and/**OR** RRID.	Not Applicable	
**Primary cultures:** Provide species, strain, sex of origin, genetic modification status.	Not Applicable	
Report if the cell lines were recently **authenticated** (e.g., by STR profiling) and tested for mycoplasma contamination.	Not Applicable	

Experimental animals	Information included in the manuscript?	In which section is the information available? (Reagents and Tools Table, Materials and Methods, Figures, Data Availability Section)
**Laboratory animals or Model organisms:** Provide species, strain, sex, age, genetic modification status. Provide accession number in repository **OR** supplier name, catalog number, clone number, **OR** RRID.	Yes	Reagents and Tools Table, Materials and Methods
**Animal observed in or captured from the field:** Provide species, sex, and age where possible.	Not Applicable	
Please detail **housing and husbandry conditions**.	Yes	Materials and Methods

Plants and microbes	Information included in the manuscript?	In which section is the information available? (Reagents and Tools Table, Materials and Methods, Figures, Data Availability Section)
**Plants:** provide species and strain, ecotype and cultivar where relevant, unique accession number if available, and source (including location for collected wild specimens).	Not Applicable	
**Microbes:** provide species and strain, unique accession number if available, and source.	Yes	Reagents and Tools Table

Human research participants	Information included in the manuscript?	In which section is the information available? (Reagents and Tools Table, Materials and Methods, Figures, Data Availability Section)
If collected and within the bounds of privacy constraints report on age, sex and gender or ethnicity for all study participants.	Not Applicable	

Core facilities	Information included in the manuscript?	In which section is the information available? (Reagents and Tools Table, Materials and Methods, Figures, Data Availability Section)
If your work benefited from core facilities, was their service mentioned in the acknowledgments section?	Not Applicable	

Design

Study protocol	Information included in the manuscript?	In which section is the information available? (Reagents and Tools Table, Materials and Methods, Figures, Data Availability Section)
If study protocol has been **pre-registered, provide DOI in the manuscript**. For clinical trials, provide the trial registration number **OR** cite DOI.	Not Applicable	
Report the **clinical trial registration number** (at ClinicalTrials.gov or equivalent), where applicable.	Not Applicable	

Laboratory protocol	Information included in the manuscript?	In which section is the information available? (Reagents and Tools Table, Materials and Methods, Figures, Data Availability Section)
Provide DOI OR other citation details if **external detailed step-by-step protocols** are available.	Yes	Materials and Methods

Experimental study design and statistics	Information included in the manuscript?	In which section is the information available? (Reagents and Tools Table, Materials and Methods, Figures, Data Availability Section)
Include a statement about **sample size** estimate even if no statistical methods were used.	Yes	Figures
Were any steps taken to minimize the effects of subjective bias when allocating animals/samples to treatment (e.g. **randomization procedure**)? If yes, have they been described?	Yes	Materials and Methods
Include a statement about **blinding** even if no blinding was done.	Yes	Materials and Methods
Describe **inclusion/exclusion criteria** if samples or animals were excluded from the analysis. Were the criteria pre-established?<br><br>If sample or data points were omitted from analysis, report if this was due to attrition or intentional exclusion and provide justification.	Not Applicable	
For every figure, are **statistical tests** justified as appropriate? Do the data meet the assumptions of the tests (e.g., normal distribution)? Describe any methods used to assess it. Is there an estimate of variation within each group of data? Is the variance similar between the groups that are being statistically compared?	Yes	Figures

Sample definition and in-laboratory replication	Information included in the manuscript?	In which section is the information available? (Reagents and Tools Table, Materials and Methods, Figures, Data Availability Section)
In the figure legends: state number of times the experiment was **replicated** in laboratory.	Yes	Figures
In the figure legends: define whether data describe **technical or biological replicates**.	Yes	Figures

Ethics

Ethics	Information included in the manuscript?	In which section is the information available? (Reagents and Tools Table, Materials and Methods, Figures, Data Availability Section)
Studies involving **human participants**: State details of **authority granting ethics approval** (IRB or equivalent committee(s), provide reference number for approval.	Not Applicable	
Studies involving **human participants**: Include a statement confirming that **informed consent** was obtained from all subjects and that the experiments conformed to the principles set out in the WMA Declaration of Helsinki and the Department of Health and Human Services Belmont Report.	Not Applicable	
Studies involving **human participants:** For publication of **patient photos**, include a statement confirming that consent to publish was obtained.	Not Applicable	
Studies involving experimental **animals**: State details of **authority granting ethics approval** (IRB or equivalent committee(s), provide reference number for approval. Include a statement of compliance with ethical regulations.	Yes	Materials and Methods
Studies involving **specimen and field samples:** State if relevant **permits** obtained, provide details of authority approving study; if none were required, explain why.	Not Applicable	

Dual Use Research of Concern (DURC)	Information included in the manuscript?	In which section is the information available? (Reagents and Tools Table, Materials and Methods, Figures, Data Availability Section)
Could your study fall under dual use research restrictions? Please check biosecurity documents and list of **select agents and toxins** (CDC): https://www.selectagents.gov/sat/list.htm	Not Applicable	
If you used a select agent, is the security level of the lab appropriate and reported in the manuscript?	Not Applicable	
If a study is subject to dual use research of concern regulations, is the name of the **authority granting approval and reference number** for the regulatory approval provided in the manuscript?	Not Applicable	

Reporting

The MDAR framework recommends adoption of discipline-specific guidelines, established and endorsed through community initiatives. Journals have their own policy about requiring specific guidelines and recommendations to complement MDAR.

Adherence to community standards	Information included in the manuscript?	In which section is the information available? (Reagents and Tools Table, Materials and Methods, Figures, Data Availability Section)
State if relevant guidelines or checklists (e.g., **ICMJE, MIBBI, ARRIVE, PRISMA**) have been followed or provided.	Not Applicable	
For **tumor marker prognostic studies,** we recommend that you follow the **REMARK** reporting guidelines (see link list at top right). See author guidelines, under 'Reporting Guidelines'. Please confirm you have followed these guidelines.	Not Applicable	
For **phase II and III randomized controlled trials**, please refer to the **CONSORT** flow diagram (see link list at top right) and submit the CONSORT checklist (see link list at top right) with your submission. See author guidelines, under 'Reporting Guidelines'. Please confirm you have submitted this list.	Not Applicable	

Data Availability

Data availability	Information included in the manuscript?	In which section is the information available? (Reagents and Tools Table, Materials and Methods, Figures, Data Availability Section)
Have **primary datasets** been deposited according to the journal's guidelines (see 'Data Deposition' section) and the respective accession numbers provided in the Data Availability Section?	Yes	Data Availability Section
Were **human clinical and genomic datasets** deposited in a public access-controlled repository in accordance to ethical obligations to the patients and to the applicable consent agreement?	Not Applicable	
Are **computational models** that are central and integral to a study available without restrictions in a machine-readable form? Were the relevant accession numbers or links provided?	Not Applicable	
If publicly available data were reused, provide the respective **data citations in the reference list**.	Yes	Data Availability Section
